# De Novo Design of Membrane‐Targeting Antimicrobial Peptides Against Gram‐Negative Bacteria Using a Generative Artificial Intelligence Framework

**DOI:** 10.1002/advs.75928

**Published:** 2026-06-02

**Authors:** Jingxiao Yu, Da‐Wen Sun, QingYi Wei, Yibing Zhang, Jidong Tang, Hongbin Pu

**Affiliations:** ^1^ School of Food Science and Engineering South China University of Technology Guangzhou China; ^2^ Academy of Contemporary Food Engineering South China University of Technology Guangzhou Higher Education Mega Centre Guangzhou China; ^3^ Engineering and Technological Research Centre of Guangdong Province On Intelligent Sensing and Process Control of Cold Chain Foods & Guangdong Province Engineering Laboratory For Intelligent Cold Chain Logistics Equipment for Agricultural Products Guangzhou Higher Education Mega Centre Guangzhou China; ^4^ Food Refrigeration and Computerized Food Technology (FRCFT) Agriculture and Food Science Centre University College Dublin National University of Ireland Belfield Ireland

**Keywords:** antimicrobial activity and biosafety, antimicrobial mechanism, antimicrobial peptides, generative artificial intelligence framework, gram‐negative bacteria

## Abstract

Antimicrobial resistance (AMR), particularly Gram‐negative bacteria, poses significant challenges due to their robust outer membranes limiting antibiotic efficacy. Antimicrobial peptides (AMPs) show promising potential to replace traditional antibiotics. This study proposes a multi‐condition constrained directed generation framework guided by the AMP antimicrobial mechanisms for designing membrane‐targeting antimicrobial peptides (MTAMPs) against Gram‐negative bacteria. By integrating sequence information, physicochemical properties, and spatial structure (PCSS) descriptors related to the outer membrane, a conditional variational autoencoder (GenMTAMP) model was developed for de novo MTAMP design. Then, target PCSS descriptors within a predefined range are input as conditional constraints into the GenMTAMP model to direct generate MTAMPs. Candidate MTAMPs were screened and evaluated through subsequent identification (ClaAMP) and prediction (PreAMP) modules. Experimental validation showed that two top‐ranked peptides named MTAMP003 and MTAMP004, exhibit excellent antibacterial activity against Gram‐negative bacteria while maintaining low cytotoxicity and haemolytic activity toward mammalian cells. Furthermore, mechanism research indicates MTAMPs can disrupt the Gram‐negative bacterial outer membrane barrier, with limited impact on mammalian cell membranes. In summary, the current research establishes a targeted and efficient generative artificial intelligence (AI) framework for de novo MTAMP design, and provides a generalisable framework for the rational design of AMPs with predefined functional properties.

## Graphical abstract

Antimicrobial resistance caused by Gram‐negative bacteria remains difficult to overcome due to the protective outer membrane. To address this challenge, a multi‐condition constrained generative AI framework, GenMTAMP is proposed for de novo membrane‐targeting antimicrobial peptide design by integrating physicochemical and spatial structure descriptors. The generated peptides show potent antibacterial activity, low toxicity, and haemolysis to mammals, demonstrating promising potential for rational AMP design.

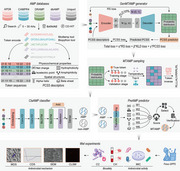

## 1 Introduction

According to data from the World Health Organization (WHO), antimicrobial resistance (AMR) is one of the major global health threats in the 21st century [1]. Related research predicts that 19.1 million people will die from AMR by 2050 [2]. Compared to Gram‐positive bacteria, Gram‐negative bacteria are more susceptible to developing AMR, primarily due to characteristics such as outer membrane barrier, highly efficient efflux pump systems, and robust horizontal gene transfer capabilities. Notably, the outer membrane is the primary and most formidable defence barrier for Gram‐negative bacteria [3]. Therefore, there is an urgent need for current research to explore novel antimicrobial agents to replace traditional antibiotics and effectively inhibit resistance development.

Antimicrobial peptides (AMPs) are a class of short‐chain polypeptide molecules comprising approximately 5 to 50 amino acids (AAs) [4]. Due to targeting the outer membrane, it is difficult for Gram‐negative bacteria to develop resistance to AMPs. Compared to traditional antibiotics, AMPs exhibit lower resistance risks, stronger membrane penetration capabilities, broader antimicrobial activity, and faster bactericidal rates [5]. Currently, methods for discovering natural AMPs face numerous limitations, including complex purification processes, extended timelines, high costs, restricted sources, and innovation challenges. Consequently, it is necessary to develop more efficient and economical AMP discovery technologies.

Artificial intelligence (AI) can intelligently screen or design AMPs by collecting data, processing information, and establishing models [6]. In recent years, with the continuous expansion of AMP databases and the continuous optimization of generative models, research on designing AMPs through AI have achieved significant progress [7], such as the AlphaFold2 model [8]. Compared to existing database screening approaches [9], AMP generation models exhibit lower data dependency, greater sequence novelty, and richer functional characteristics [10]. Currently, generative models such as the variational autoencoder (VAE) [11], the diffusion model [12], the large language model [13], and the graph neural network [14] have been employed to de novo design AMPs. The conditional variational autoencoder (CVAE) can regulate latent space through explicit conditional constraints, achieving directed generation and multi‐objective optimization of AMP sequences. Therefore, there is great potential in developing multi‐condition constrained generation models tailored to the characteristics of Gram‐negative bacterial outer membranes, which can significantly enhance the targeting specificity of generated AMPs.

**FIGURE 1:**
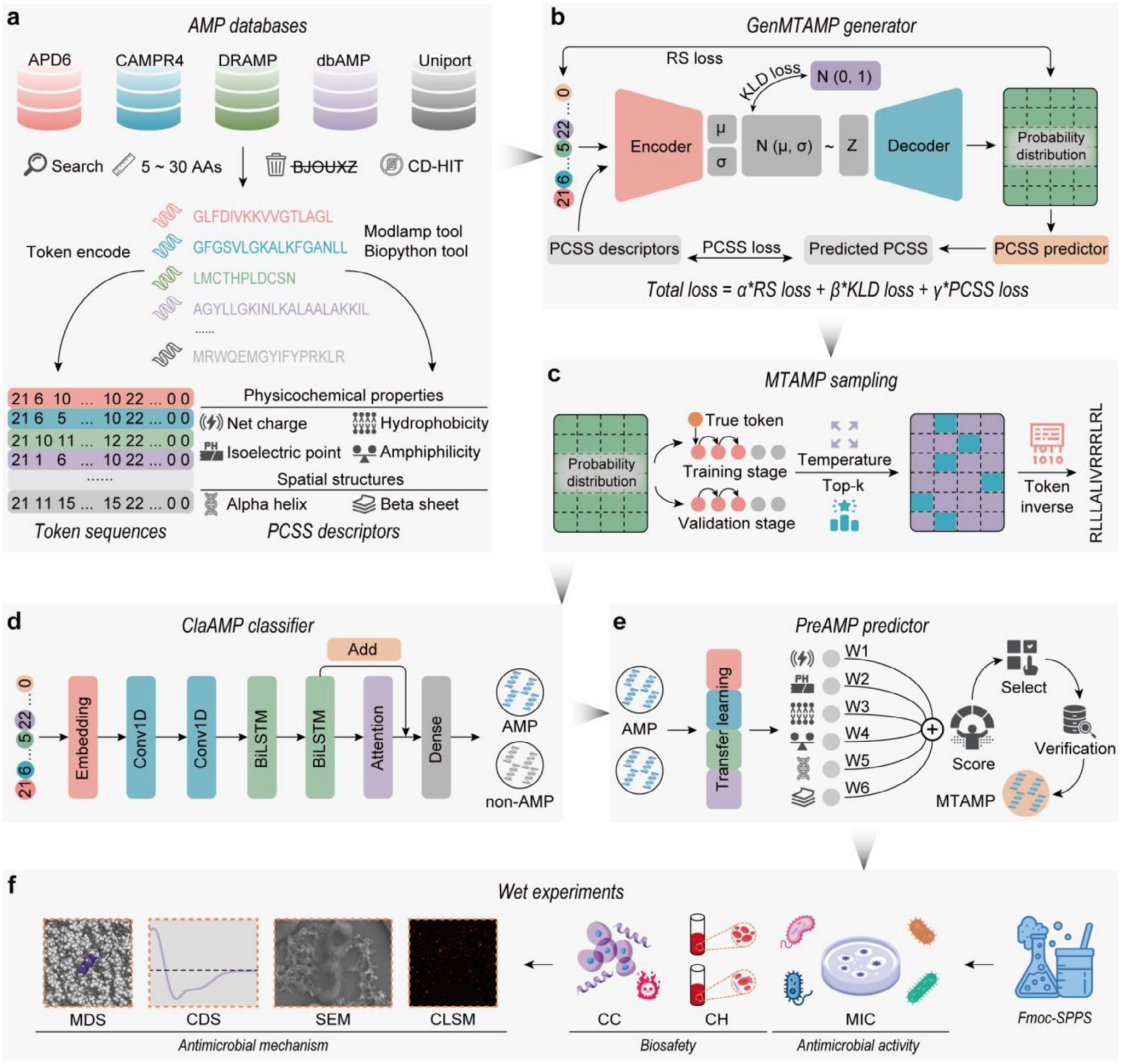
The pipeline framework of the current research. (a) The AMP and non‐AMP sequence data obtained from five database are preprocessed, encoded into tokens, and used to predict PCSS descriptors. (b) The GenMTAMP model is used to generate the sequence probability distribution, which includes an encoder, decoder, and PCSS predictor. (c) Potential AMPs are obtained sequentially based on scheduled sampling, temperature sampling, and top‐k sampling. (d) The ClaAMP model, built based on convolutional neural network (CNN), bidirectional long short‐term memory (BiLSTM), and Attention modules is used to initially screen MTAMPs. (e) The PreAMP model, combined with transfer learning (TL) based on the ClaAMP model is used to predict PCSS descriptors of the preliminarily screened AMPs. Then, the weighted score (WS) method is obtained to determine the top‐ranked MTAMPs. W1, W2, W3, W4, W5, and W6 refer to the weights of six PCSS descriptors. (f) Fmoc‐SPPS method is employed to synthesize the top‐ranked MTAMPs from WS method. The antimicrobial activity of the synthesized MTAMPs against four Gram‐negative bacteria was evaluated by minimum inhibitory concentration (MIC). The biosafety for mammalian cells was assessed through cell cytotoxicity (CC) and cell haemolysis (CH). In addition, the antimicrobial mechanisms were elucidated using the molecular dynamics simulation (MDS), circular dichroism spectroscopy (CDS), scanning electron microscopy (SEM), and confocal laser scanning microscope (CLSM).

Relevant research indicates that AMPs act on the outer membrane of Gram‐negative bacteria through three core mechanisms [4]. First, the positively charged AMPs are attracted to the negatively charged lipopolysaccharides on the surface of the outer membrane through electrostatic interactions. Then, AMPs insert into the hydrophobic region of the outer membrane. Finally, AMPs disrupt the integrity of the outer membrane. Therefore, the core characteristics of AMPs require positive charge and appropriate isoelectric point, strong hydrophobicity and balanced amphiphilicity, and stable alpha helix or beta sheet structures. Hence, the current research established a development framework for the guided design of membrane‐targeting antimicrobial peptides (MTAMPs) based on net charge, isoelectric point, hydrophobicity, amphiphilicity, alpha helix, and beta sheet. First, diverse and reliable sequence data alongside the corresponding physicochemical properties and spatial structure (PCSS) information related to the outer membrane were collected (Figure 1a). A CVAE model incorporating PCSS information (GenMTAMP) was developed to de novo design of MTAMPs, with PCSS descriptors within the desired range serving as constraints (Figure 1b). Potential MTAMPs were obtained through scheduled sampling training mode combined with temperature sampling and top‐k generation sampling methods (Figure 1c). Then, the constructed AMP classification (ClaAMP) model was utilised to determine whether potential MTAMPs were AMPs or non‐AMPs (Figure 1d). Next, the PCSS descriptors of MTAMPs classified as AMPs were predicted using the established AMP prediction (PreAMP) model, and a weighted score (WS) was applied to rank potential MTAMPs (Figure 1e). Finally, the top‐ranked MTAMPs were synthesised via Fmoc solid‐phase peptide synthesis (Fmoc‐SPPS) method. The antibacterial activity and biosafety were validated through wet experiments, and the antibacterial mechanism of the MTAMPs was elucidated (Figure 1f). To our delight, two synthesized MTAMPs called MTAMP003 and MTAMP004 demonstrated outstanding antibacterial performance against Gram‐negative bacteria while exhibiting low toxicity and haemolysis toward mammalian cells. The above results indicate that the membrane‐targeting generation strategy targeting the outer membrane of Gram‐negative bacteria proposed effectively enhances the functional targeting in de novo design of MTAMPs in the current research, providing a feasible generative design framework for the efficient and reliable development of MTAMPs with specific functional properties.

## 2 Results

### 2.1 Data Processing and Analysis

**FIGURE 2:**
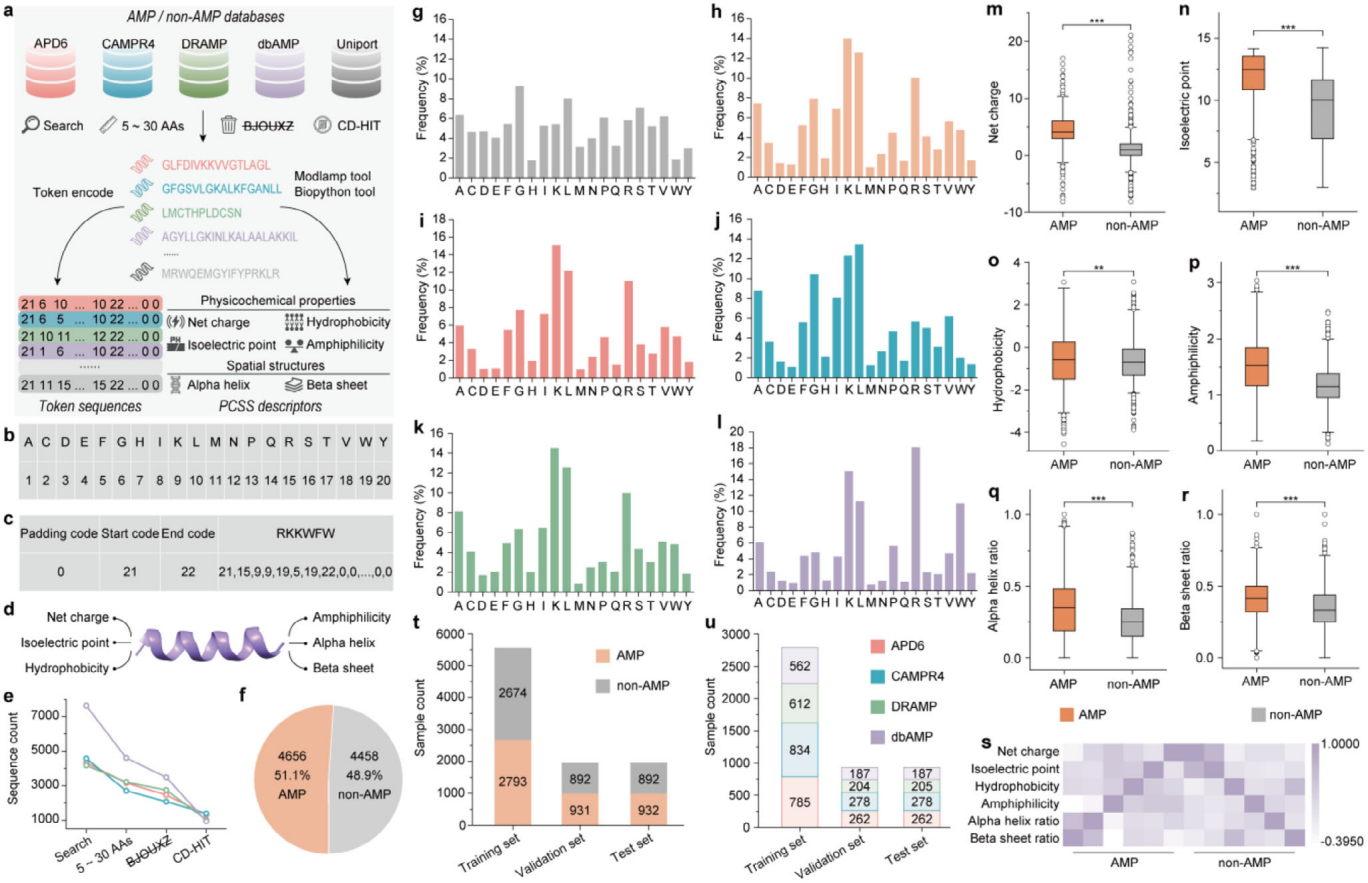
Processing and analysis results of AMPs and non‐AMPs. (a) Sources (APD6, CAMPR4, DRAMP, dbAMP, and Uniport) and preprocessing methods for AMP and non‐AMP sequence data. Preprocessing methods include: screening sequences with lengths ranging from 5 to 30 AAs, removing sequences containing non‐standard AAs, and eliminating sequences with similarity exceeding 80%. PCSS descriptors were obtained from sequence data using Modlamp and Biopython tools. (b) The rule of encoding 20 standard AAs into tokens. (c) The encoding rule of padding, start, and end codes. An example of encoding a sequence into tokens is provided. (d) The six important PCSS descriptors of AMPs referring to net charge, isoelectric point, hydrophobicity, amphiphilicity, alpha helix ratio, and beta sheet ratio. (e) The sequence count of the four AMP databases following different processing methods. (f) Data distribution for AMPs and non‐AMPs. (g, h) The AA distribution of all AMPs (g) and non‐AMPs (h). (i–l) The AA distribution of APD6 (i), CAMPR4 (j), DRAMP (k), and dbAMP (l) databases. (m–s) Significance analysis of net charge (m), isoelectric point (n), hydrophobicity (o), amphiphilicity (p), alpha helix ratio (q), beta sheet ratio (r), and correlation analysis (s) between the PCSS descriptors AMPs and non‐AMPs. The black solid line in the middle of the box represents the mean. The horizontal lines at the bottom and top of the box represent the 1/4 and 3/4 values. (t–u) AMP and non‐AMP distributions (t), and four AMP database distributions (u) across the training set, validation set, and test set.

High‐quality data acquired through data processing is a critical step for building high‐performance models, which can provide a consistent and reliable foundation for subsequent analysis (Figure 2a). During data cleaning on the obtained AMP and non‐AMP sequences, the original sequences were found to contain those shorter than 5 or longer than 30 AAs, those containing non‐standard AAs (B, J, O, U, X, Z), and those with similarity exceeding 80% (Figure 2e). Eventually, 4656 highly representative AMP sequences were selected from the four databases, and 4458 non‐AMP sequences were obtained from the Uniport database (Figure 2f), effectively avoiding class imbalance issues.

The performance differences between AMPs and non‐AMPs primarily depend on the AA types and quantities. Compared to non‐AMP sequences (Figure 2g), AMP sequences contain higher proportions of Lysine (K), Arginine (R), Leucine (L), Tryptophan (W), and Alanine (A) [15, 16] (Figure 2h). The K and R residues with positive charges can easily bind to the lipopolysaccharide molecules with negative charges from the outer membrane of Gram‐negative bacteria. The L and W residues with hydrophobicity can assist the peptide in inserting into the hydrophobic core of the lipid component of lipopolysaccharide. The A residue helps the peptide form alpha helix structures. In addition, the consistency distribution in AAs from different AMP databases indicates that the acquired AMP sequences exhibit excellent representativeness and stability (Figure 2i–l).

To quantify the differences between AMP and non‐AMP sequences, statistical analysis was performed on PCSS descriptors. Significant differences were observed in 6 PCSS descriptors, including net charge (*p* < 0.001), isoelectric point (*p* < 0.001), hydrophobicity (*p* < 0.01), amphiphilicity (*p* < 0.001), alpha helix ratio (*p* < 0.001), and beta sheet ratio (*p* < 0.001) between AMPs and non‐AMPs (Figure 2m–r). Moreover, the linear correlations among the PCSS descriptors within AMPs and non‐AMPs were relatively weak, with no excessive linear redundant information (Figure 2s). Collectively, these results highlight the fundamental differences between AMPs and non‐AMPs from the PCSS descriptor perspective.

### 2.2 ClaAMP Model Design

**FIGURE 3:**
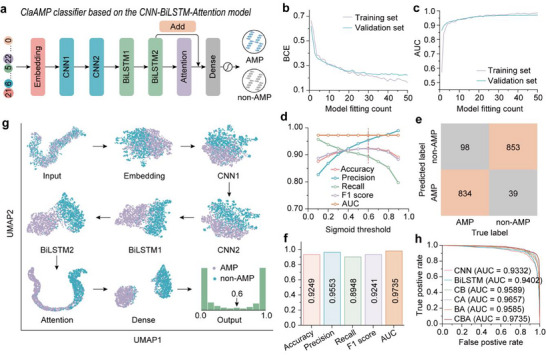
Design and evaluation of the ClaAMP model. (a) The ClaAMP model structure fusing three key modules: CNN, BiLSTM, and Attention. In addition, the ClaAMP model includes Embedding and Dense modules. (b, c) BCE loss (b) and AUC score (c) as model fitting count increases during the training stage. (d) Model performance at different sigmoid threshold in the dense output layer. Precision and recall serve as observation metrics, while accuracy and F1 score are evaluation metrics. (e) Confusion matrix of the test set when the sigmoid threshold is 0.6. (f) Accuracy, precision, recall, F1 score, and AUC of the test set. (g) Distribution visualisation of AMP and non‐AMP samples for each module of the ClaAMP model based on the UMAP method. The green frequency distribution plot shows the sigmoid value distribution of test set samples. (h) The AUC across different ablation experiments. The parameters of same modules in different models were kept consistent. The CB model includes CNN and BiLSTM modules. The CA model includes CNN and Attention modules. The BA model includes BiLSTM and Attention modules. The CBA model refers to the ClaAMP model.

The ClaAMP model can distinguish between AMPs and non‐AMPs, which consists of three important components: the CNN, BiLSTM, and Attention modules (Figure 3a). As the model fitting count increased, the binary cross entropy (BCE) loss on both the training and validation sets initially decreased rapidly before levelling off (Figure 3b), and the area under the curve (AUC) score continued to increase until stabilization (Figure 3c). By the 18th iteration, the ClaAMP model achieved nearly equal BCE and AUC values on both the training and validation sets, indicating excellent performance without overfitting. Therefore, the ClaAMP model at this stage was saved for subsequent applications, and the learning rate was 0.00001 (Figure S1a). To avoid increased wet experiment costs caused by misclassifying false positive samples, the ClaAMP model achieved both high precision and accuracy when the sigmoid function threshold in the dense output layer was set to 0.6 (Figure 3d). The confusion matrix for the test set indicated that only 4% (39/892) of non‐AMP samples were wrongly predicted as AMP samples, while 89% (834/932) of AMP samples were correctly predicted as AMP samples (Figure 3e). The accuracy and precision reached 92.49% and 95.53% respectively, further validating the reliability and effectiveness of the ClaAMP model (Figure 3f).

The results of visualizing the ClaAMP model using the uniform manifold approximation and projection (UMAP) method indicate that AMP and non‐AMP samples gradually separate into two clusters with increasing model layers (Figure 3g). In addition, the Kullback‐Leibler divergence (KLD) value continuously increases, indicating that the ClaAMP model gradually learns the intrinsic differences between AMPs and non‐AMPs (Table S1). Furthermore, ablation experiments indicate that removing any one or two of the CNN, BiLSTM, and Attention modules can decrease the ClaAMP model performance (Figure S1b–f). The AUC further indicates that all three components are indispensable (Figure 3h). The CNN module can extract local features from sequences, the BiLSTM module can capture dependency structures within the global context, while the Attention module can screen key AAs and functional regions.

To evaluate the generalisation capability of the ClaAMP model, external verification data were employed for further validation. All 60 AMPs sequences from APD6, CAMPR4, DRAMP, dbAMP, DBAASP, StarPep, and AMPSphere databases were correctly identified, and only an AMP sequence (AGFVLKGYTKTSQ) from dbAMP was misclassified as a non‐AMP. The above misclassification may reflect that the ClaAMP model tends to prioritize typical membrane‐active features, such as sufficient net positive charge and favorable amphiphilic balance, which are not prominently exhibited in this sequence. Furthermore, the ClaAMP model correctly predicted all non‐AMPs from UniPort and all AMPs reported in published articles [15, 17, 18, 19, 20, 21, 22, 23, 24, 25] (Table S2). Thus, the ClaAMP model shows robust AMP recognition capability, establishing an initial defence line for subsequently screening MTAMPs.

### 2.3 PreAMP Model Construction

**FIGURE 4:**
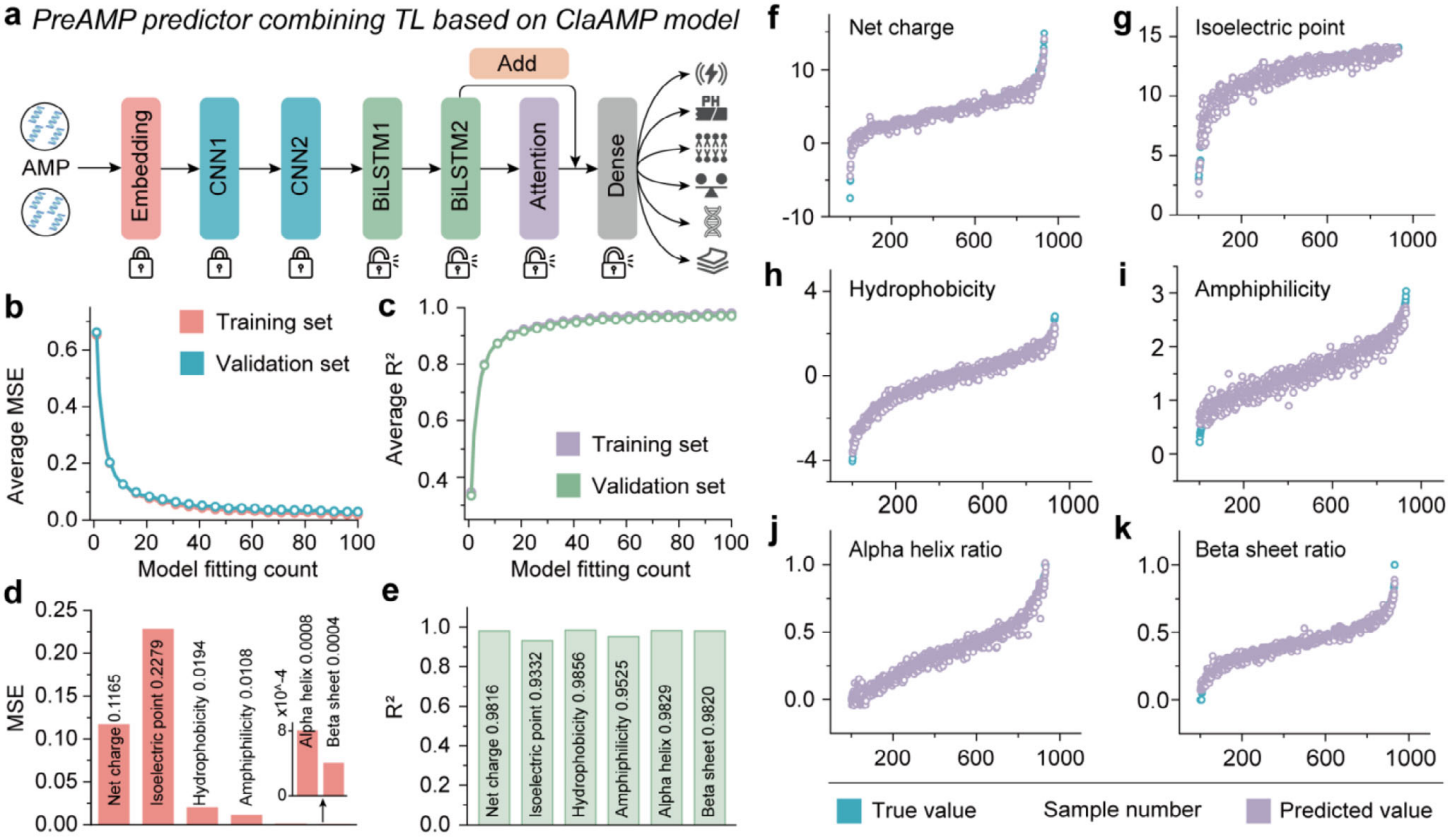
Construction and evaluation of the PreAMP model. (a) The architecture of the PreAMP model based on the ClaAMP model using the TL strategy. A closed lock indicates that this layer is frozen. An open lock indicates that this layer is not frozen. (b, c) The average MSE (b) and *R* ^2^ (c) for the training set and validation set during model training process. The average MSE and *R* ^2^ represent the real‐time averages of the six PCSS descriptors. (d, e) The MSE (d) and *R* ^2^ (e) for six PCSS descriptors based on the test set. (f–k) Overlap of net charge (f), isoelectric point (g), hydrophobicity (h), amphiphilicity (i), alpha helix ratio (j) and beta sheet ratio (k) for the test set. Each PCSS descriptor is sorted in ascending order based on true values, and the predicted values are displayed in corresponding sample positions.

The TL strategy was used to construct the PreAMP model by freezing some model layers, which maintained the same model architecture as the ClaAMP model (Figure 4a). This strategy not only reduces model training time but also enhances prediction stability, thereby mitigating overfitting risks and improving the generalisation capability. In detail, the CNN module was frozen, while keeping the BiLSTM and Attention module remained trainable. The PreAMP model preserves robust local features learned from the source task, while allowing the higher‐level sequence dependencies and attention mechanisms to adapt to the AMP features specific to the target dataset. Furthermore, significance analyses observed in net charge, isoelectric point, hydrophobicity, amphiphilicity, alpha helix ratio, and beta sheet ratio showed no significant differences (*p* < 0.0001) between the training set, validation set, and test set (Figure S2a–f). The consistency across these three datasets provides interpretable evidence for constructing a stable and robust PreAMP model.

The training process of the PreAMP model indicates that average mean squared error (MSE) initially decreases rapidly before gradually stabilising near zero (Figure 4b), while the average coefficient of determination (*R* ^2^) rises sharply before levelling off at one (Figure 4c). This learning pattern indicates that the PreAMP model efficiently captured the relationship between the main AMP sequence and PCSS descriptors early, with subsequent parameter updates further enhancing model performance. Furthermore, the detailed training processes for each PCSS descriptor indicate that the PreAMP model converges and optimizes stably, with the capability to accurately predict all PCSS descriptors simultaneously (Figure S2g–l). These results confirm that the PreAMP model maintains robust learning dynamics and achieves reliable predictive performance across PCSS descriptors.

Predicted results on the test set show that the MSE for all PCSS descriptors is within 1.00% of the true values (Figure 4d), with R^2^ exceeding 93.00% for each (Figure 4e), indicating that the PreAMP model can accurately predict PCSS descriptors with high performance. In addition, the high overlap between predicted and true values visually confirms that the PreAMP model successfully captures key feature patterns of PCSS descriptors (Figure 4f–k). Furthermore, the error between true and predicted values reveals that most samples exhibit low prediction errors, with residuals concentrated around zero and symmetrically distributed (Figure S2m–r). These results indicate that the PreAMP model does not suffer from overfitting or systematic errors.

To further evaluate the generalization capability, the PreAMP model was applied to predict PCSS descriptors for external verification data. Comparing prediction results of the PreAMP model with those from Modlamp and Biopython tools reveals that the mean and standard deviation (SD) are closely aligned, with only PreAMP model exhibiting slightly broader maximum and minimum (Table S3). Additionally, detailed PCSS descriptor prediction results of the external verification data based on Modlamp and Biopython tools and PreAMP model can be found in Tables S4 and S5, respectively. The above results indicate that PreAMP model not only accurately reproduces the overall distribution characteristics of external verification data, but also possesses stronger numerical coverage capabilities, capturing more extreme value ranges within the data.

### 2.4 GenMTAMP Model Development

**FIGURE 5:**
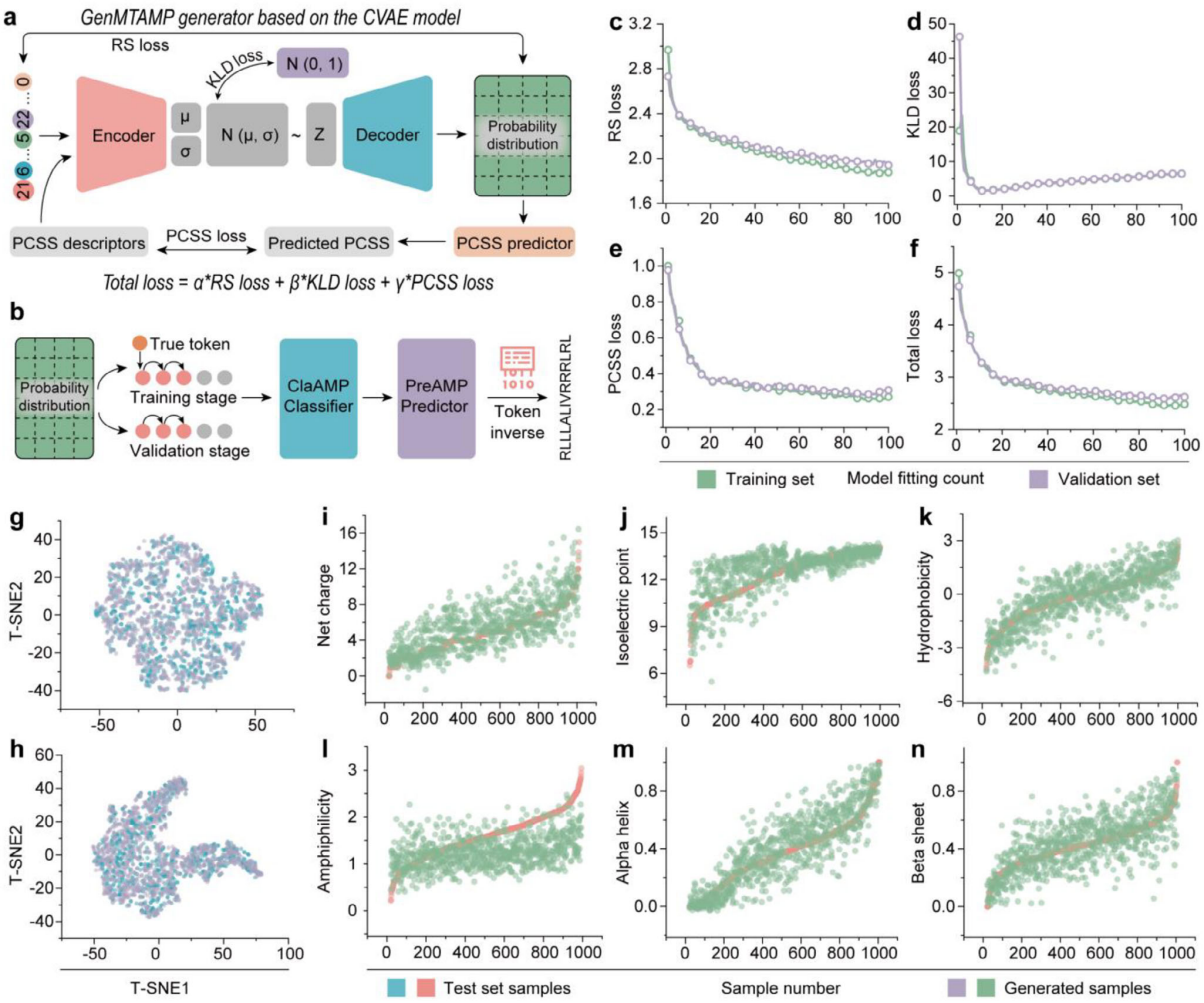
Development and evaluation of the GenMTAMP model. (a) The framework of the GenMTAMP model based on the CVAE model architecture. The GenMTAMP model includes an encoder, a decoder, and a PCSS predictor. The purple rectangle represents the standard normal distribution. The α, β, and γ denote the weights for RS loss, KLD loss, and PCSS loss, respectively. (b) Training and validation principles and application workflow of the GenMTAMP generator. During the training stage, the GenMTAMP generator with progressively decreasing teacher sampling ratios. For the validation stage, the GenMTAMP generator adopts a sampling approach combining temperature and top‐k. For stepwise autoregression, the pink circle represents the token obtained from the previous prediction step, while the gray circle represents the token being predicted. (c–f) RS loss (c), KLD loss (d), PCSS loss (e), and total loss (f) of the training and validation sets during the GenMTAMP model training process. (g, h) The mean (g) and SD (h) distributions of test set and generated samples in the latent space based on the t‐SNE method. (i–n) Net charge (i), isoelectric point (j), hydrophobicity (k), amphiphilicity (l), alpha helix ratio (m), and beta sheet ratio (n) of the test set and generated samples. Each PCSS descriptor is sorted in ascending order within the test set, and the generated value of the corresponding sample is displayed in the exact same position.

**FIGURE 6:**
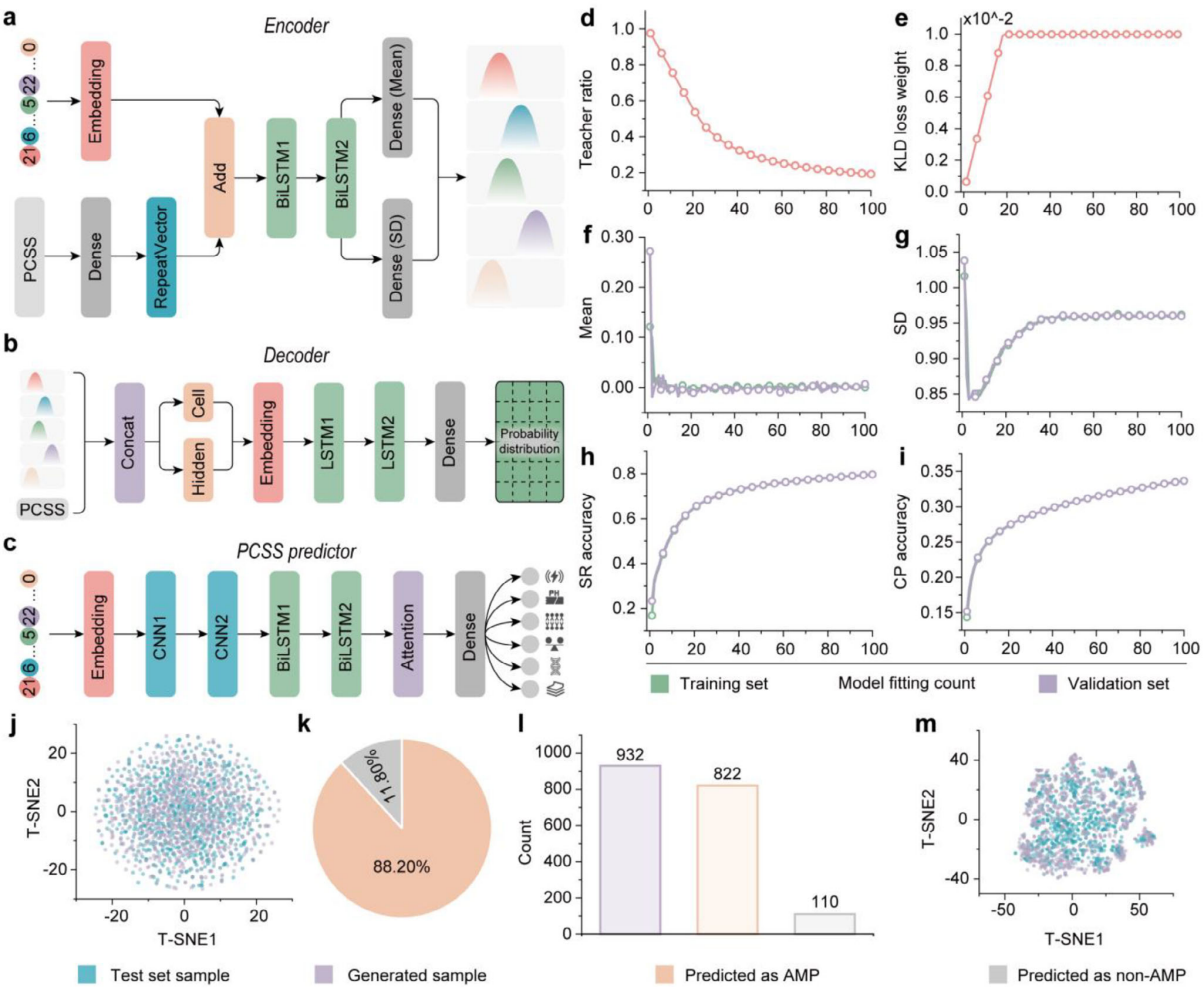
Composition, training process, and evaluation results of the GenMTAMP model. (a–c) Model architecture of the encoder (a), the decoder (b), and the PCSS predictor (c). Different normal distribution diagrams represent distinct variables in the latent space. (d–i) Teacher ratio (d), KLD loss weight (e), mean (f), SD (g), SR accuracy (h), and CP accuracy (i) of training and validation sets during the training process. (j) The latent vector distribution of the test set and generated samples. (k, l) Prediction rates (k) and predicted counts (l) of generated samples based on the ClaAMP model. (m) The AA frequency distribution of test set and generated samples.

The VAE model can output the mean and SD of latent variables through KLD regularization, forming a continuous and structured probability distribution in the latent space [14]. The above mechanism can assist the VAE model to perform smooth interpolation and plausible sampling in the latent space, thereby ensuring generated peptides maintain biological plausibility. To enhance the targeted generation capability of the VAE model for designing MTAMPs, the CVAE model named GenMTAMP was constructed by incorporating PCSS descriptors associated with Gram‐negative bacterial outer membranes as constraints in the current research (Figure 5a). The GenMTAMP model is composed of three modules: an encoder (Figure 6a), a decoder (Figure 6b), and a PCSS predictor (Figure 6c). The encoder can encode AMP sequences and PCSS descriptors into latent space information, while the decoder can decode latent space information and PCSS descriptors into the probabilistic distribution. The PCSS predictor can estimate the PCSS features of generated sequences and provide feedback, thereby enhancing conditional consistency and generation reliability. These three components work synergistically to ensure the overall performance of the GenMTAMP model in latent representation learning, conditional constraint consistency, and directed generation reliability.

For both training and validation stages, the encoder operates consistently by compiling AMP sequences and PCSS descriptors into the latent space. Each latent information is represented through the mean and SD. The mean captures the primary semantic information of latent features, while the SD encodes the randomness of latent variables, supporting subsequent reparameterized sampling and diverse generation. The decoder generates sequences using a stepwise autoregressive mode, starting from the start token and ending with the end token. During the training stage, the planned sampling strategy replaces full teacher forcing. For each decoding step, input tokens are randomly selected from the ground truth sequence or previously generated output of the decoder according to a teacher ratio. As the model fitting count increases, the teacher ratio gradually decreases (Figure 6d). This sampling mechanism maintains training stability while effectively mitigating exposure bias. During the validation stage, no truth tokens are provided. Therefore, the decoder performs fully autoregressive inference, where each input token is determined solely based on the predicted token at the preceding step. Furthermore, the constructed ClaAMP classifier and PreAMP predictor were employed for dual filtering, with MTAMPs obtained through token inverse (Figure 5b). This workflow ensures that the generative performance of the GenMTAMP model is assessed under conditions that reflect practical application scenarios, thereby comprehensively assessing MTAMP quality.

To enable the GenMTAMP model to reconstruct sequences and learn to generate target sequences, the KLD loss weight was initially set close to zero and then gradually increased (Figure 6e). As the model fitting count increased, the reconstruction (RS) loss first decreased rapidly and then slowed down (Figure 5c). The results indicate that the GenMTAMP model can rapidly learn to reconstruct sequences, which is crucial for capturing key patterns and features within sequences and provides a foundation for generating high‐quality sequences. Meanwhile, the KLD loss rapidly decreases from a high initial value and stabilizes at a low level during the early training stage, owing to the low initial KLD weights and the high model dependence on truth tokens. As KL annealing progresses and the teacher ratio gradually decreases, the encoder increasingly utilizes the latent space to express sequence features, causing the KLD loss to rise slightly (Figure 5d). This facilitates the structurization and regularization of the latent space. The continuity and structure of the latent space enable the GenMTAMP model to perform smooth sampling during generation, resulting in diverse and expected sequences. At this stage, the PCSS loss between the predicted PCSS descriptors from the PCSS predictor and the actual PCSS descriptors continues to decrease, further validating the effective model optimization process (Figure 5e). In detail, according to the importance of PCSS descriptors, the weights for net charge, isoelectric point, hydrophobicity, amphiphilicity, alpha helix ratio, and beta sheet ratio are set to 0.2, 0.1, 0.2, 0.2, 0.2, and 0.1, respectively. Finally, the total loss function constructed through linear weighting of the three losses becomes the overall optimization objective, which continuously decreases as the model fitting count increases (Figure 5f). Specifically, RS loss and PCSS loss were set to 1.0 and 2.0, respectively. This training strategy ensures rapid model convergence while effectively preventing overfitting and latent space collapse. The above results demonstrate that the GenMTAMP model has been successfully constructed, effectively balancing sequence reconstruction capability, generative diversity, and target sequence alignment capability.

For the latent space, the mean closer to 0 indicates that a higher degree of alignment between the posterior distribution and the standard normal distribution, while the SD closer to 1 signifies that the latent representation better satisfies prior constraints. During the training process, the mean rapidly converged toward 0 (Figure 6f), while the SD initially decreased to approximately 0.8 before gradually increasing (Figure 6g). This phenomenon indicates mild posterior shrinkage during early training, where the decoder primarily relies on the autoregressive capabilities for reconstruction while weakly utilizing latent variables, leading to compression of the posterior distribution. As the KLD weight gradually increases, the GenMTAMP model begins to fully leverage the latent space to express sequence structure, causing the posterior distribution to progressively approach the standard normal prior. These findings reflect that the latent spatial structure transforms from an amorphous state into a distinguishable, continuous, and stable state. Moreover, both sequence reconstruction (SR) accuracy (Figure 6h) and condition prediction (CP) accuracy (Figure 6i) steadily increased throughout training process, indicating that the GenMTAMP model progressively mastered sequence reconstruction capabilities while simultaneously enhancing compliance with the target PCSS descriptors. The results demonstrate the synchronous reinforcement of generation quality and conditional consistency.

During the model evaluation stage using the test set, sequences generated with different temperature sampling parameters (0.8, 1.0, 1.2) and top‐k sampling parameters (1, 3, 5) were evaluated based on the ClaAMP classifier and PreAMP predictor. Results indicate that when temperature parameters are same, the ability to accurately predict AMPs slightly decreases as top‐k increases. However, the PCSS descriptors of the generated sequences more closely approximate true values. Specifically, when the temperature is 1.0 and top‐k is 5, the six PCSS descriptors showed the minimum MSE (Table S6). Therefore, the sampling strategy with a temperature of 1.0 and top‐k of 5 is used for subsequent MTAMP generation.

Generated sequences exhibit high overlap with test sequences in both the latent space and the mean (Figure 5g) and SD (Figure 5h) based on the t‐distributed stochastic neighbour embedding (t‐SNE). The results indicate that the GenMTAMP model consistently captures core distributional features of sequences at the latent representation level. Furthermore, the latent vector (*z*) of generated sequences exhibits high consistency with test sequences (Figure 6j). The phenomenon demonstrates that the GenMTAMP model not only reproduces the latent structural patterns of true sequences but also maintains continuity and distinguishability in the latent space. This lays a reliable foundation for subsequent diverse generation and conditional control.

The generated sequences were input into the ClaAMP model for initial screening. According to the ClaAMP prediction results, 88.20% of the generated sequences were predicted as AMPs, while 11.80% were predicted as non‐AMPs (Figure 6k). In terms of absolute numbers, 822 generated sequences were predicted as AMPs and 110 were predicted as non‐AMPs (Figure 6l). These results indicate that a high proportion of the generated sequences were recognized by the ClaAMP model as potential candidates with AMP characteristics, suggesting that the GenMTAMP model effectively captured important sequence features associated with AMPs. The t‐SNE visualization of the AA frequency shows that generated sequences highly overlap with test sequences in the low‐dimensional space (Figure 6m), demonstrating that the GenMTAMP model can learn and maintain similarity to true AMPs at the sequence composition feature level.

Furthermore, the filtered sequences were input into the PreAMP predictor, revealing that the generated sequences highly overlapped with true peptides in PCSS distributions (Figure 5i–n). Although the overlap between the amphiphilicity of the generated sequences and that of the actual sequences is slightly lower, the primary reason lies in the fact that amphiphilicity depends on residue arrangement and spatial periodicity, whereas the conditional constraints of the GenMTAMP model are primarily based on global PCSS descripts. Overall, the generated sequences still exhibit correct functional tendencies and maintain reasonable overall physicochemical properties, indicating that the GenMTAMP model possesses both sequence reconstruction capability and innovative potential.

A comparative analysis of five different generative models showed that sequences generated by the GenMTAMP model exhibited the highest predictive rate by the ClaAMP classifier as antimicrobial peptides, reaching 99.30% (Table S7). For AMPs screened by the ClaAMP model, the average PCSS descriptors predicted by the PreAMP model indicate that AMPs generated by the GenMTAMP model exhibited the highest average values for net charge, isoelectric point, hydrophobicity, amphiphilicity, and alpha helix, while exhibiting the lowest average value for beta sheet (Table S8). These results indicate that the GenMTAMP model is more effective at generating AMP candidates with expected physicochemical properties and spatial structures.

### 2.5 MTAMP Model Generation

**FIGURE 7:**
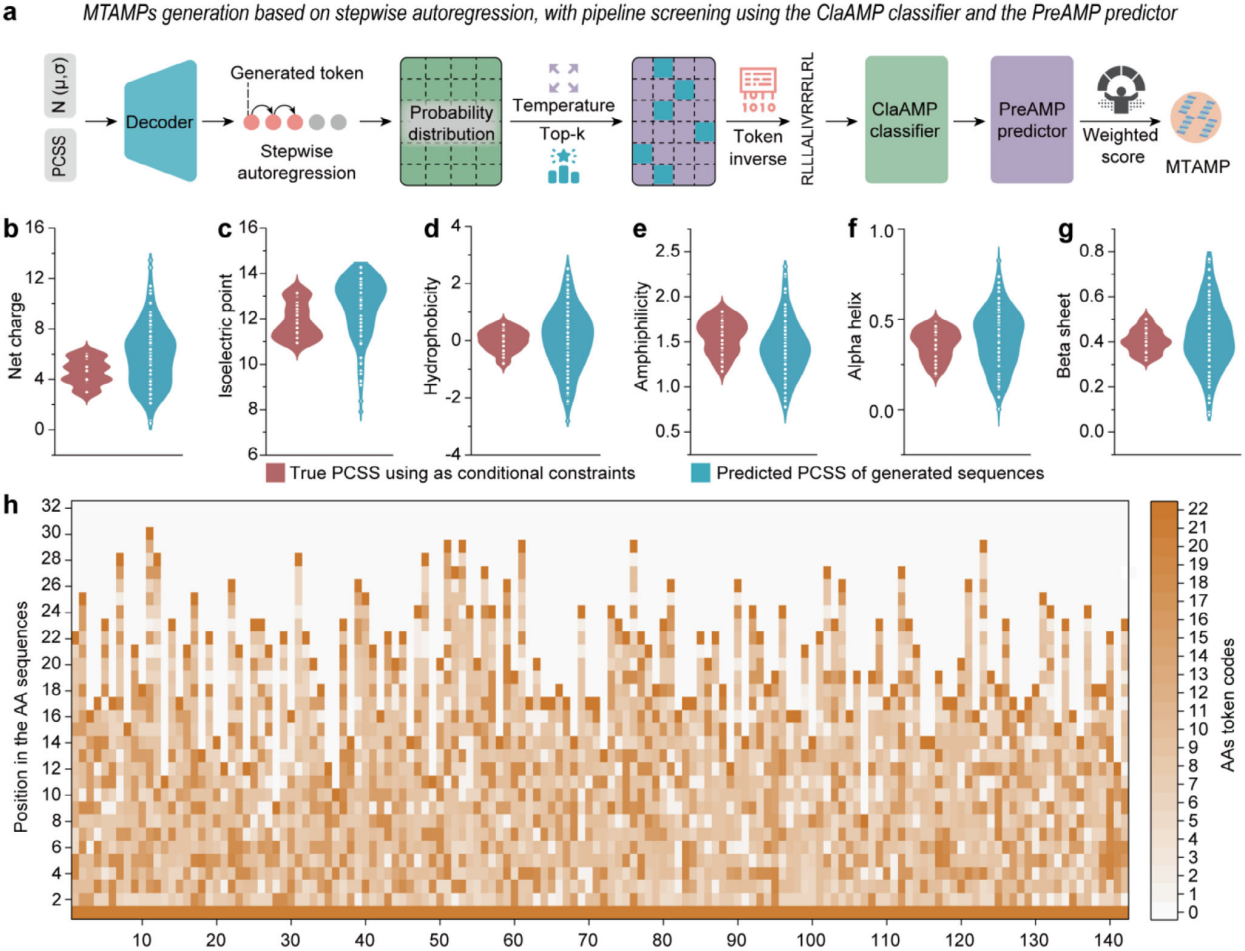
Sequences generated by the GenMTAMP model combining the ClaAMP classifier and PreAMP predictor. (a) The detailed steps for screening MTAMPs using the GenMTAMP model. MTAMPs generation based on stepwise autoregression, with pipeline screening using the ClaAMP classifier and the PreAMP predictor. For stepwise autoregression, the pink circle represents the token obtained from the previous prediction step, while the gray circle represents the token being predicted. (b–g) Net charge (b), isoelectric point (c), hydrophobicity (d), amphiphilicity (e), alpha helix ratio (f), beta sheet ratio (g) as constraint conditions, and generated sequences. (h) AA sequence structure heatmap of the 142 screened MTAMPs. Darker colors indicate larger tokens.

The training data were derived from general AMP‐related databases rather than being limited to peptides specific to Gram‐negative bacteria. During the peptide generation stage, the model framework was pre‐trained using selected PCSS descriptor values and subsequently screened to achieve specificity for Gram‐negative bacteria. To ensure the GenMTAMP model learns more stable and reasonable conditional distributions within typical PCSS ranges, 143 AMPs were selected from 4656 AMPs, with all PCSS descriptors falling within the 1/4 to 3/4 range (Table S9). By using the PCSS descriptors of these 143 AMPs as conditional constraints, both the interference from extreme samples is mitigated, and the biological plausibility of generated sequences is enhanced. Then, the decoder decodes the latent variables sampled from standard normal space and the above 143 PCSS descriptors into probability distributions. The AA sequences are generated from the above probability distribution using a sampling strategy with a temperature of 1.0 and top‐k of 5. This sampling strategy ensures sequence diversity while maintaining stable and reliable PCSS properties and functional predictions for the generated sequences. Then, generated tokens are converted into AA sequences using a token inverse approach. The ClaAMP classifier identified 142 sequences as AMPs, with one sequence (GSPSPSGGGSSRKGRSRGIARAGPGSGP) classified as non‐AMP. Furthermore, the PCSS descriptors predicted by the PreAMP predictor showed good agreement with actual PCSS descriptors (Table S10). The PCSS descriptors of generated sequences contained true PCSS descriptors, indicating that the GenMTAMP model effectively inherited and preserved the physicochemical feature distribution of real AMPs during generation, thereby avoiding physically unreasonable or biologically infeasible sequences (Figure 7b–g). In addition, the GenMTAMP model can generate sequences of variable lengths, with most concentrated between 10 and 20 AAs (Figure 7h). This length distribution not only aligns closely with the typical scale of natural AMPs but also offers significant advantages in balancing antimicrobial activity, synthetic feasibility, and biosafety. Finally, the weight score (WS) method was used to score the 142 generated sequences, 8 sequences with a score of 1.0 (Grade 1, 5.6%), 81 sequences scored above 0.9 (Grade 2, 57.04%), 41 sequences scored between 0.8 and 0.9 (Grade 2, 28.87%), 12 sequences scored below (Grade 4, 8.45%) (Table S11). The WS evaluation results indicate that the PCSS descriptors of the vast majority of generated sequences exhibit good consistency with ideal PCSS features, further validating the rationality and potential functionality of the GenMTAMP model. To further validate the practical performance of the generated sequences, the top 8 sequences with the highest WS were selected from the 142 generated sequences for actual synthesis and experimental verification.

### 2.6 Antimicrobial Activity and Biosafety

**FIGURE 8:**
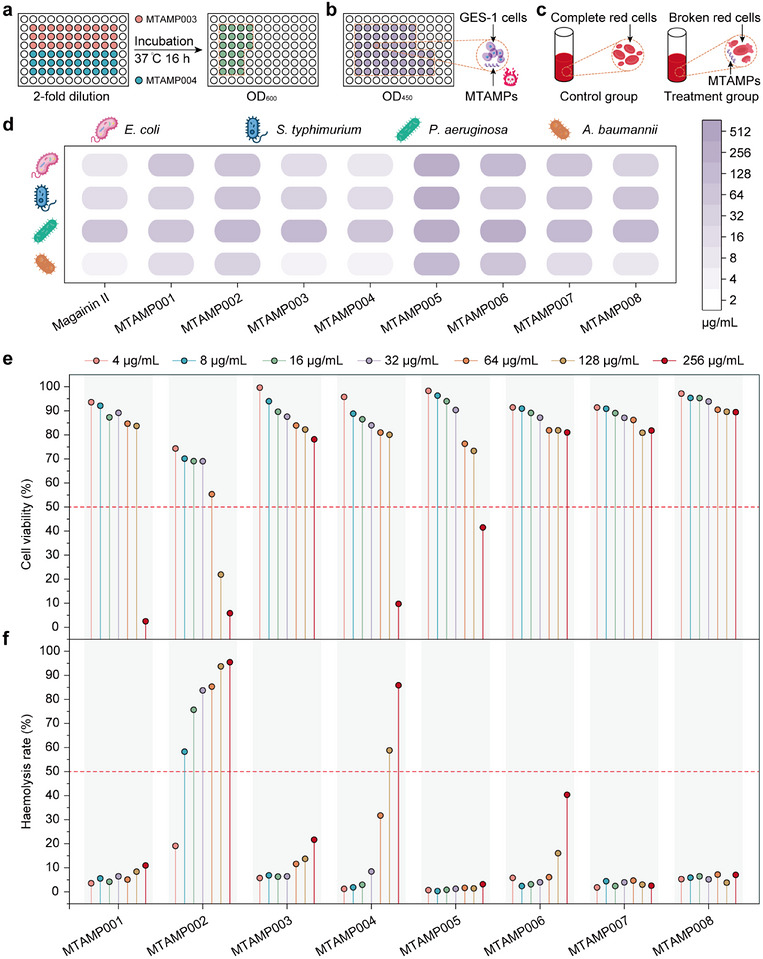
Antimicrobial activity and biological safety of MTAMPs. (a–c) Simple detection scheme for MIC (a), cytotoxicity (b), and haemolysis (c) of the MTAMPs. Red and blue wells represent two MTAMPs, respectively. Green wells indicate bacterial cultures with turbidity after MTAMP treatment, which exhibit high OD_600_ values. Purple wells indicate cell cultures with turbidity after MTAMP treatment, which exhibit high OD_450_ values. (d) MIC values of MTAMPs against four Gram‐negative bacteria referring to *E. coli* ATCC 25922, *P. aeruginosa* ATCC 27853, *S. typhimurium* CMCC (B) 50115, and *A. baumannii* ATCC 19606. Lighter purple indicates lower MIC values. (e) Toxicity of MTAMPs to the GES‐1 cells. (f) The haemolysis of MTAMPs on the rabbit red cells. The red dashed line represents the 50% cell viability and haemolysis rate.

To validate the antimicrobial activity and biosafety of the top eight MTAMPs, minimum inhibitory concentration (MIC) (Figure 8a), cell cytotoxicity (CC) (Figure 8b), and cell haemolysis (CH) (Figure 8c) were used to assess their effects on four Gram‐negative bacteria (*E. coli* ATCC 25922, *P. aeruginosa* ATCC 27853, *S. typhimurium* CMCC (B) 50115, and *A. baumannii*) (Magainin II serving as the positive control), the GES‐1 cells, and rabbit red cells. The MIC results indicate that all eight MTAMPs exhibit antibacterial activity, except for MTAMP005 (Figure 8d). Both MTAMP003 and MTAMP004 exhibited MIC values as low as 4 µg/mL against *A. baumannii*. In addition, they show promising antibacterial effects against *E. coli* and *S. typhimurium*. Unfortunately, some AMPs exhibited high MIC values of 256 µg/mL against *P. aeruginosa*. This may be attributed to the highly developed outer membrane barrier, active efflux pump systems, and biofilm formation capabilities, which limit peptide penetration and antibacterial activity [26, 27]. Nevertheless, comparative results with Magainin II indicate that the designed AMPs, particularly MTAMP003 and MTAMP004, exhibit encouraging antimicrobial potential against a broad spectrum of Gram‐negative bacteria. Toxicity assessment of different types and concentrations of MTAMPs against GES‐1 cells revealed that all eight MTAMPs maintained GES‐1 cell viability above 50% at concentrations below 128 µg/mL, except for MTAMP002 (Figure 8e). Furthermore, haemolysis assays on rabbit red cells indicated that most peptides exhibited haemolysis rates below 50%, except for MTAMP002 and MTAMP004 (Figure 8f).

MTAMP003 and MTAMP004 show excellent antibacterial activity, low toxicity, and low haemolysis, representing relatively superior MTAMPs. Conversely, MTAMP005 exhibits poor antibacterial activity, while MTAMP002 displays high toxicity and haemolysis, representing relatively inferior MTAMPs. Since MTAMP005 (VVGVGIIAGVVKGGG) lacked sufficient positive charge and a typical amphiphilic structure, the peptide struggled to effectively adsorb and disrupt bacterial cell membranes, resulting in weak antibacterial activity. For MTAMP002 (RLILVPIVALLVIRRLVKKRKRR), both excessive hydrophobicity and high positive charge density result in a lack of selectivity toward mammalian cell membranes. The peptide strongly disrupts GES‐1 cell membranes and induces haemolysis in rabbit red cells, exhibiting high cytotoxicity and haemolysis. Additionally, MTAMP001, MTAMP006, MTAMP007, and MTAMP008 showed good antibacterial effects with low toxicity and haemolysis, thereby qualifying as relatively successful MTAMPs. The above results indicate that the antibacterial activity and biosafety of MTAMPs are jointly determined by a balanced combination of cationic charge, hydrophobicity, and amphipathic structure. Furthermore, as the MTAMP concentration increases, the cytotoxicity (Figure S3a–h) and haemolysis (Figure S3i–p) toward mammals gradually rise. This phenomenon indicates a significant concentration‐dependent feature in the membrane action mechanism of MTAMPs, with their membrane selectivity diminishing at high concentrations. Overall, these experimental validation results further demonstrate that the current research framework offers reliable and practical guidance for the targeted design and screening of MTAMPs.

To further evaluate whether the designed peptides were specific to Gram‐negative bacteria, an additional assessment was conducted on MTAMP003, MTAMP004, and Magainin II against two Gram‐positive bacterial (*S. aureus* ATCC 25923 and *B. subtilis* ATCC 9372) and one fungus (*C. albicans* ATCC 10231). The results showed that MTAMP003 and MTAMP004 maintained strong activity against both Gram‐positive bacteria, with MIC values as low as 4 µg/mL, indicating that these MTAMPs were not restricted to Gram‐negative bacteria. In contrast, their activities against *C. albicans* were much weaker, suggesting limited antifungal efficacy (Table S12). This difference may be attributed to the distinct envelope and membrane properties of fungi compared with bacteria. Gram‐positive bacteria possess negatively charged cell envelopes that still favour electrostatic interactions with MTAMPs. However, the more complex cell wall and membrane composition of fungus may reduce the susceptibility of *C. albicans* to these MTAMPs.

### 2.7 Antimicrobial Mechanism

**FIGURE 9:**
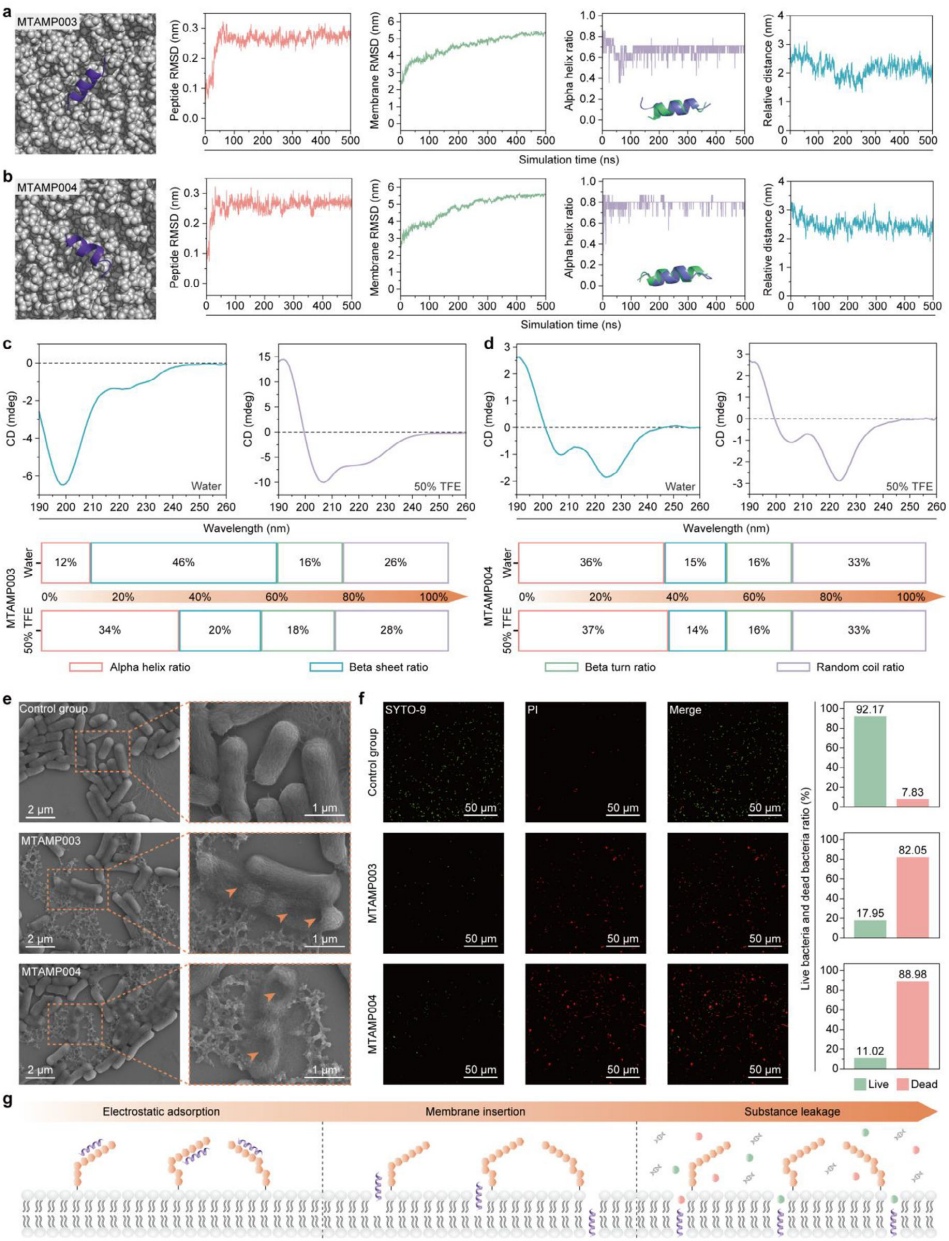
Antimicrobial mechanisms of MTAMPs. (a, b) Schematic diagrams of MDS, peptide RMSD, membrane RMSD, alpha helix ratio, and relative distance between peptide and membrane mass centre of the MTAMP003 (a) and MTAMP004 (b). Purple peptides represent the peptides at 0 ns, while green peptides represent those at 500 ns. (c, d) CDS and secondary structure ratio of MTAMP003 (c) and MTAMP004 (d) in water and 50% TFE. The black dashed line indicates the zero level. The orange arrows represent the sum of different secondary structure ratios. (e, f) SEM (e) and CLSM (f) images of control group, MTAMP003 and MTAMP004. Orange arrows indicate *E. coli* damage regions. Green cells represent live *E. coli*, while red cells indicate dead *E. coli*. (g) Mechanism of MTAMP action on the bacterial outer membrane. Schematic illustrating how MTAMP disrupts bacterial outer membrane structure and induces cell death via electrostatic adsorption and amphipathic alpha helix membrane insertion. The purple spiral represents MTAMPs, the orange circle represents lipopolysaccharides, the red circle represents sodium ions, and the green circle represents potassium ions.

Elucidating the antibacterial mechanism of MTAMP is essential, thus MTAMP003 and MTAMP004 were selected as research subjects. Full‐atom MDS revealed the states of MTAMP003 (Figure 9a) and MTAMP004 (Figure 9b) on the phospholipid bilayer surface during the simulation process. As the simulation time increased, both the peptides and the membrane reached stable root mean square deviation (RMSD) values, indicating reliable simulation results. Further alpha helix ratio analysis revealed that both MTAMP003 and MTAMP004 consistently maintained a high proportion of 0.7, indicating highly stable alpha helix structures for both peptides. In addition, comparison of the secondary structure diagrams for the two peptides at the simulation start (0 ns, green) and endpoint (500 ns, purple) showed high structural overlap. As simulation time increases, the relative distance between the centre of the peptide and the centre of the membrane gradually decreases, indicating continuous peptide‐membrane approach and interaction. Furthermore, MDS results for the remaining six MTAMPs are presented in Figure S4a–f. As shown in Figure S4c, MTAMP005 exhibits structural instability and undergoes helical unwinding, which explains its relatively poor antibacterial activity. To further validate the MDS results, a positive control (Magainin II) and a negative control (AGFVLKGYTKTSQ) were additionally included in the simulations. The positive control exhibited performance generally similar to MTAMP003 and MTAMP004 (Figure S4g). In contrast, the negative control displayed a relatively large initial RMSD and maintained a non‐helical structure throughout the simulation, indicating poor structural stability (Figure S4h). These control results further support the reliability and rationality of the MDS analysis.

Generally, the antimicrobial efficacy of AMPs is closely related to the secondary structure. Related studies indicate that AMPs exhibit a lower helix state in water, while the outer membrane of Gram‐negative bacteria induces an increase in their helix state [28]. Circular dichroism spectrum (CDS) results indicate that MTAMP003 exhibits only 12% alpha helix content in water, whereas this proportion increases to 34% when present in 50% TFE (Figure 9c). However, MTAMP004 maintains a relatively high alpha helix ratio even in water, resulting in only a 1% increase in the alpha helix ratio in 50% TFE (Figure 9d). Additionally, CDS results for the remaining six MTAMP variants are presented in Figure S5a–f. As shown in Figure S5b, MTAMP002 exhibits a higher alpha helix ratio in 50% TFE, making it more likely to cause damage to mammalian cells and resulting in higher toxicity and haemolysis. The above findings effectively explain the previous observation that the MTAMP004 with higher concentrations exhibits higher toxicity and haemolysis compared to the MTAMP003.

To investigate the mechanism of action of MTAMP003 and MTAMP004 against Gram‐negative bacteria, *E. coli* was selected as the test organism. Scanning electron microscopy (SEM) revealed that bacteria in the control group appeared fully intact, whereas those treated with MTAMP003 and MTAMP004 exhibited distinct concavities (Figure 9e). Furthermore, confocal laser scanning microscope (CLSM) results indicated that most control bacteria remained viable (shown in green), while bacteria treated with MTAMP003 and MTAMP004 predominantly exhibited progressive cell death (shown in red) (Figure 9f). Counting of viable and dead bacteria indicated that the dead bacteria ratio in the treatment groups was significantly higher than those in the control group.

Integrated MDS and multiscale experimental characterization reveal that the antibacterial mechanism of MTAMPs likely follows a typical membrane‐targeting pathway. First, the peptide enriches and anchors to the bacterial cell membrane surface via electrostatic interactions, maintaining a stable amphiphilic alpha helix conformation induced by the membrane environment. Then, the peptide inserts directionally into the lipid bilayer, disrupting the ordered arrangement of the membrane and reducing the structural stability. Finally, membrane permeability increases, intracellular substances leak out, and cellular homeostasis is disrupted, leading to bacterial death (Figure 9g).

## 3 Discussion and Conclusions

The AMR has become a major threat to global public health, with Gram‐negative bacteria posing particular challenges due to their outer membrane barrier. The AMPs, which can target and disrupt bacterial outer membranes, are considered a promising alternative strategy to address Gram‐negative bacterial AMR. Therefore, the current research proposes a generative AI‐based AMP design framework based on CVAE model. The framework can guide MTAMP generation by focusing on the Gram‐negative bacteria outer membrane. Wet experiments confirmed that the candidate AMPs exhibit excellent antibacterial activity and biosafety, and elucidated their antibacterial mechanisms. Therefore, the current research demonstrates the potential application of a generative AI framework in the rational design of AMPs targeting specific bacterial barrier structures.

Several models [16, 29, 30, 31, 32] have been developed to screen AMPs from existing natural sequences, database‐based screening strategies are inherently constrained by the known natural sequence space, making it difficult to overcome limitations imposed by existing sequence sources. Although some generative models [7, 10, 12, 33, 34, 35] have been proposed for de novo design of AMPs, they still face limitations in targeted design control. Therefore, the current research integrates key biophysical features (e.g., negatively charged lipopolysaccharide structures and phospholipid bilayers with hydrophilic‐hydrophobic balance) of the Gram‐negative bacterial outer membrane with typical structural characteristics (e.g., positively charged AA composition and amphipathic alpha helical conformation) of AMPs to establish a rational design and screening workflow for MTAMPs.

Through reflection and analysis, our study still presents several limitations. The GenMTAMP framework primarily learns knowledge from linear peptide sequences composed of standard AAs, lacking non‐natural AAs or complex topological structures. These limitations constrain the exploration of the broader AMP chemical space. Therefore, integrating three‐dimensional structural information from AlphaFold3 [36] and incorporating non‐standard AAs may further enhance the performance of the GenMTAMP framework. Furthermore, the GenMTAMP framework, as a data‐driven generative model, exhibits a lack of interpretability in the internal decision‐making process. The molecular mechanisms underlying the interaction between AMPs and the outer membrane of Gram‐negative bacteria remain difficult to elucidate in depth from the feature dimension. In addition, protein language models may provide more powerful sequence representations and further improve prediction accuracy. Their integration into AMP generation and screening frameworks represents a promising direction for future research. Finally, the scale of experimental validation remains relatively limited. Future work should further assess the robustness and translational potential of the model across larger sets of candidate peptides and diverse biological models.

Overall, the current research systematically validated the feasibility and effectiveness of generative AI in AMP rational design by leveraging the structural characteristics of Gram‐negative bacterial outer membranes. Wet experiment results demonstrate that the designed MTAMP003 and MTAMP004 exhibit outstanding antibacterial activity against Gram‐negative bacteria while maintaining low cell cytotoxicity and haemolysis toward mammalian cells. Overall, the current research proposes that the multi‐constraint generation strategy can efficiently and reliably develop MTAMPs, providing a universal and scalable computational design paradigm.

## 4 Materials and Methods

### 4.1 AMP Sequences

The AMP sequences were downloaded from APD6 (https://aps.unmc.edu/AP/), CAMPR4 (https://camp.bicnirrh.res.in/index.php), DRAMP (http://dramp.cpu‐bioinfor.org/), and dbAMP (https://awi.cuhk.edu.cn/dbAMP/) before July 2025. All sequences are annotated as experimentally validated. The four databases are open access and contain AMP sequences from different sources.

### 4.2 Non‐AMP Sequences

The non‐AMP sequences were obtained from Uniport (https://www.uniprot.org/) before July 2025. All sequences containing keywords such as antimicrobial, antibacterial, antifungal, antiviral, antibiotic, antimalarial, antiparasitic, anticancer, defense, defensin, cathelicidin, histatin, bacteriocin, fungicide, toxin, effector, and excreted were excluded.

### 4.3 External Verification Data

External verification data includes AMPs acquired from APD6, CAMPR4, DRAMP, dbAMP, DBAASP (https://www.dbaasp.org/home), StarPep (https://starpepweb.org/), AMPSphere (https://ampsphere.big‐data‐biology.org/home), 10 published articles [15, 17, 18, 19, 20, 21, 22, 23, 24, 25], and non‐AMPs acquired from Uniport. 10 records were randomly selected from each AMP database, and 20 records were randomly selected from the non‐AMP database.

### 4.4 Sequence Cleaning

The length of AMP and non‐AMP sequences was limited to between 5 and 30 AAs. AMP and non‐AMP sequences containing non‐standard AAs (B, J, O, U, X, Z) were excluded. Duplicate sequences with similarity exceeding 80% were removed using the cluster database at high identity with tolerance (CD‐HIT) method. When an AMP sequence and a non‐AMP sequence were assigned to the same CD‐HIT cluster, the non‐AMP sequence was preferentially removed.

### 4.5 Sequence Encoding

The 20 standard AAs (A, C, D, E, F, G, H, I, K, L, M, N, P, Q, R, S, T, V, W, Y) were encoded as 20 positive integers (1, 2, 3, 4, 5, 6, 7, 8, 9, 10, 11, 12, 13, 14, 15, 16,1 17, 18, 19, 20) (Figure 2b). Sequences shorter than 30 AAs were padded with 0 to reach length 30. In addition, the start code is 21, and the end code is 22. For example, the encoded token of RKKWFW is 21, 15, 9, 9, 19, 5, 19, 22, 0, 0, …, 0, 0 (Figure 2c), with a total length of 32.

### 4.6 PCSS Descriptors

The Modlamp (https://modlamp.org/) and Biopython (https://biopython.org/) tools were used to predict the physicochemical properties (net charge, isoelectric point, hydrophobicity, amphiphilicity) and spatial structures (alpha helix ratio, beta sheet ratio) of AMP and non‐AMP sequences (Figure 2d).

### 4.7 Data Distribution

The token sequences and PCSS descriptors from AMPs (4656 samples, 51.1%) and non‐AMPs (4458 samples, 48.9%) were divided into training set (2793 AMPs and 2674 non‐AMPs), validation set (931 AMPs and 892 non‐AMPs), and test set (932 AMPs and 892 non‐AMPs) according to a ratio of 6:2:2 (Figure 2t), with the train_test_split function in scikit‐learn (https://scikit‐learn.org/stable/index.html). Similarly, samples from different AMP databases were also allocated into the training set (APD6: 783, CAMPR4: 834, DRAMP: 612, dbAMP: 562), validation set (APD6: 262, CAMPR4: 278, DRAMP: 204, dbAMP: 187), and test set (APD6: 262, CAMPR4: 278, DRAMP: 205, dbAMP: 187) by the same random splitting strategy with a 6:2:2 ratio (Figure 2u).

### 4.8 ClaAMP Model

The ClaAMP model is sequentially composed of an input layer (input dimension: 32), an embedding layer (input dimension: 23, output dimension: 128), two feature extraction modules, two BiLSTM layers (unit count: 64), an attention layer (head count: 8, head size: 32), an add layer that can merge the outputs from the second BiLSTM layer and the attention layer, a global average pooling layer, a dense layer (output dimension: 256, activation function: ReLU) and a dense output layer (output dimension: 1, activation function: sigmoid). Two feature extraction modules include a CNN layer (kernel count: 128, kernel size: 3, stride: 1, activation function: ReLU, padding mode: same), a maxpooling layer (pool size: 2, stride: 1), and a dropout layer (dropout probability: 0.2).

(1) $$Sigmoid_{i}=\frac{1}{1+e^{-logit_{i}}}$$

(2) $$\mathrm{Clas}s_{i\;}=\left\{\begin{array}{c} \mathrm{AMP},\;\mathrm{Sigmoi}d_{i}\;\geq \;T\\ \mathrm{non}-\mathrm{AMP},\;\mathrm{Sigmoi}d_{i}\;<\;T \end{array}\right.$$

where *i* refers to the samples number, *Sigmoid_i_* refers to the sigmoid value of *i*th sample, *logit_i_* refers to the logit output of *i*th sample, and *T* refers to the sigmoid threshold. *Class*_*i* _ refers to the class of *i*th sample.

The ClaAMP model was training using the adaptive moment estimation (Adam) optimizer with an initial learning rate of 0.0001. The BCE was used as the loss function, and the AUC was used as the evaluation metric. Since the sigmoid threshold is variable during training, the AUC is chosen as the evaluation metric, which can remain independent of the sigmoid threshold. A learning rate scheduler was implemented to automatically reduce the learning rate by 90% when the validation loss plateaued for 10 consecutive epochs. The model was trained for 50 epochs with a batch size of 256.

In addition, accuracy, precision, recall, and F1 score were used to further evaluate the performance of the ClaAMP model. Accuracy can reflect the overall proportion of correctly classified samples, representing the global predictive correctness. Precision can quantify the proportion of true positives among all predicted positive results, indicating the reliability of positive predictions. Recall can measure the proportion of actual positive samples that are correctly identified, reflecting the sensitivity to true positive instances. F1 score can balance precision and recall as the harmonic mean, providing a robust measure when dealing with imbalanced datasets.

(3) $$BCE\;=-\frac{1}{n}\sum_{i}^{n}(y_{i}\cdot \log\left(p\left(y_{i}\right)\right)+(1-y_{i})\cdot \log(1-p\left(y_{i}\right)))$$

where *i* refers to the samples number (*i* = 1, 2, 3, …, *n*), *y_i_* refers to the true class, and *p*(*y_i_*) refers to the probability of predicting a positive result.

(4) $$TPR=\frac{TP}{TP+FN}$$

(5) $$FPR=\frac{FP}{FP+TN}$$

(6) $$AUC=\int_{0}^{1}TPR\left(FPR\right)d(FPR)$$

where *TP* refers to the number of samples belonging to category *i* that are predicted to be category *i*, *TN* refers to the number of samples not belonging to category *i* that are not predicted to be category *i*, *FP* refers to the number of samples not belonging to category *i* that are predicted to be category *i*, and *FN* refers to the number of samples belonging to category *i* that are not predicted to be category *i*.

(7) $$\mathrm{Accuracy}=\frac{1}{n}\sum_{i}^{n}\frac{\mathrm{TP}+\mathrm{TN}}{\mathrm{TP}+\mathrm{TN}+\mathrm{FP}+\mathrm{FN}}$$

(8) $$\mathrm{Precision}=\frac{1}{n}\sum_{i}^{n}\frac{\mathrm{TP}}{\mathrm{TP}+\mathrm{FP}}$$

(9) $$\mathrm{Recall}=\frac{1}{n}\sum_{i}^{n}\frac{\mathrm{TP}}{\mathrm{TP}+\mathrm{FN}}$$

(10) $$F1\;\mathrm{score}=\frac{1}{n}\sum_{i}^{n}2\frac{\mathrm{Precision}\;*\;\mathrm{Recall}}{\mathrm{Precision}+\mathrm{Recall}}$$

where *i* refers to the samples number (*i* = 1, 2, 3, …, *n*), and the relevant explanation of *TP*, *TN*, *FP* and *FN* can refer to Equations (4)—(6).

### 4.9 PreAMP Model

The PreAMP model was constructed based on the TL strategy, based on the ClaAMP model, adopting the same structure. However, the PreAMP model freezes the parameters of the embedding layer, the CNN layer, the maxpooling layer, and the dropout layer. The parameters of the BiLSTM layer, the attention layer, the add layer, the global average pooling layer, and the dense layer remain trainable. The output layer was modified to contain 6 units, which exactly correspond to net charge, isoelectric point, hydrophobicity, amphiphilicity, alpha helix ratio, beta sheet ratio, and a linear activation function to predict 6 PCSS descriptors.

The PreAMP model was optimized using the Adam optimizer with a learning rate of 0.0001. The MSE was used as the loss function, and the *R* ^2^ was employed as the scoring function. The PreAMP model was trained for 100 epochs with a batch size of 128.

To assess the predictive performance of the PreAMP model, both MSE and R^2^ were calculated. The MSE can measure the average magnitude of prediction errors by calculating the mean squared difference between the predicted and actual values, reflecting overall predictive capability. The *R* ^2^ can quantify the proportion of variation in actual values explained by the model, indicating the extent to the model captures underlying data patterns. In addition, the predicted error (PE) was further utilized to compute the sample distribution of errors between true and predicted values.

(11) $$MSE=\frac{1}{n}\sum_{i}^{n}{\left(y_{i}-\hat{y_{i}}\right)}^{2}$$

(12) $$\;R^{2}=1\;-\;\frac{\sum_{i\;}^{n}{\left(y_{i}\;-\;\hat{y_{i}}\right)}^{2}}{\sum_{i\;}^{n}{(y_{i}\;-\;\bar{y})}^{2}}$$

(13) $$\mathrm{PE}=y_{i}-\hat{y_{i}}$$

where *i* refers to the samples number (*i* = 1, 2, 3, …, *n*), *y_i_* is the true value of the *i*th sample, $\hat{y_{i}}$ is the predicted value of the *i*th sample, and $\bar{y}$ is the average true value of all samples.

### 4.10 GenMTAMP Model

The GenMTAMP model primarily includes an encoder, a decoder, and a conditional predictor. The encoder (Figure 6a) receives two input ports referring to the token sequences and the PCSS descriptors. First, the sequence tokens were transformed by an embedding layer (input dimension: 23, output dimension: 128). In parallel, the PCSS descriptors were processed sequentially through a dense layer (output dimension: 128) followed by a repeatvector layer to match the temporal dimension of the embedded sequences. Then, the two representations were fused using an add layer. Next, the fused features were passed through two BiLSTM layers (unit count: 128), producing the contextualized sequence representation. Finally, the output results were mapped by two parallel dense layers (output dimension: 64) to estimate mean and SD of the latent distribution. The latent vector (*z*) was sampled from the reparameterized Gaussian distribution.

(14) z=μ+σ⊙ε,ε∼N(0,1)

where μ and σ refer to the mean and SD vectors output by the encoder for each sample, ε is a random vector drawn from the standard normal distribution, and ⊙ denotes element‐wise multiplication.

The decoder (Figure 6b) is sequentially composed of a concatenation module, two state initialization dense layers for the initial hidden and cell states, an embedding layer, two LSTM layers, and a dense output layer. The latent vector *z* and the PCSS descriptors are concatenated along the feature dimension. Then, two independent dense layers (output dimension: 64, activation function: ReLU) are used to generate the initial hidden state and cell state for the subsequent LSTM layers. The decoder incorporates an embedding layer (input dimension: 23, output dimension: 128) to represent the previously generated tokens during autoregressive decoding. The embedding is processed through two stacked LSTM layers (unit count: 64). The first LSTM layer outputs full temporal sequences, while the second LSTM layer returns only the final hidden state at each decoding step to facilitate step‐wise generation. The final dense layer outputs a dimension of 23, including AA types, padding, start, and end tokens, generating the token‐level logits required for autoregressive sequence generation.

The conditional predictor (Figure 6c) adopts the same architecture as the PreAMP model, including an input layer, an embedding layer, two CNN blocks comprising a CNN layer, a maxpooling layer, and a dropout layer, two BiLSTM layers, an attention layer, an add layer, a global average pooling layer, and two dense layers. In addition, all hyperparameters and architectural configurations remain consistent with the PreAMP model.

The GenMTAMP model was optimized using Adam with a learning rate of 0.001. The GenMTAMP model was fitted using a batch size of 256 and a training epoch count of 100. The loss function is a total loss composed of the RS loss, the KLD loss, and the PCSS loss weighted together. The RC loss can evaluate the ability to accurately reconstruct sequences from latent representations. The KLD loss can regularize the latent space by aligning the posterior with the prior distribution, promoting smooth and continuous latent representations. The PCSS loss can enforce consistency between generated sequences and their target PCSS descriptors. The SR accuracy and CP accuracy were used for forward evaluation of model performance. The SR accuracy can reflect the ability to learn the target sequence, serving as a measure of reconstruction quality. The CP accuracy can assess whether generated sequences satisfy predefined conditions in PCSS descriptors, acting as a key indicator for evaluating conditional consistency and directional generation capability.

(15) $$\mathrm{CE}\left(x_{t},\;{\hat{y}}_{t}\right)=-\sum_{k}^{K}1_{\left[x_{t}=k\right]}\log({\hat{y}}_{t,\;k})$$

(16) $$RC=\frac{\sum_{t}^{T}1_{\left[x_{t}\neq 0\right]}CE(x_{t},\;\;{\hat{y}}_{t})}{\sum_{t=1}^{T}1_{\left[x_{t}\neq 0\right]}+\varepsilon }$$

(17) $$KLD=-\frac{1}{2}\cdot \frac{1}{N}\sum_{n}^{N}\sum_{d}^{D}(1+\log\sigma_{n,\;i}^{2}\;-\;\mu_{n,\;i}^{2}\;-\;\sigma_{n,\;i}^{2})$$

(18) $$PCSS=\frac{1}{N}\sum_{n}^{N}\sum_{p}^{P}w_{p}{\left(c_{n,\;p}\;-\;{\hat{c}}_{n,\;p}\right)}^{2}$$

(19) Totalloss=α∗RC+β∗KLD+γ∗PCSS

where *t* refers to the time step number (*t* = 1, 2, 3, …, *T*), *k* refers to the AA type (*k* = 1, 2, 3, …, *K*), *x_t_* refers to the true token at *t*th time step, ${\hat{y}}_{t,\;k}$ the probability of predicting the *k*th AA type, ε is added to avoid division by zero, *n* refers to the batch number (*n* = 1, 2, 3, …, *N*), *d* refers to the latent space dimension number (*d* = 1, 2, 3, …, *D*), $\sigma_{n,\;i}^{2}$ refers to the mean of the *d*th latent space dimension for the *n*th sample, $\mu_{n,\;i}^{2}$ refers to the SD of *d*th latent space dimension for the *n*th sample, *p* refers to the PCSS number (*p* = 1, 2, 3, …, *P*), w_*p*_ refers to the weight of *p*th PCSS descriptor, *c*_*n*, *p*_ refers to the true value of the *p*th PCSS descriptor for the *n*th sample, ${\hat{c}}_{n,\;p}$ refers to the true value of the *p*th PCSS descriptor for the *n*th sample, and α, β, and γ refer to the weight coefficients for the *RC*, *KLD* and *PCSS* losses, respectively.

(20) $$m_{n,t}=\left\{\begin{array}{c} 1,\;x_{n,t}\;\mathrm{is}\;\mathrm{not}\;0\\ 0,\;x_{n,t}\;\mathrm{is}\;0 \end{array}\right.$$

(21) $$SR=\frac{\sum_{n}^{N}\sum_{t}^{T}m_{n,\;t}\cdot 1_{\left[{\hat{x}}_{n,\;t}=x_{n,\;t}\right]}}{\sum_{n}^{N}\sum_{t}^{T}m_{n,\;t}+\varepsilon }$$

(22) $$\mathrm{CP}=\frac{1}{p}\sum_{p=1}^{P}\frac{\frac{1}{N}\sum_{n}^{N}(c_{n,p}-{\bar{c}}_{p})({\hat{c}}_{n,p}-{\bar{\hat{c}}}_{p})}{\sigma_{c_{p}}\cdot \sigma_{{\hat{c}}_{p}}+\varepsilon }$$

where *n* refers to the batch number (*n* = 1, 2, 3, …, *N*), *t* refers to the time step number (*t* = 1, 2, 3, …, *T*),  *x*_*n*,*t*_ refers to the true token at *t*th time step for *n*th sequence, ${\hat{x}}_{n,\;t}$ refers to the reconstructed token at *t*th time step for *n*th sequence, ε is added to avoid division by zero, *p* refers to the PCSS number (*p* = 1, 2, 3, …, *P*), *c*_*n*,*p*_ refers to the true value of the *p*th PCSS descriptor for the *n*th sample, ${\bar{c}}_{p}$ refers to the mean of the true values for *N* samples, ${\hat{c}}_{n,\;p}$ refers to the predicted value of the *p*th PCSS descriptor for the *n*th sample, ${\bar{\hat{c}}}_{p}$ refers to the mean of the predicted values for *N* samples, $\sigma_{c_{p}}$ refers to the SD of the true values for *N* samples, $\sigma_{{\hat{c}}_{p}}$ refers to the SD of the predicted values for *N* samples, ε is added to avoid division by zero.

To further evaluate the performance of the GenMTAMP model, four representative published AMP generative models, including PepVAE [37], KPPepGen [38], AMP‐Designer [13], and HydrAMP [11], were introduced for comparative analysis. For each model, the same number of peptide sequences as those generated by the GenMTAMP model was generated. Then, all generated sequences were subjected to a unified downstream screening pipeline. Specifically, the generated peptides were first screened by the ClaAMP model. Subsequently, the sequences predicted as AMPs were input into the PreAMP model to predict six PCSS descriptors, including net charge, isoelectric point, hydrophobicity, amphiphilicity, alpha helix, and beta sheet. This procedure enabled a consistent comparison of the downstream quality of peptides generated by different models under the same evaluation standards.

### 4.11 MTAMP Generation

The detailed steps for generating sequences using MTAMP sampling are as follows: (1) Input the six PCSS descriptors and the normal distribution sampled from the latent space into the decoder of the GenMTAMP model to generate probability distribution. (2) Map probability distributions to tokens based on greedy sampling, and generate the next probability distribution using stepwise autoregressive method based on the token predicted in the previous step. (3) Process probability distributions using temperature sampling and top‐k sampling. (4) Inverse tokens to AA sequences as MTAMPs. (5) Input the generated MTAMPs into the ClaAMP model to identify whether they are AMPs. (6) Input the generated MTAMPs identified as AMPs into the PreAMP model to predict the PCSS descriptors. (7) Quantify the performance of the generated MTAMPs based on a weighted score (WS). This strategy tightly couples the generation model with downstream functional evaluation, providing an effective paradigm for achieving high‐fidelity de novo design of MTAMPs.

(23) $$Score_{i}\left(pcss_{i,\;j}\right)=\left\{\begin{array}{c} e^{-{\left(\frac{\;{\bar{pcss}}_{i,\min}-pcss_{i,j}}{{\bar{pcss}}_{i,\max}-{\bar{pcss}}_{i,\min}}\right)}^{2}},\;pcss_{i,j}<{\bar{pcss}}_{i,\min}\\ 1,{\bar{pcss}}_{i,\min}<pcss_{i,j}<{\bar{pcss}}_{i,\max}\\ e^{-{\left(\frac{pcss_{i,j}-{\bar{pcss}}_{i,\max}}{{\bar{pcss}}_{i,\max}-{\bar{pcss}}_{i,\min}}\right)}^{2}},\;pcss_{i,j}>{\bar{pcss}}_{i,\max} \end{array}\right.$$

(24) $$WS=\frac{\sum_{i}w_{i}Score_{i}\left(pcss_{i,\;j}\right)}{\sum_{i}w_{i}}$$

where *i* refers to the PCSS descriptors (*i* = net charge, isoelectric point, hydrophobicity, amphiphilicity, alpha helix ratio, and beta sheet ratio), *j* refers to the number of generated MTAMPs, *pcss*_*i*, *j*_ refers to the *i*th PCSS description of the *j*th generated MTAMP, $\;{\bar{pcss}}_{i,\;\min}$ refers to the 1/4 boundary value of the *i*th PCSS description, $\;{\bar{pcss}}_{i,\;\max}$ refers to the 3/4 boundary value of the *i*th PCSS description, *w_i_* refers to the weights of the *i*th PCSS description.

### 4.12 Fmoc Solid‐Phase Peptide Synthesis

All peptides were synthesized using Fmoc‐SPPS (Sangon Biotech (Shanghai) Co., Ltd., Shanghai, China). The molecular weight (MW) and purity of the synthesized peptides were characterized by liquid chromatography‐mass spectrometry (LC‐MS) and high‐performance liquid chromatography (HPLC), respectively. Each peptide is successfully synthesized with a MW error of less than 0.1 wt.% and a purity exceeding 95% (Table S13). The synthesized samples were stored as lyophilized powder at −20°C in a light‐protected environment. In addition, an AMP (Magainin II) purchased from Shanghai Macklin Biochemical Co., Ltd. (Shanghai, China) was used as the positive control.

### 4.13 Minimum Inhibitory Concentration

Four Gram‐negative bacteria, referring to *E. coli* ATCC 25922, *P. aeruginosa* ATCC 27853, *S. typhimurium* CMCC (B) 50115, and *A. baumannii* ATCC 19606, two Gram‐positive bacteria referring to *S. aureus* ATCC 25923 and *B. subtilis* ATCC 9372, and one fungus referring to *C. albicans* ATCC 10231 were purchased from Guangdong Microbial Culture Collection Center (GDMCC) (Guangzhou, China). The four Gram‐negative bacteria and two Gram‐positive bacteria were cultured in Luria‐Bertani (LB) medium at 37°C with shaking at 200 rpm until reaching the logarithmic growth stage. The fungus was cultured in Sabouraud dextrose broth at 37°C with shaking at 200 rpm. Then, the microbial suspension was diluted 100‐fold with Mueller‐Hinton broth (MHB) to achieve a final inoculum of approximately 5 × 10^5^ CFU/mL. The peptide was dissolved in phosphate‐buffered saline (PBS) to prepare a stock solution at a concentration of 1024 µg/mL. As shown in Figure 8a, 100 µL of MHB was added to each well of a 96‐well plate, and 100 µL of the peptide stock solution was added to the first well. A series of peptide solutions with decreasing concentrations was prepared in the 96‐well plate using a two‐fold dilution method. Subsequently, 100 µL of microbial suspension was added to each well containing a peptide dilution, adjusting the final volume to 200 µL per well. A blank group was established containing only 100 µL of the peptide concentration and 100 µL of MHB. A negative control group containing only 100 µL of microbial suspension and 100 µL of MHB was also established. After incubating the plates at 37°C for 16 h, the OD_600_ values were measured using a microplate reader (SpectraMax Mini, Molecular Devices Shanghai Corporation, Shanghai, China). All experiments were performed in triplicate.

### 4.14 Cell Cytotoxicity

The Cell Counting Kit 8 (CCK‐8) assay was used to evaluate the cell cytotoxicity of peptides against mammalian cells. As shown in Figure 8b, approximately 4000 GES‐1 cells were seeded into sterile 96‐well cell culture plates and cultured at 37°C with 5% CO_2_ for 24 h. The peptide dissolved in PBS was diluted in medium to form multiple concentration gradients ranging from 4 to 256 µg/mL. The medium served as the blank group, and the cell suspension acted as the control group. Subsequently, 10 µL of CCK‐8 reagent (Shanghai Aladdin Biochemical Technology Co., Ltd., Shanghai, China) was added to each well and incubated at 37°C for 2 h. Finally, the OD_450_ was measured using a microplate reader (SpectraMax Mini, Molecular Devices Shanghai Corporation, Shanghai, China). The CCK‐8 assay was performed in triplicate. The cell viability (*CV*) was calculated using the following formula:

(25) $$\mathrm{CV}\;(\%)=\frac{OD_{\mathrm{Sample}\;\mathrm{group}}-OD_{\mathrm{Blank}\;\mathrm{group}}}{OD_{\mathrm{Control}\;\mathrm{group}}\;-\;OD_{\mathrm{Blank}\;\mathrm{group}}}\times 100\%$$

where *OD*_*Sample* *group*_ refers to the OD value of the sample at 450 nm, *OD*_*Blank* *group*_ refers to the OD value of the blank group at 450 nm, and *OD*_*Control* *group*_ refers to the OD value of the control group at 450 nm.

### 4.15 Cell Haemolysis

Fresh rabbit blood (Nanjing Senbeijia Biotechnology Co., Ltd., NanJing, Jiangsu) was centrifuged at 3000 rpm for 10 min until the supernatant became clear to wash away the culture medium. Then, the cells were resuspended in PBS to prepare a 2% rabbit red cell suspension. Peptide stock solutions with different volumes were added to 80 µL of the 2% rabbit red cell suspension. Subsequently, all samples were supplemented with PBS to a final volume of 280 µL, achieving final peptide concentrations of 4, 8, 16, 32, 64, 128, and 256 µg/mL. The PBS and Triton X‐100 PBS (2%) were used as negative and positive controls, respectively (Figure 8c). Samples were incubated at 37°C in a water bath for 1 h, then centrifuged at 3000 rpm for 10 min. Finally, 200 µL of supernatant was transferred to a 96‐well plate, and the OD_545_ was measured using a microplate reader (SpectraMax Mini, Molecular Devices Shanghai Corporation, Shanghai, China). Three replicates were set for each sample. The haemolysis rate (*HR*) for each sample was calculated using the following formula:

(26) $$\mathrm{HR}\;(\%)=\frac{OD_{\mathrm{Sample}\;\mathrm{group}}\;-\;OD_{\mathrm{Negative}\;\mathrm{group}}}{OD_{\mathrm{Positive}\;\mathrm{group}}\;-\;OD_{\mathrm{Negative}\;\mathrm{group}}}\times 100\%$$

where *OD*_*Sample* *group*_ refers to the OD value of the sample at 545 nm, *OD*_*Negative* *group*_ refers to the OD value of the negative group at 545 nm, and *OD*_*Positive* *group*_ refers to the OD value of the positive group at 545 nm.

### 4.16 Molecular Dynamics Simulation

The CHARMM‐GUI service [39] about Membrane Builder was used to construct the peptide‐phospholipid bilayer simulation system. The complete workflow (https://charmm‐gui.org/?doc=input/membrane.bilayer) is summarized below:

Step 1: Structure coordinates of the peptide molecule. The three‐dimensional coordinates (PDB files) of peptides were generated using the AlphaFold2 service [8].
Step 2: Orientation of the peptide molecule. The peptide molecule was reoriented by aligning the principal structural axis with the *z*‐axis, followed by a 90° rotation around the *y*‐axis. Subsequently, the molecule was translated upward by 3.00 nm along the *z*‐axis to ensure an appropriate initial position relative to the membrane surface.
Step 3: Specification of system dimensions. The simulation box (length × width × height: 10 × 10 × 10 nm) included a planar lipid bilayer composed of 120 molecules of 1‐palmitoyl‐2‐oleoyl‐sn‐glycero‐3‐phosphoethanolamine (POPE) and 60 molecules of 1‐palmitoyl‐2‐oleoyl‐sn‐glycero‐3‐(phospho‐rac‐(1‐glycerol)) (POPG). The water phase fully occupied the region extending 2.25 nm above and below the bilayer midplane.
Step 4: Construction of system components. The water phase was supplemented with K^+^ and Cl^−^ to achieve a physiological concentration of 0.15 mol/L to neutralize the total charge of the system.
Step 5: Assembly of the simulation system. The CHARMM‐GUI service can automatically assemble the peptide molecule, lipid bilayer, water, and ions into a unified system while evaluating protein surface penetration and lipid ring penetration.
Step 6: System equilibration. The final system was energy‐minimized and subsequently equilibrated in the isothermal‐isobaric (NPT) ensemble for 5 ns using the CHARMM36 force field as implemented in the CHARMM‐GUI service. Particle‐Mesh Ewald (PME) parameters were generated automatically, and the temperature was maintained at 310.0 K throughout the equilibration process.

Finally, the constructed peptide‐phospholipid bilayer structure was subjected to the MDS using the GROMACS program [40]. Default simulation settings were implemented in CHARMM‐GUI for the selected force field combination and periodic boundary conditions. The workflow included energy minimization, followed by six sequential equilibration stages with gradually released positional restraints on both peptides and lipid molecules.

In the current research, the RMSD was primarily employed to assess the stability of the peptide and the phospholipid bilayer. The DSSP program [41] was further utilized to evaluate the structural stability of the peptide alpha helix. Furthermore, the interaction between the peptide and the phospholipid bilayer was further assessed by measuring the depth of peptide insertion into the membrane along the z‐axis.

(27) $$\mathrm{RMSD}(t)=\sqrt{\frac{1}{N_{\mathrm{RMSD}}}\sum_{i=1}^{N_{\mathrm{RMSD}}}∥x_{i}\left(t\right){-x}_{i}^{\mathrm{ref}}{∥}^{2}}$$

where *t* is the frame number, *RMSD*(*t*) is the RMSD at *t*th frame, *i* is the atom number (*i* = 1, 2, 3, …, *N_RMSD_*), *x_i_* (*t*) is the positions of *i*th atom at *t*th frame, $x_{i}^{ref}$ is the positions of *i*th atom at first frame, and ‖ · ‖ is the Euclidean distance.

(28) $$\mathrm{Alpha}\;\mathrm{heli}x_{i}\left(t\right)=\left\{\begin{array}{c} 1,\;\mathrm{if}\;\mathrm{DSS}P_{i}(t)\;\in \;\left\{H\right\}\;\\ 0,\;\mathrm{otherwise} \end{array}\right.$$

(29) $$\mathrm{Alpha}\;\mathrm{heli}x_{0-1}\left(t\right)=\frac{1}{N_{\mathrm{helix}}}\sum_{i=1}^{N_{\mathrm{helix}}}\mathrm{Alpha}\;\mathrm{heli}x_{i}\left(t\right)$$

where *t* is the frame number, *i* is the atom number (*i* = 1, 2, 3, …, *N_helix_*),  *DSSP_i_* (*t*) is the secondary structure type, *H* is the alpha helix conformation, *Alpha* *helix_i_* (*t*) is the alpha helix ratio of *i*th atom at *t*th frame, and *Alpha* *helix*_0‐1_(*t*) is the normalized alpha helix ratio at *t*th frame.

(30) $$\mathrm{Distance}\left(t\right)=\frac{1}{N_{\mathrm{mem}}}\sum_{i=1}^{N_{\mathrm{mem}}}z_{i}^{\mathrm{mem}}(t)\;-\;\frac{1}{N_{\mathrm{pep}}}\sum_{j=1}^{N_{\mathrm{pep}}}z_{j}^{\mathrm{pep}}\left(t\right)$$

where *t* is the frame number, *Distance*(*t*) is the relative distance between the peptide and the membrane center in the Z‐axis at *t*th frame, *i* is the membrane atom number (*i* = 1, 2, 3, …, *N_mem_*), *j* is the peptide atom number (*i* = 1, 2, 3, …, *N_pep_*), $z_{i}^{mem}(t)$ is the z‐coordinate of *i*th membrane atom at *t*th frame, $z_{j}^{pep}(t)$ is the z‐coordinate of *i*th peptide atom at *t*th frame.

### 4.17 Circular Dichroism Spectrum

The 1 mg lyophilized peptide powder was dissolved in 1 mL ultrapure water to obtain a 1 mg/mL stock solution. The 0.1 mg/mL ultrapure water‐based peptide solution was prepared by diluting 0.1 mL of the stock solution with 0.9 mL ultrapure water. In addition, the 0.1 mg/mL stock solution was sequentially diluted with 0.4 mL ultrapure water and 0.5 mL TFE solution to obtain a 0.1 mg/mL peptide solution containing 50% TFE. Subsequently, CDS for both peptide solution systems and corresponding blank controls were measured using a circular dichroism spectrometer (Chirascan V100, Applied Photophysics Ltd., Leatherhead, United Kingdom). The 200 µL solution was injected into a 1 mm path length quartz cell to measure CDS within the 190–260 nm. The scanning speed was 200 nm/min with a bandwidth of 1 nm. Finally, the spectral data were processed using the mean moving smoothing filter, and the proportion of secondary structures (alpha helix ratio, beta sheet ratio, beta turn ratio, and random coil ratio) in peptides was calculated using the CDNN software.

### 4.18 Scanning Electron Microscope

*E. coli* ATCC 25922 was cultured in LB medium at 37°C until the logarithmic growth phase, and the bacterial solution was diluted to 1×10^8^ CFU/mL. The bacterial suspension was treated with peptides at a final concentration of 1×MIC for 16 h at 37°C, with untreated *E. coli* ATCC 25922 serving as the control group. Samples were centrifuged at 4°C at 4000 rpm for 5 min. The supernatant was discarded, and the pellet was resuspended in PBS, repeated three times. Then, the bacterial pellet was resuspended in 2.5% glutaraldehyde solution and incubated overnight at 4°C. The centrifuged bacterial pellet was sequentially resuspended in 30%, 50%, 70%, 90%, and 100% ethanol solutions and dehydrated for 10 min. Finally, samples were observed using the SEM (Merlin, Carl Zeiss Vision (China) Ltd., Guangzhou, China) after critical CO_2_ drying and gold sputtering coating.

### 4.19 Confocal Laser Scanning Microscope

*E. coli* ATCC 25922 were grown in LB medium at 37°C to the logarithmic phase and subsequently adjusted to a final density of 1×10^8^ CFU/mL. The bacterial suspension was incubated with peptides at a concentration corresponding to 1×MIC for 16 h at 37°C, while untreated bacteria were used as the negative control. Following incubation, the samples were centrifuged at 4°C and 4000 rpm for 5 min. The resulting pellets were collected, washed three times with PBS, and then resuspended in a SYTO‐9/PI (Shanghai Macklin Biochemical Co., Ltd.) staining solution. After incubation at 25°C for 15 min in the dark, the stained bacteria were examined using CLSM (Leica STELLARIS 5, Leica Microsystems Shanghai Ltd., Wetzlar, Germany). All experiments were repeated at least three times.

### 4.20 Statistical Analysis

Data analysis methods, including sequence cleaning, sequence encoding, PCSS descriptor acquisition, data distribution, model construction (ClaAMP, PreAMP, GenMTAMP), and MTAMP sampling generation were performed using Python 3.8 within a software environment configured with Tensorflow 2.11 (https://www.tensorflow.org/), Keras 2.11 (https://keras.io/), Scikit‐learn 1.3 (https://scikit‐learn.org/stable/), Numpy 1.23 (https://numpy.org/), Pandas 2.0 (https://pandas.pydata.org/), Scipy 1.10 (https://scipy.org/), Matplotlib 3.4 (https://matplotlib.org/). All workflows were executed in Jupyter Notebook (https://jupyter.org/). Model training and evaluation were conducted on a workstation equipped with a single NVIDIA RTX 2080 Ti GPU (11 GB VRAM), 12 vCPUs (Intel Xeon Platinum 8255C @ 2.50 GHz), and 40 GB system memory. The figures used to present the research results were drawn using the Origin 2021 (https://www.originlab.com/) and Adobe Illustrator 2021 (https://www.adobe.com/products/illustrator.html).

## Author Contributions

**Da‐Wen Sun**: funding acquisition, resources, writing – review and editing, supervision. **Hongbin Pu**: funding acquisition, investigation, resources. **Yibing Zhang**: investigation. **Jidong Tang**: investigation. **QingYi Wei**: investigation, resources, funding acquisition. **Jingxiao Yu**: writing – original draft, formal analysis, investigation.

## Conflicts of Interest

The authors declare no conflicts of interest.

## Supporting information




**Supporting File**: advs75928‐sup‐0001‐SuppMat.docx.

## Data Availability

The main data supporting the findings of this study are available within the Article and the Supplementary Information. Our study contains publicly available AMP and non‐AMP data. The AMP data were mainly collected from four public AMP datasets: APD6 (https://aps.unmc.edu/AP/), CAMPR4 (https://camp.bicnirrh.res.in/index.php), DRAMP (http://dramp.cpu‐bioinfor.org/), and dbAMP (https://awi.cuhk.edu.cn/dbAMP/) prior to July 2025. The non‐AMP data were obtained from Uniport (https://www.uniprot.org/) before July 2025. All non‐AMP data containing keywords such as antimicrobial, antibacterial, antifungal, antiviral, antibiotic, antimalarial, antiparasitic, anticancer, defense, defensin, cathelicidin, histatin, bacteriocin, fungicide, toxin, effector, and excreted were excluded. In addition, external verification data were collected from APD6, CAMPR4, DRAMP, dbAMP, DBAASP (https://www.dbaasp.org/home), StarPep (https://starpepweb.org/), AMPSphere (https://ampsphere.big‐data‐biology.org/home), Uniport and relevant references. The relevant code related to this article is available in GitHub at https://github.com/JingxiaoYu1230/GenMTAMP.
