# Supplementary material for: De Novo Design of Membrane‐Targeting Antimicrobial Peptides Against Gram‐Negative Bacteria Using a Generative Artificial Intelligence Framework
Source: Adv Sci (Weinh). 2026 Jun 2:e75928. Online ahead of print. doi: 10.1002/advs.75928 (PMC13336696; doi:10.1002/advs.75928)
Supplement: Supplementary file 1 — Supporting File: advs75928‐sup‐0001‐SuppMat.docx. [file ADVS-9999-e75928-s001.docx]

**Supplementary Information**

**De novo design of membrane-targeting antimicrobial peptides against Gram-negative bacteria using a generative artificial intelligence framework**

Jingxiao Yu^1,2,3^, Da-Wen Sun^1,2,3,4,^[[1]](#footnote-1)^*^, Qingyi Wei^1,2,3^, Yibin Zhang^1,2,3^, Jidong Tang^1,2,3^, Hongbin Pu^1,2,3,*^

^1^ School of Food Science and Engineering, South China University of Technology, Guangzhou 510641, China

^2^ Academy of Contemporary Food Engineering, South China University of Technology, Guangzhou Higher Education Mega Centre, Guangzhou 510006, China

^3^ Engineering and Technological Research Centre of Guangdong Province on Intelligent Sensing and Process Control of Cold Chain Foods, & Guangdong Province Engineering Laboratory for Intelligent Cold Chain Logistics Equipment for Agricultural Products, Guangzhou Higher Education Mega Centre, Guangzhou 510006, China

^4^ Food Refrigeration and Computerized Food Technology (FRCFT), Agriculture and Food Science Centre, University College Dublin, National University of Ireland, Belfield, Dublin 4, Ireland

**
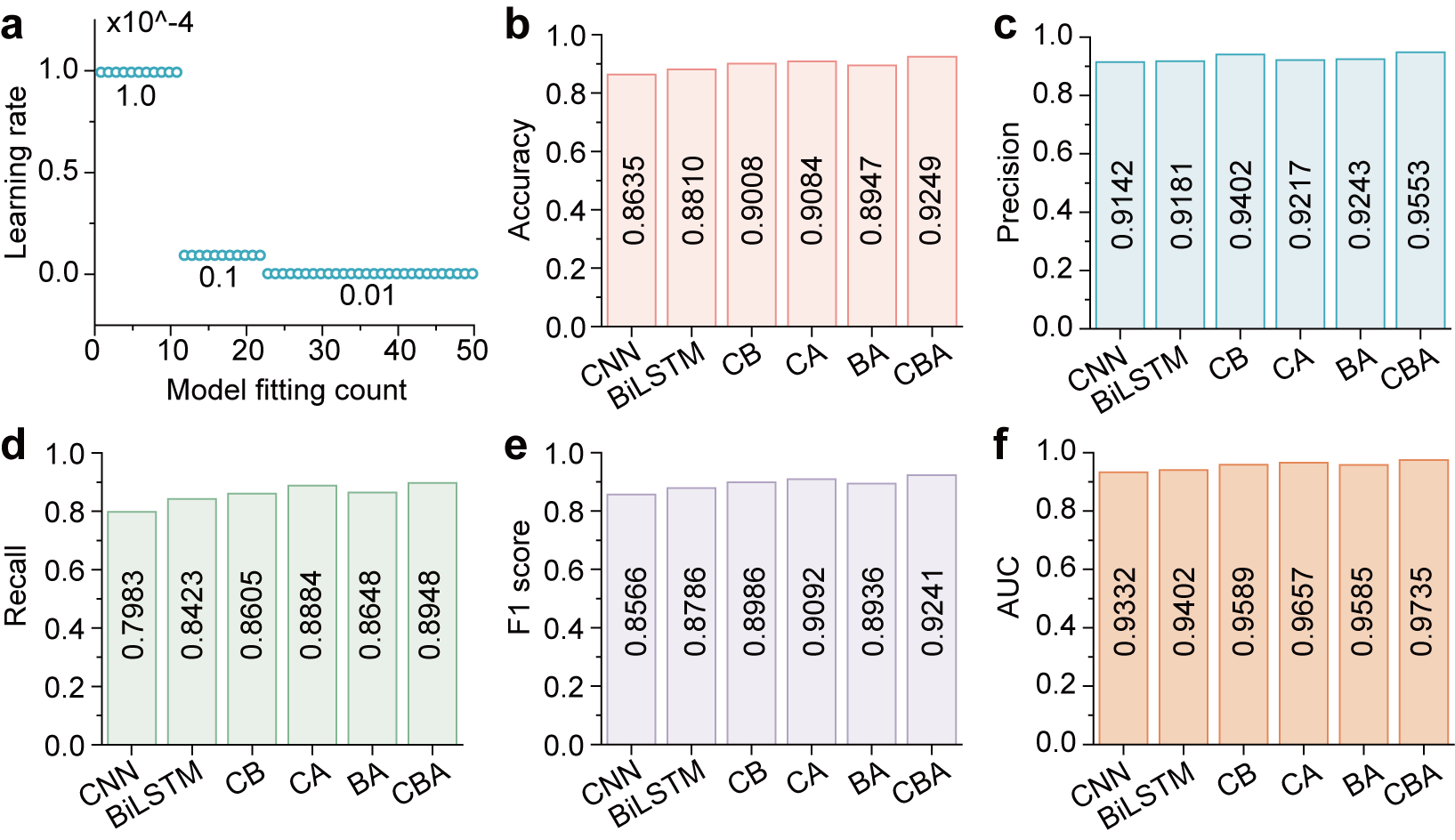
**

**Fig. S1: Learning rate and ablation experiments for the ClaAMP model.** **a**, Learning rate during training process. A learning rate scheduler was implemented to automatically reduce the learning rate by 90% when the validation loss showed no decrease for 10 consecutive epochs. **b-f**, Accuracy (**b**), precision (**c**), recall (**d**), F1 score (**e**) and AUC (**f**) of different models in the ablation experiment. The parameters of same modules in different models were kept consistent. The CB model includes CNN and BiLSTM modules. The CA model includes CNN and Attention modules. The BA model includes BiLSTM and Attention modules. The CBA model refers to the ClaAMP model.


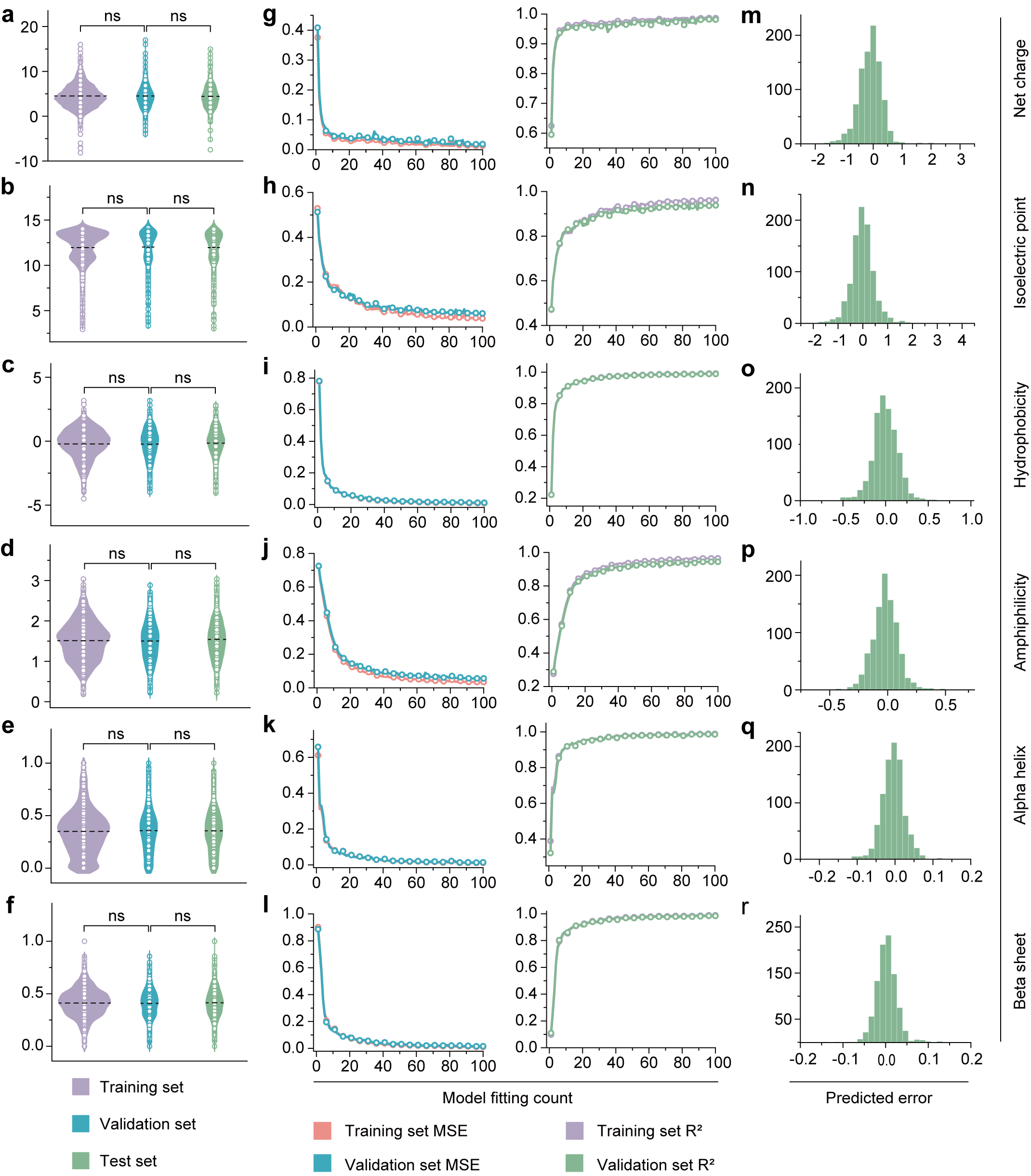


**Fig. S2:** **The data distribution, training process and predicted error of the PreAMP model.** **a-f**, Data distribution of net charge (**a**), isoelectric point (**b**), hydrophobicity (**c**), amphiphilicity (**d**), alpha helix ratio (**e**) and beta sheet ratio (**f**) for the training set, validation set, and test set. Black dashed lines indicate the mean, and ns denotes no significant difference (*p* < 0.0001). **g-l**, The MSE and R² of net charge (**g**), isoelectric point (**h**), hydrophobicity (**i**), amphiphilicity (**j**), alpha helix ratio (**k**) and beta sheet ratio (**l**). **m-r**, Predicted error of net charge (**m**), isoelectric point (**n**), hydrophobicity (**o**), amphiphilicity (**p**), alpha helix ratio (**q**) and beta sheet ratio (**r**). The vertical axis represents frequency.


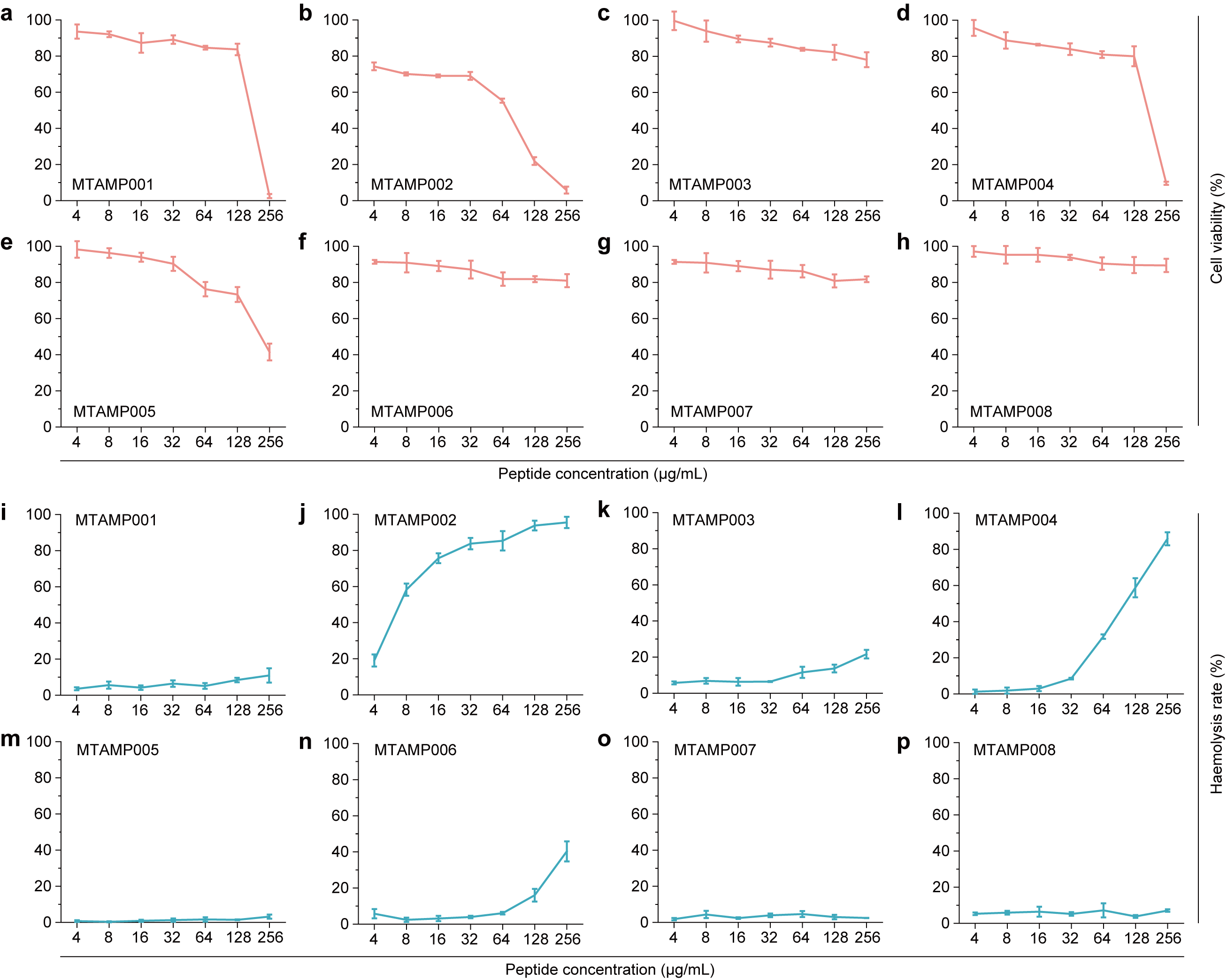


**Fig. S3: The cell toxicity and haemolysis of MTAMPs.** **a-h**, The toxicity of MATAMP001 (**a**), MTAMP002 (**b**), MTAMP003 (**c**), MTAMP004 (**d**), MTAMP005 (**e**), MTAMP006 (**f**), MTAMP007 (**g**) and MTAMP008 (**h**) target GES-1 cells. **i-p**, The haemolysis of MATAMP001 (**i**), MTAMP002 (**j**), MTAMP003 (**k**), MTAMP004 (**l**), MTAMP005 (**m**), MTAMP006 (**n**), MTAMP007 (**o**) and MTAMP008 (**p**) target rabbit red cells. All experiments were repeated three times in parallel.


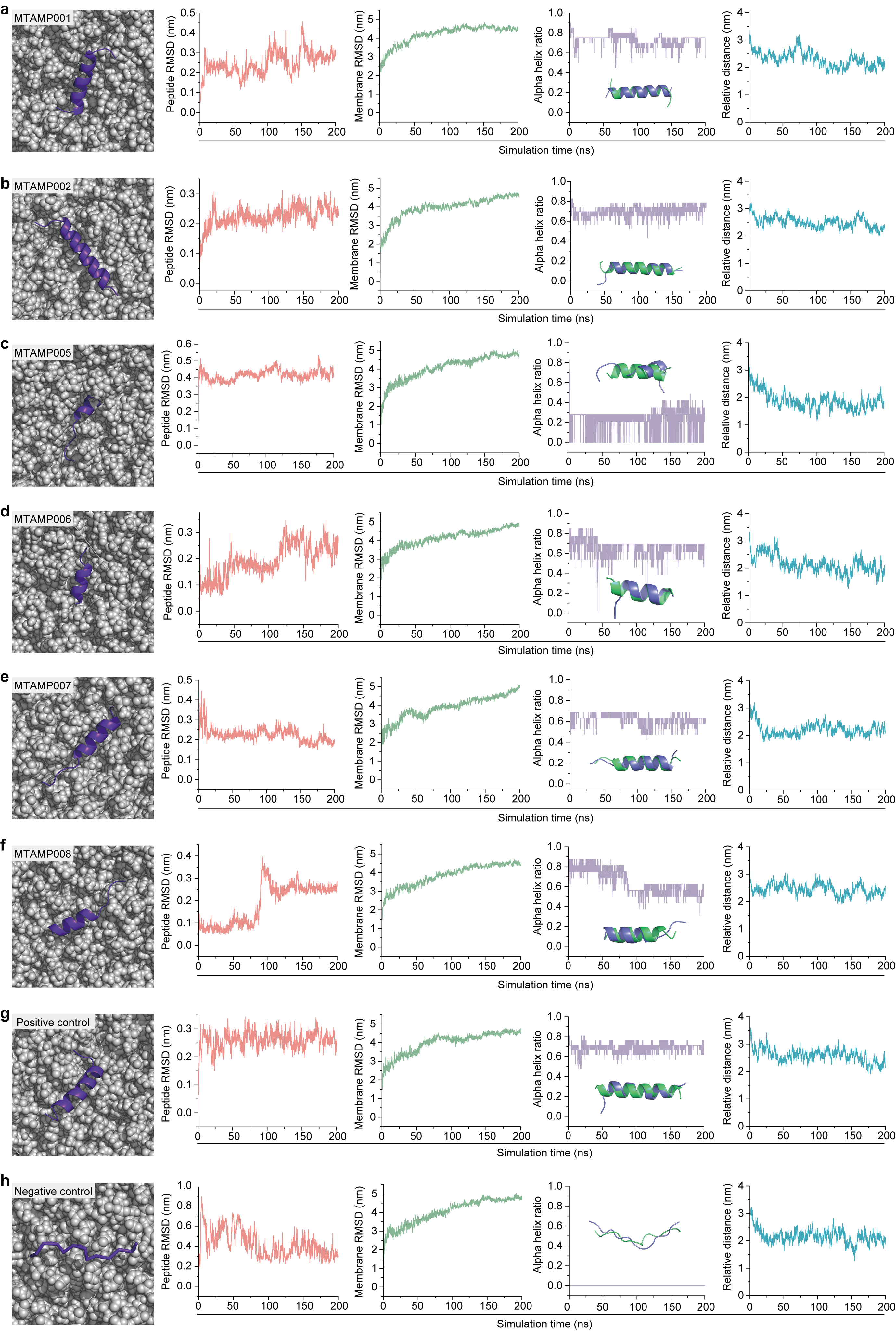


**Fig. S4: MDS results of MTAMPs** **interacting with bacterial outer membranes.** **a-h**, Schematic diagrams of molecular dynamics simulations, peptide RMSD, membrane RMSD, alpha helix ratio, and relative distance between peptide and membrane mass centre of MTAMP001 (**a**), MTAMP002 (**b**), MTAMP005 (**c**), MTAMP006 (**d**), MTAMP007 (**e**), MTAMP008 (**f**), positive control (**g**), and negative control (**h**). Purple peptides represent the peptides at 0 ns, while green peptides represent those at 200 ns.


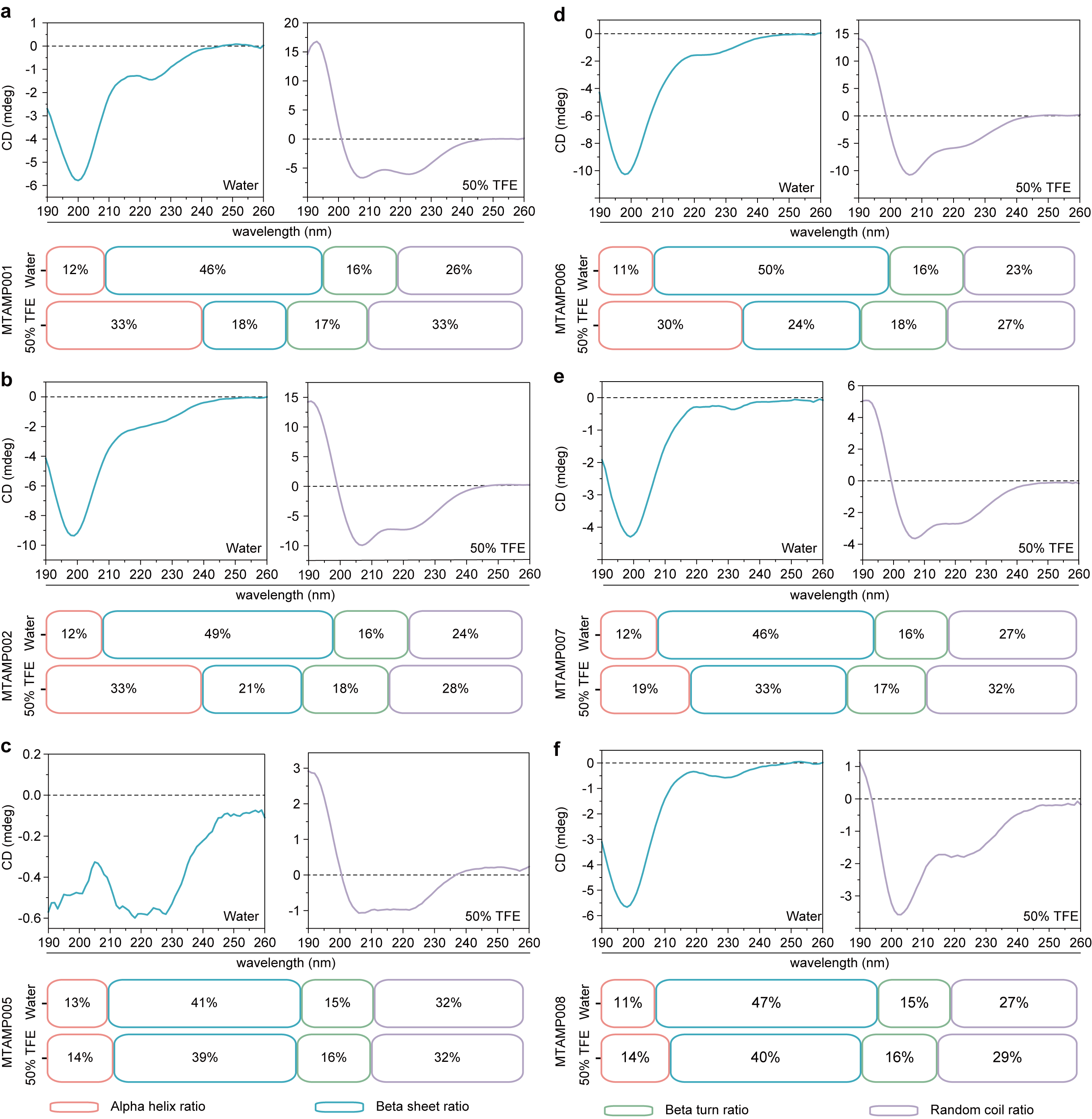


**Fig. S5: CDS and secondary structure ratio of MTAMPs**. **a-f**, CDS and secondary structure ratio of MTAMP001 (**a**), MTAMP002 (**b**), MTAMP005 (**c**), MTAMP006 (**d**), MTAMP007 (**e**), and MTAMP008 (**f**) in the water and 50% TFE. The black dashed line indicates the zero level.

**Table S1: KLD values for AMPs and non-AMPs using the UMAP method.**

| Modules | KLD values |
| --- | --- |
| Input | 0.1251 |
| Embedding | 0.8786 |
| CNN1 | 1.7172 |
| CNN2 | 2.6872 |
| BiLSTM1 | 3.7431 |
| BiLSTM2 | 5.1696 |
| Attention | 5.4587 |
| Dense | 6.3161 |

**Table S2: Extensive validation results of the ClaAMP model.**

| Sequences | True labels | Predicted labels | Sources |
| --- | --- | --- | --- |
| HGVSGHGQHGVHG | AMP | AMP | APD6 |
| LLPIVGNLLKSLL | AMP | AMP | APD6 |
| IKITTMLAKLGKVLAHV | AMP | AMP | APD6 |
| GLKKLLGKLLKKLGKLLLK | AMP | AMP | APD6 |
| GRPNPVNTKPTPYPRL | AMP | AMP | APD6 |
| RRWCFRVCYKGFCYRKCR | AMP | AMP | APD6 |
| GSKKPVPIIYCNRRTGKCQRM | AMP | AMP | APD6 |
| GVLSNVIGYLKKLGTGALNAVLKQ | AMP | AMP | APD6 |
| KWRRWVRWI | AMP | AMP | APD6 |
| RGGRLCYCRGWICFCVGR | AMP | AMP | APD6 |
| RWRRWR | AMP | AMP | CAMPR4 |
| WRWWRW | AMP | AMP | CAMPR4 |
| LKKLLKKLKKLL | AMP | AMP | CAMPR4 |
| GLFDIVKKIAGHIASSI | AMP | AMP | CAMPR4 |
| FLGGLIKIVPAMICAVTKK | AMP | AMP | CAMPR4 |
| FLGALFKVASKVLPSVFCAITKKC | AMP | AMP | CAMPR4 |
| GLFDIVKKVVGAFGSL | AMP | AMP | CAMPR4 |
| QRPYTQPLIYYPPPPTPPRIYRA | AMP | AMP | CAMPR4 |
| FLSLIPHAINAVSAIAKHFG | AMP | AMP | CAMPR4 |
| RGGRLCYCRRRFCICV | AMP | AMP | CAMPR4 |
| GILDAIKAIAKAAG | AMP | AMP | DRAMP |
| FFFLSRIFGK | AMP | AMP | DRAMP |
| LKLKSIVSWAKKVL | AMP | AMP | DRAMP |
| NILNTIINLAKKIL | AMP | AMP | DRAMP |
| LVRGCWTKSYPPKPCFVR | AMP | AMP | DRAMP |
| GLFDVIKKVASVIGLASP | AMP | AMP | DRAMP |
| GGAGHVPEYFVGIGTPISFYG | AMP | AMP | DRAMP |
| GNGVLKTISHECNMNTWQFLFTCC | AMP | AMP | DRAMP |
| GLKEIFKAGLGSLVKGIAAHVAS | AMP | AMP | DRAMP |
| FLSHIAGFLSNLFGK | AMP | AMP | DRAMP |
| AALRGCWTKSIPPKPCPGKR | AMP | AMP | dbAMP |
| AAPRGGKGFFCKLFKDC | AMP | AMP | dbAMP |
| AKKPVAKKAAGGVKKPK | AMP | AMP | dbAMP |
| AFRKQLKW | AMP | AMP | dbAMP |
| AGFAAQAAASLAPVAAQQL | AMP | AMP | dbAMP |
| AKRHHGYKRKFH | AMP | AMP | dbAMP |
| AGFVLKGYTKTSQ | AMP | non-AMP | dbAMP |
| AILTTLANWARKFL | AMP | AMP | dbAMP |
| AIPWSIWWRLLFKG | AMP | AMP | dbAMP |
| APDRPRKFCGILG | AMP | AMP | dbAMP |
| MRWQEMGYIFYPRKLR | non-AMP | non-AMP | Uniport |
| DTLKSEKNADFKDLY | non-AMP | non-AMP | Uniport |
| MEMALMVAQTRKGKSVV | non-AMP | non-AMP | Uniport |
| DVVSPPVCGN | non-AMP | non-AMP | Uniport |
| GFRSPCPPFC | non-AMP | non-AMP | Uniport |
| YIELAVVADHGIFTKYNSNLNTIR | non-AMP | non-AMP | Uniport |
| VVGGDCIPQVPFLAFLYSEYFC | non-AMP | non-AMP | Uniport |
| GCCSLPPCAANNPDYC | non-AMP | non-AMP | Uniport |
| KRNGCCNCSSKWCRDHSRCCGR | non-AMP | non-AMP | Uniport |
| SLGGKPDLRPCHPPCHYIPRPKPR | non-AMP | non-AMP | Uniport |
| MAAYLDPTGQY | non-AMP | non-AMP | Uniport |
| AIGPVDEV | non-AMP | non-AMP | Uniport |
| CCGVPNAACPPCVCNKTCG | non-AMP | non-AMP | Uniport |
| VIGGDECNINEHRFL | non-AMP | non-AMP | Uniport |
| CCSQDCRVCIPCCPY | non-AMP | non-AMP | Uniport |
| MGGKWSKSS | non-AMP | non-AMP | Uniport |
| LPASVDWRKEGAVLPIRHQGQCG | non-AMP | non-AMP | Uniport |
| GPMEDPLEIIRI | non-AMP | non-AMP | Uniport |
| VIGGDECNINEHRSLVAIFDS | non-AMP | non-AMP | Uniport |
| VVGGDECNINE | non-AMP | non-AMP | Uniport |
| SWFKHKSK | AMP | AMP | DBAASP |
| KLIIKWLIKILK | AMP | AMP | DBAASP |
| RVKRVWPLVIRTVIAGYNLYRAIKKK | AMP | AMP | DBAASP |
| RKRIHIGPGRAFYTT | AMP | AMP | DBAASP |
| GLRKRLRKFRNKIKEKLKKI | AMP | AMP | DBAASP |
| GIKEFKRIVQRIKDFLRNLV | AMP | AMP | DBAASP |
| FPWWWPF | AMP | AMP | DBAASP |
| KWKSFIKKLTSAAKKVVTTAKPLALIS | AMP | AMP | DBAASP |
| YVLWKRKRFIFI | AMP | AMP | DBAASP |
| GWKRKRFG | AMP | AMP | DBAASP |
| GIGAVLKVLTTGLPALISWIKRKRQQ | AMP | AMP | StarPep |
| GIGKFLHSAKKFGKAFVGEIMNS | AMP | AMP | StarPep |
| ILPWKWPWWPWRR | AMP | AMP | StarPep |
| FFHHIFRGIVHVGKTIHRLVTG | AMP | AMP | StarPep |
| FLPLIGRVLSGIL | AMP | AMP | StarPep |
| FWGALAKGALKLIPSLFSSFSKKD | AMP | AMP | StarPep |
| GLFDVIKKVASVIGGL | AMP | AMP | StarPep |
| GLFGVLAKVAAHVVPAIAEHF | AMP | AMP | StarPep |
| GLLKRIKTLL | AMP | AMP | StarPep |
| GLFDIIKKIAESF | AMP | AMP | StarPep |
| FFNDGKGTIYYGIKKYFRIYF | AMP | AMP | AMPSphere |
| LKYLLRRLVVPP | AMP | AMP | AMPSphere |
| LKGELIKRLFALYTVGYYL | AMP | AMP | AMPSphere |
| IFAAVGGGMIIACGAFVWFILKR | AMP | AMP | AMPSphere |
| LKGELIKRLFALYTVGYYL | AMP | AMP | AMPSphere |
| IPKSFQNLYRYLLYETKV | AMP | AMP | AMPSphere |
| ACGGKKSGGKKGKGGKGSK | AMP | AMP | AMPSphere |
| FITVKYLTKMKLFCEKIWWNEK | AMP | AMP | AMPSphere |
| SIVEIRLKLRHIKGVYRIAGI | AMP | AMP | AMPSphere |
| AGGGVVLIGLQLIPLLSGLFS | AMP | AMP | AMPSphere |
| KLKGLLGGKKKLK | AMP | AMP | ^[1]^ |
| AKKFKIWCAIKRLLA | AMP | AMP | ^[2]^ |
| AKLKPKILIWLLKKLKRII | AMP | AMP | ^[3]^ |
| GLWKIKLAFGKMFAK | AMP | AMP | ^[4]^ |
| FLGMIPGLIGGLIKAFK | AMP | AMP | ^[5]^ |
| AKLLKALLKLLKKLA | AMP | AMP | ^[6]^ |
| YLRLIRYMAKMI | AMP | AMP | ^[7]^ |
| DTFGRCRRWWAALGACRR | AMP | AMP | ^[8]^ |
| HIMPIQAIKIPKPKPKPKIKI | AMP | AMP | ^[9]^ |
| GLFKKLRRKIKKGFKKIFKRL | AMP | AMP | ^[10]^ |

**Table S3: Statistical analysis of external validation results for the PreAMP model.**

|  |  | Net charge | Isoelectric point | Hydrophobicity | Amphiphilicity | Alpha helix | Beta sheet |
| --- | --- | --- | --- | --- | --- | --- | --- |
| Modlamp  Biopython | Mean | 4.2626 | 11.9796 | 0.0763 | 1.5686 | 0.3663 | 0.4316 |
|  | SD | 2.2997 | 1.4088 | 1.0230 | 0.4708 | 0.2100 | 0.1425 |
|  | Minimum | 0.0280 | 7.6406 | -3.3000 | 0.5588 | 0.0000 | 0.0000 |
|  | 1/4 value | 2.9830 | 10.7919 | -0.6733 | 1.1800 | 0.2286 | 0.3333 |
|  | 1/2 value | 3.9885 | 12.1620 | 0.3000 | 1.6094 | 0.3481 | 0.4583 |
|  | 3/4 value | 5.0290 | 13.2344 | 0.8429 | 1.8735 | 0.5000 | 0.5317 |
|  | Maximum | 11.9850 | 13.8633 | 1.8077 | 2.5345 | 1.0000 | 0.7143 |
| PreAMP | Mean | 4.1182 | 12.0346 | 0.0728 | 1.5607 | 0.3605 | 0.4289 |
|  | SD | 2.2215 | 1.4605 | 1.0454 | 0.4741 | 0.2118 | 0.1408 |
|  | Minimum | -0.1421 | 7.9244 | -3.2520 | 0.6197 | 0.0212 | 0.0112 |
|  | 1/4 value | 2.7476 | 11.0461 | -0.6503 | 1.1514 | 0.2070 | 0.3320 |
|  | 1/2 value | 3.9326 | 12.4321 | 0.2693 | 1.5497 | 0.3344 | 0.4554 |
|  | 3/4 value | 5.0456 | 13.3102 | 0.7836 | 1.8887 | 0.4819 | 0.5342 |
|  | Maximum | 11.4305 | 14.0433 | 1.9073 | 2.5434 | 1.0000 | 0.7381 |

**Table S4: External validation results for the Modlamp and Biopython tools.**

| Sequences | Net charge | Isoelectric point | Hydrophobicity | Amphiphilicity | Alpha helix | Beta sheet |
| --- | --- | --- | --- | --- | --- | --- |
| HGVSGHGQHGVHG | 1.1570 | 12.2500 | -0.8231 | 1.0910 | 0.0000 | 0.1538 |
| LLPIVGNLLKSLL | 1.9890 | 12.8438 | 1.6385 | 2.1539 | 0.5385 | 0.6154 |
| IKITTMLAKLGKVLAHV | 4.0300 | 13.0781 | 1.0353 | 1.6648 | 0.5294 | 0.5294 |
| GLKKLLGKLLKKLGKLLLK | 7.9860 | 13.2656 | 0.3000 | 2.2714 | 0.8421 | 0.4737 |
| GRPNPVNTKPTPYPRL | 3.9870 | 12.1367 | -1.4375 | 0.8889 | 0.1250 | 0.3125 |
| RRWCFRVCYKGFCYRKCR | 7.3690 | 10.7961 | -0.8000 | 1.6036 | 0.1111 | 0.3333 |
| GSKKPVPIIYCNRRTGKCQRM | 6.6780 | 11.4150 | -0.9000 | 1.1883 | 0.1905 | 0.2381 |
| GVLSNVIGYLKKLGTGALNAVLKQ | 3.9860 | 11.0527 | 0.5458 | 1.7788 | 0.4167 | 0.4583 |
| KWRRWVRWI | 4.9890 | 13.8008 | -1.2667 | 1.8079 | 0.1111 | 0.5556 |
| RGGRLCYCRGWICFCVGR | 4.3720 | 9.8057 | 0.1944 | 1.0200 | 0.0556 | 0.3333 |
| RWRRWR | 4.9900 | 13.8633 | -3.3000 | 1.0020 | 0.0000 | 0.3333 |
| WRWWRW | 2.9900 | 13.7070 | -2.1000 | 1.3993 | 0.0000 | 0.6667 |
| LKKLLKKLKKLL | 6.9860 | 13.2344 | -0.0500 | 2.5345 | 1.0000 | 0.5000 |
| GLFDIVKKIAGHIASSI | 2.0310 | 10.7090 | 0.9118 | 2.1152 | 0.2941 | 0.4118 |
| FLGGLIKIVPAMICAVTKK | 3.8340 | 10.9878 | 1.3421 | 1.4557 | 0.4211 | 0.4737 |
| FLGALFKVASKVLPSVFCAITKKC | 4.6800 | 10.6924 | 1.1417 | 1.5883 | 0.4167 | 0.4583 |
| GLFDIVKKVVGAFGSL | 1.9890 | 10.7090 | 1.1750 | 1.9863 | 0.3125 | 0.5000 |
| QRPYTQPLIYYPPPPTPPRIYRA | 3.9820 | 10.5635 | -1.1000 | 1.1041 | 0.0870 | 0.3913 |
| FLSLIPHAINAVSAIAKHFG | 2.0730 | 12.8438 | 1.0350 | 1.8231 | 0.3500 | 0.4000 |
| RGGRLCYCRRRFCICV | 5.3720 | 10.7715 | 0.0438 | 1.1880 | 0.0625 | 0.3125 |
| GILDAIKAIAKAAG | 1.9890 | 10.7090 | 1.0143 | 2.0134 | 0.5714 | 0.2857 |
| FFFLSRIFGK | 2.9890 | 13.5586 | 0.9900 | 1.7320 | 0.2000 | 0.6000 |
| LKLKSIVSWAKKVL | 4.9870 | 13.1406 | 0.5714 | 1.6553 | 0.5714 | 0.5000 |
| NILNTIINLAKKIL | 2.9890 | 13.0000 | 0.8714 | 2.2703 | 0.4286 | 0.5714 |
| LVRGCWTKSYPPKPCFVR | 4.6790 | 10.7793 | -0.3167 | 1.0144 | 0.1667 | 0.3889 |
| GLFDVIKKVASVIGLASP | 1.9890 | 10.7090 | 1.1278 | 1.8808 | 0.3333 | 0.4444 |
| GGAGHVPEYFVGIGTPISFYG | 0.0280 | 7.6406 | 0.4000 | 1.1429 | 0.0952 | 0.4286 |
| GNGVLKTISHECNMNTWQFLFTCC | 0.5700 | 7.8306 | 0.0875 | 1.4125 | 0.2083 | 0.4167 |
| GLKEIFKAGLGSLVKGIAAHVAS | 3.0300 | 10.9868 | 0.7478 | 1.7044 | 0.4783 | 0.3478 |
| FLSHIAGFLSNLFGK | 2.0310 | 12.8438 | 0.8733 | 1.7741 | 0.3333 | 0.4667 |
| AALRGCWTKSIPPKPCPGKR | 5.6800 | 11.4956 | -0.6700 | 1.2281 | 0.3000 | 0.2000 |
| AAPRGGKGFFCKLFKDC | 3.6800 | 10.4165 | -0.1000 | 1.5412 | 0.3529 | 0.2353 |
| AKKPVAKKAAGGVKKPK | 7.9860 | 13.2656 | -0.9235 | 1.6413 | 0.6471 | 0.1176 |
| AFRKQLKW | 3.9890 | 13.5664 | -1.0375 | 1.6266 | 0.5000 | 0.3750 |
| AGFAAQAAASLAPVAAQQL | 0.9900 | 12.2500 | 0.9211 | 1.1232 | 0.5789 | 0.2105 |
| AKRHHGYKRKFH | 6.1110 | 12.1846 | -2.2833 | 0.9478 | 0.3333 | 0.1667 |
| AILTTLANWARKFL | 2.9890 | 13.5586 | 0.7071 | 1.7244 | 0.5000 | 0.5714 |
| AIPWSIWWRLLFKG | 2.9890 | 13.5586 | 0.5214 | 1.4426 | 0.2857 | 0.5714 |
| APDRPRKFCGILG | 2.8350 | 11.2217 | -0.3846 | 1.2181 | 0.2308 | 0.2308 |
| MRWQEMGYIFYPRKLR | 3.9860 | 11.2378 | -0.9375 | 1.1552 | 0.3125 | 0.3750 |
| DTLKSEKNADFKDLY | -0.0130 | 6.8750 | -1.3200 | 1.1838 | 0.4667 | 0.3333 |
| MEMALMVAQTRKGKSVV | 2.9890 | 11.5723 | 0.2647 | 0.9065 | 0.5294 | 0.2941 |
| DVVSPPVCGN | -0.1640 | 5.9063 | 0.3700 | 1.4977 | 0.0000 | 0.3000 |
| GFRSPCPPFC | 1.6820 | 8.9463 | 0.0100 | 1.1241 | 0.0000 | 0.2000 |
| YIELAVVADHGIFTKYNSNLNTIR | 1.0280 | 9.4751 | 0.0667 | 1.1345 | 0.2500 | 0.5000 |
| VVGGDCIPQVPFLAFLYSEYFC | -1.3210 | 3.9297 | 1.0000 | 1.2000 | 0.1818 | 0.5000 |
| GCCSLPPCAANNPDYC | -0.6280 | 5.6250 | -0.0250 | 0.9547 | 0.1875 | 0.1250 |
| KRNGCCNCSSKWCRDHSRCCGR | 5.1070 | 9.2075 | -1.3000 | 1.2174 | 0.0909 | 0.0455 |
| SLGGKPDLRPCHPPCHYIPRPKPR | 4.7620 | 10.7544 | -1.1750 | 1.2721 | 0.1667 | 0.1667 |
| MAAYLDPTGQY | -0.0140 | 6.5000 | -0.2727 | 1.0237 | 0.3636 | 0.3636 |
| AIGPVDEV | -1.0100 | 3.9297 | 0.7125 | 1.8755 | 0.2500 | 0.3750 |
| CCGVPNAACPPCVCNKTCG | 1.0650 | 7.8296 | 0.5158 | 1.3208 | 0.1579 | 0.1579 |
| VIGGDECNINEHRFL | -1.1210 | 5.3027 | -0.2467 | 0.9462 | 0.2000 | 0.3333 |
| CCSQDCRVCIPCCPY | 0.0640 | 7.4341 | 0.4600 | 1.2080 | 0.0000 | 0.2000 |
| MGGKWSKSS | 2.9890 | 13.0000 | -1.1111 | 0.7582 | 0.3333 | 0.1111 |
| LPASVDWRKEGAVLPIRHQGQCG | 1.8780 | 10.0195 | -0.4174 | 0.8039 | 0.2609 | 0.2609 |
| GPMEDPLEIIRI | -1.0090 | 4.3223 | 0.0500 | 1.6571 | 0.3333 | 0.3333 |
| VIGGDECNINEHRSLVAIFDS | -2.1210 | 4.4209 | 0.0810 | 0.9860 | 0.1905 | 0.3333 |
| VVGGDECNINE | -2.1630 | 3.6875 | -0.2636 | 0.8557 | 0.1818 | 0.2727 |
| SWFKHKSK | 4.0300 | 13.0781 | -1.8250 | 1.0650 | 0.3750 | 0.2500 |
| KLIIKWLIKILK | 4.9870 | 13.1406 | 1.0750 | 2.1924 | 0.5833 | 0.6667 |
| RVKRVWPLVIRTVIAGYNLYRAIKKK | 8.9830 | 12.1619 | -0.0692 | 1.7843 | 0.3077 | 0.5000 |
| RKRIHIGPGRAFYTT | 5.0290 | 12.4146 | -0.8067 | 1.1239 | 0.1333 | 0.4000 |
| GLRKRLRKFRNKIKEKLKKI | 10.9860 | 12.6211 | -1.4750 | 1.9541 | 0.5500 | 0.3000 |
| GIKEFKRIVQRIKDFLRNLV | 4.9890 | 11.9043 | -0.2250 | 2.5242 | 0.3000 | 0.4500 |
| FPWWWPF | 0.9900 | 12.2500 | -0.0429 | 0.5588 | 0.0000 | 0.7143 |
| KWKSFIKKLTSAAKKVVTTAKPLALIS | 7.9860 | 13.2656 | 0.1667 | 1.6152 | 0.5185 | 0.4444 |
| YVLWKRKRFIFI | 4.9870 | 12.1621 | 0.3000 | 0.9885 | 0.2500 | 0.6667 |
| GWKRKRFG | 4.9890 | 13.7148 | -1.9625 | 0.6356 | 0.2500 | 0.2500 |
| GIGAVLKVLTTGLPALISWIKRKRQQ | 5.9880 | 13.7188 | 0.2731 | 1.4644 | 0.3462 | 0.4615 |
| GIGKFLHSAKKFGKAFVGEIMNS | 4.0300 | 11.1567 | 0.0826 | 1.8710 | 0.3913 | 0.3043 |
| ILPWKWPWWPWRR | 3.9890 | 13.7109 | -1.0692 | 0.5873 | 0.1538 | 0.5385 |
| FFHHIFRGIVHVGKTIHRLVTG | 4.1560 | 13.7109 | 0.4545 | 2.2396 | 0.0909 | 0.5455 |
| FLPLIGRVLSGIL | 1.9900 | 13.5508 | 1.8077 | 2.0742 | 0.3077 | 0.6154 |
| FWGALAKGALKLIPSLFSSFSKKD | 3.9880 | 11.1567 | 0.3292 | 1.5373 | 0.4583 | 0.3750 |
| GLFDVIKKVASVIGGL | 1.9890 | 10.7090 | 1.2813 | 2.0909 | 0.3125 | 0.5000 |
| GLFGVLAKVAAHVVPAIAEHF | 1.0730 | 10.0254 | 1.3000 | 1.6680 | 0.4286 | 0.4286 |
| GLLKRIKTLL | 3.9890 | 13.5664 | 0.6300 | 2.2419 | 0.6000 | 0.6000 |
| GLFDIIKKIAESF | 0.9890 | 9.8398 | 0.6692 | 2.4458 | 0.3846 | 0.4615 |
| FFNDGKGTIYYGIKKYFRIYF | 3.9800 | 10.2336 | -0.2667 | 1.2936 | 0.1429 | 0.5714 |
| LKYLLRRLVVPP | 3.9870 | 12.1367 | 0.5167 | 2.0927 | 0.4167 | 0.5833 |
| LKGELIKRLFALYTVGYYL | 2.9830 | 10.1792 | 0.5842 | 1.6309 | 0.4737 | 0.6316 |
| IFAAVGGGMIIACGAFVWFILKR | 2.8350 | 11.4004 | 1.7087 | 1.0299 | 0.3043 | 0.4783 |
| LKGELIKRLFALYTVGYYL | 2.9830 | 10.1792 | 0.5842 | 1.6309 | 0.4737 | 0.6316 |
| IPKSFQNLYRYLLYETKV | 2.9830 | 10.1792 | -0.3833 | 1.7607 | 0.3333 | 0.5556 |
| ACGGKKSGGKKGKGGKGSK | 7.8320 | 11.4526 | -1.4632 | 0.8699 | 0.4211 | 0.0000 |
| FITVKYLTKMKLFCEKIWWNEK | 3.8320 | 10.3018 | -0.1682 | 1.3407 | 0.4545 | 0.5455 |
| SIVEIRLKLRHIKGVYRIAGI | 5.0290 | 11.8838 | 0.4476 | 1.6015 | 0.2857 | 0.4762 |
| AGGGVVLIGLQLIPLLSGLFS | 0.9900 | 12.2500 | 1.7190 | 1.2643 | 0.3333 | 0.5238 |
| KLKGLLGGKKKLK | 6.9860 | 13.2344 | -0.7231 | 1.1477 | 0.7692 | 0.3077 |
| AKKFKIWCAIKRLLA | 5.8340 | 11.7520 | 0.4200 | 1.4120 | 0.6000 | 0.4000 |
| AKLKPKILIWLLKKLKRII | 7.9860 | 13.5977 | 0.4421 | 1.8031 | 0.6316 | 0.5263 |
| GLWKIKLAFGKMFAK | 4.9870 | 13.1406 | 0.3933 | 1.3561 | 0.6000 | 0.4000 |
| FLGMIPGLIGGLIKAFK | 2.9890 | 13.0000 | 1.3647 | 1.7490 | 0.4118 | 0.4706 |
| AKLLKALLKLLKKLA | 5.9870 | 13.1875 | 0.8333 | 2.2723 | 1.0000 | 0.4667 |
| YLRLIRYMAKMI | 3.9850 | 11.3398 | 0.5583 | 2.1106 | 0.5000 | 0.5000 |
| DTFGRCRRWWAALGACRR | 4.6820 | 11.9248 | -0.6833 | 1.1561 | 0.2222 | 0.2778 |
| HIMPIQAIKIPKPKPKPKIKI | 7.0280 | 13.2344 | -0.3524 | 1.1936 | 0.3810 | 0.2857 |
| GLFKKLRRKIKKGFKKIFKRL | 11.9850 | 13.8203 | -0.7952 | 2.2797 | 0.5238 | 0.3810 |

**Table S5: External validation results for the PreAMP model.**

| Sequences | Net charge | Isoelectric point | Hydrophobicity | Amphiphilicity | Alpha helix | Beta sheet |
| --- | --- | --- | --- | --- | --- | --- |
| HGVSGHGQHGVHG | 1.0478 | 11.8420 | -0.9139 | 1.1576 | 0.0212 | 0.1360 |
| LLPIVGNLLKSLL | 2.1991 | 12.8913 | 1.6095 | 2.0974 | 0.5356 | 0.6040 |
| IKITTMLAKLGKVLAHV | 3.7802 | 13.1093 | 1.1955 | 1.5871 | 0.5229 | 0.5292 |
| GLKKLLGKLLKKLGKLLLK | 7.4481 | 13.5483 | 0.2875 | 2.3012 | 0.8320 | 0.4849 |
| GRPNPVNTKPTPYPRL | 3.6770 | 12.0269 | -1.5999 | 0.8377 | 0.1114 | 0.3014 |
| RRWCFRVCYKGFCYRKCR | 7.0990 | 10.7439 | -0.6673 | 1.5580 | 0.1020 | 0.3461 |
| GSKKPVPIIYCNRRTGKCQRM | 6.5731 | 11.6799 | -0.8384 | 1.0818 | 0.1821 | 0.2585 |
| GVLSNVIGYLKKLGTGALNAVLKQ | 3.7762 | 11.7893 | 0.5140 | 1.9068 | 0.3986 | 0.4665 |
| KWRRWVRWI | 5.0447 | 13.8968 | -1.2778 | 1.6848 | 0.1006 | 0.5432 |
| RGGRLCYCRGWICFCVGR | 4.2386 | 10.0548 | 0.2511 | 1.0109 | 0.0550 | 0.3171 |
| RWRRWR | 4.8638 | 14.0433 | -3.2520 | 0.9502 | 0.0075 | 0.3347 |
| WRWWRW | 2.8568 | 13.7250 | -2.0086 | 1.2170 | 0.0130 | 0.6692 |
| LKKLLKKLKKLL | 7.0125 | 13.0916 | -0.0760 | 2.5434 | 0.9652 | 0.4988 |
| GLFDIVKKIAGHIASSI | 1.9508 | 11.0044 | 1.0475 | 2.1425 | 0.2972 | 0.4227 |
| FLGGLIKIVPAMICAVTKK | 3.6828 | 11.5534 | 1.2799 | 1.4967 | 0.3950 | 0.4785 |
| FLGALFKVASKVLPSVFCAITKKC | 4.2513 | 10.9420 | 1.2205 | 1.6213 | 0.4136 | 0.4649 |
| GLFDIVKKVVGAFGSL | 1.8719 | 10.8334 | 1.2227 | 2.0890 | 0.2924 | 0.4975 |
| QRPYTQPLIYYPPPPTPPRIYRA | 4.1724 | 10.7460 | -0.9748 | 1.0855 | 0.0836 | 0.4051 |
| FLSLIPHAINAVSAIAKHFG | 2.2766 | 12.8253 | 1.0881 | 1.8010 | 0.3392 | 0.3995 |
| RGGRLCYCRRRFCICV | 5.2459 | 10.3146 | 0.0447 | 1.0066 | 0.0681 | 0.3080 |
| GILDAIKAIAKAAG | 1.8188 | 10.7268 | 0.9508 | 1.9886 | 0.5817 | 0.2810 |
| FFFLSRIFGK | 2.7659 | 13.6774 | 0.8670 | 1.6606 | 0.2000 | 0.5977 |
| LKLKSIVSWAKKVL | 4.9364 | 13.4191 | 0.6185 | 1.6640 | 0.5727 | 0.4998 |
| NILNTIINLAKKIL | 3.1178 | 12.9292 | 0.8653 | 2.3455 | 0.4161 | 0.5417 |
| LVRGCWTKSYPPKPCFVR | 4.2963 | 11.1254 | -0.1943 | 1.2753 | 0.1577 | 0.3922 |
| GLFDVIKKVASVIGLASP | 2.0152 | 10.9752 | 1.1980 | 1.9498 | 0.3126 | 0.4517 |
| GGAGHVPEYFVGIGTPISFYG | -0.1421 | 7.9244 | 0.4996 | 1.0407 | 0.1053 | 0.4404 |
| GNGVLKTISHECNMNTWQFLFTCC | 0.6515 | 8.1841 | 0.1197 | 1.4650 | 0.2093 | 0.3814 |
| GLKEIFKAGLGSLVKGIAAHVAS | 2.8564 | 11.4810 | 0.7746 | 1.8635 | 0.4759 | 0.3601 |
| FLSHIAGFLSNLFGK | 2.0262 | 12.6839 | 1.0238 | 1.8412 | 0.3066 | 0.4610 |
| AALRGCWTKSIPPKPCPGKR | 5.0483 | 11.6740 | -0.6456 | 1.2119 | 0.2926 | 0.1893 |
| AAPRGGKGFFCKLFKDC | 3.0731 | 9.3628 | 0.0443 | 1.4931 | 0.3240 | 0.2343 |
| AKKPVAKKAAGGVKKPK | 7.9342 | 13.2468 | -0.8876 | 1.5333 | 0.6628 | 0.1375 |
| AFRKQLKW | 4.0766 | 13.7295 | -1.1096 | 1.5888 | 0.5221 | 0.3735 |
| AGFAAQAAASLAPVAAQQL | 1.2053 | 11.7694 | 1.1906 | 1.0249 | 0.6124 | 0.1898 |
| AKRHHGYKRKFH | 5.9509 | 12.6286 | -2.4468 | 1.1331 | 0.3247 | 0.1643 |
| AILTTLANWARKFL | 3.2148 | 13.2851 | 0.8106 | 1.7965 | 0.4907 | 0.5833 |
| AIPWSIWWRLLFKG | 2.9778 | 13.3746 | 0.5942 | 1.4651 | 0.2760 | 0.6068 |
| APDRPRKFCGILG | 2.8651 | 10.9189 | -0.6487 | 1.0469 | 0.1882 | 0.1745 |
| MRWQEMGYIFYPRKLR | 3.6874 | 10.3901 | -0.9093 | 1.1257 | 0.2825 | 0.3854 |
| DTLKSEKNADFKDLY | -0.6324 | 6.4224 | -1.2299 | 1.4113 | 0.4366 | 0.3360 |
| MEMALMVAQTRKGKSVV | 3.3112 | 11.2838 | 0.3493 | 1.0008 | 0.4959 | 0.3127 |
| DVVSPPVCGN | -0.3980 | 7.9187 | 0.4285 | 1.5252 | 0.0206 | 0.3095 |
| GFRSPCPPFC | 1.6147 | 9.4008 | -0.0148 | 1.1493 | 0.0061 | 0.1981 |
| YIELAVVADHGIFTKYNSNLNTIR | 1.1550 | 8.7037 | 0.1092 | 1.2336 | 0.2390 | 0.4977 |
| VVGGDCIPQVPFLAFLYSEYFC | -0.9896 | 5.7651 | 0.8552 | 1.2470 | 0.1666 | 0.4927 |
| GCCSLPPCAANNPDYC | -0.9882 | 5.2068 | -0.1678 | 1.0389 | 0.1935 | 0.1386 |
| KRNGCCNCSSKWCRDHSRCCGR | 4.3565 | 9.6473 | -1.1266 | 1.2431 | 0.1187 | 0.0756 |
| SLGGKPDLRPCHPPCHYIPRPKPR | 4.0554 | 10.5062 | -1.1517 | 1.0872 | 0.1531 | 0.1590 |
| MAAYLDPTGQY | -0.6643 | 6.6178 | -0.5087 | 0.8368 | 0.3227 | 0.3587 |
| AIGPVDEV | -1.7268 | 5.7127 | 0.4357 | 1.5233 | 0.2596 | 0.3938 |
| CCGVPNAACPPCVCNKTCG | 0.9904 | 7.1004 | 0.5002 | 1.3567 | 0.1488 | 0.1643 |
| VIGGDECNINEHRFL | -1.4322 | 6.2139 | -0.2924 | 1.2319 | 0.1886 | 0.3215 |
| CCSQDCRVCIPCCPY | 0.2439 | 5.5914 | 0.2830 | 1.1641 | 0.0639 | 0.2381 |
| MGGKWSKSS | 2.7614 | 12.7651 | -1.1913 | 0.6314 | 0.3188 | 0.1006 |
| LPASVDWRKEGAVLPIRHQGQCG | 1.5435 | 9.6780 | -0.3480 | 0.8755 | 0.2524 | 0.2555 |
| GPMEDPLEIIRI | -0.9931 | 6.5161 | -0.0823 | 1.5430 | 0.3233 | 0.3275 |
| VIGGDECNINEHRSLVAIFDS | -1.9145 | 5.8708 | -0.0187 | 1.1066 | 0.1829 | 0.3202 |
| VVGGDECNINE | -2.6445 | 3.3581 | -0.4887 | 1.2182 | 0.2161 | 0.2458 |
| SWFKHKSK | 4.4368 | 13.3828 | -1.9885 | 0.7914 | 0.3800 | 0.2626 |
| KLIIKWLIKILK | 4.4233 | 13.5475 | 0.9621 | 2.3192 | 0.5501 | 0.6599 |
| RVKRVWPLVIRTVIAGYNLYRAIKKK | 8.3603 | 12.4551 | -0.0600 | 1.8827 | 0.2994 | 0.4891 |
| RKRIHIGPGRAFYTT | 4.7269 | 12.4811 | -0.8195 | 1.0309 | 0.1307 | 0.4268 |
| GLRKRLRKFRNKIKEKLKKI | 10.9436 | 12.9034 | -1.3518 | 1.9978 | 0.5457 | 0.3134 |
| GIKEFKRIVQRIKDFLRNLV | 4.8991 | 11.9048 | -0.2056 | 2.5338 | 0.3082 | 0.4387 |
| FPWWWPF | 1.0460 | 12.6243 | -0.1122 | 0.7107 | 0.0163 | 0.7381 |
| KWKSFIKKLTSAAKKVVTTAKPLALIS | 7.6739 | 13.1990 | 0.0877 | 1.6501 | 0.5091 | 0.4385 |
| YVLWKRKRFIFI | 4.8203 | 12.7891 | 0.1744 | 1.1140 | 0.2595 | 0.6144 |
| GWKRKRFG | 5.2442 | 13.8290 | -2.2296 | 0.9564 | 0.2666 | 0.2389 |
| GIGAVLKVLTTGLPALISWIKRKRQQ | 5.8432 | 13.3000 | 0.4193 | 1.4106 | 0.3691 | 0.4572 |
| GIGKFLHSAKKFGKAFVGEIMNS | 3.8242 | 11.4501 | 0.1712 | 1.8639 | 0.3771 | 0.3047 |
| ILPWKWPWWPWRR | 3.9183 | 13.6208 | -1.2649 | 0.6197 | 0.1221 | 0.5548 |
| FFHHIFRGIVHVGKTIHRLVTG | 4.1718 | 13.3874 | 0.5048 | 2.2220 | 0.0974 | 0.5379 |
| FLPLIGRVLSGIL | 2.1262 | 13.5170 | 1.7973 | 1.9545 | 0.2958 | 0.6180 |
| FWGALAKGALKLIPSLFSSFSKKD | 3.9025 | 12.1358 | 0.3512 | 1.5231 | 0.4790 | 0.3540 |
| GLFDVIKKVASVIGGL | 1.9960 | 11.0601 | 1.3003 | 2.1804 | 0.3000 | 0.5010 |
| GLFGVLAKVAAHVVPAIAEHF | 1.1159 | 10.2151 | 1.3383 | 1.6275 | 0.4396 | 0.4252 |
| GLLKRIKTLL | 4.0285 | 13.6323 | 0.6625 | 2.2466 | 0.5980 | 0.6018 |
| GLFDIIKKIAESF | 0.8575 | 8.8000 | 0.7036 | 2.4259 | 0.3619 | 0.4536 |
| FFNDGKGTIYYGIKKYFRIYF | 3.9470 | 9.8912 | -0.2788 | 1.2034 | 0.1778 | 0.5329 |
| LKYLLRRLVVPP | 3.9744 | 12.4091 | 0.5517 | 2.1808 | 0.4223 | 0.5720 |
| LKGELIKRLFALYTVGYYL | 2.7476 | 9.6469 | 0.5012 | 1.6992 | 0.4674 | 0.6187 |
| IFAAVGGGMIIACGAFVWFILKR | 3.0713 | 11.2289 | 1.6321 | 0.9919 | 0.2947 | 0.4807 |
| LKGELIKRLFALYTVGYYL | 2.7476 | 9.6469 | 0.5012 | 1.6992 | 0.4674 | 0.6187 |
| IPKSFQNLYRYLLYETKV | 2.7669 | 9.4828 | -0.3106 | 1.8618 | 0.3126 | 0.5621 |
| ACGGKKSGGKKGKGGKGSK | 6.8935 | 12.5292 | -1.5516 | 0.8282 | 0.4220 | 0.0112 |
| FITVKYLTKMKLFCEKIWWNEK | 3.3610 | 9.4944 | -0.0106 | 1.5415 | 0.4498 | 0.5380 |
| SIVEIRLKLRHIKGVYRIAGI | 5.0279 | 11.8011 | 0.5161 | 1.5311 | 0.2999 | 0.4921 |
| AGGGVVLIGLQLIPLLSGLFS | 1.2573 | 11.9500 | 1.9073 | 1.1820 | 0.3297 | 0.5427 |
| KLKGLLGGKKKLK | 7.1101 | 13.4223 | -0.7862 | 1.3058 | 0.8052 | 0.3240 |
| AKKFKIWCAIKRLLA | 5.5346 | 12.6987 | 0.4798 | 1.4460 | 0.6064 | 0.4018 |
| AKLKPKILIWLLKKLKRII | 7.9723 | 13.5821 | 0.3906 | 1.7693 | 0.6383 | 0.5118 |
| GLWKIKLAFGKMFAK | 4.8200 | 13.3407 | 0.4953 | 1.3554 | 0.6292 | 0.4008 |
| FLGMIPGLIGGLIKAFK | 3.0861 | 13.3704 | 1.2640 | 1.8294 | 0.4172 | 0.4632 |
| AKLLKALLKLLKKLA | 5.8697 | 13.3907 | 0.7642 | 2.2541 | 1.0000 | 0.4742 |
| YLRLIRYMAKMI | 4.1623 | 11.5419 | 0.5695 | 1.9765 | 0.5127 | 0.4951 |
| DTFGRCRRWWAALGACRR | 4.7097 | 11.6888 | -0.7439 | 1.3149 | 0.1731 | 0.2607 |
| HIMPIQAIKIPKPKPKPKIKI | 6.7294 | 13.0987 | -0.2751 | 1.0574 | 0.4067 | 0.2941 |
| GLFKKLRRKIKKGFKKIFKRL | 11.4305 | 13.4925 | -0.6549 | 2.3100 | 0.5256 | 0.3861 |

**Table S6: Parameter selection for temperature sampling and top-k sampling based on the ClaAMP and PreAMP models.**

| Temperature | 0.8 | | | 1.0 | | | 1.2 | | |
| --- | --- | --- | --- | --- | --- | --- | --- | --- | --- |
| Top-k | 1 | 3 | 5 | 1 | 3 | 5 | 1 | 3 | 5 |
| Accuracy | 0.9045 | 0.9067 | 0.8916 | 0.8937 | 0.9013 | 0.8820 | 0.9013 | 0.9109 | 0.8734 |
| Precision | 1.0000 | 1.0000 | 1.0000 | 1.0000 | 1.0000 | 1.0000 | 1.0000 | 1.0000 | 1.0000 |
| Recall | 0.9045 | 0.9067 | 0.8916 | 0.8937 | 0.9013 | 0.8820 | 0.9013 | 0.9109 | 0.8734 |
| F1 score | 0.9499 | 0.9510 | 0.9427 | 0.9433 | 0.9481 | 0.9373 | 0.9481 | 0.9534 | 0.9324 |
| Net charge MSE | 10.9038 | 7.2579 | 4.7423 | 11.9901 | 6.4420 | 4.6448 | 11.8392 | 6.1082 | 4.7870 |
| Isoelectric point MSE | 1.5725 | 1.4324 | 1.4248 | 1.6463 | 1.3450 | 1.2060 | 1.5843 | 1.5291 | 1.3130 |
| Hydrophobicity MSE | 1.9607 | 0.9734 | 0.7244 | 1.9928 | 0.7563 | 0.6611 | 1.9834 | 0.7676 | 0.6742 |
| Amphiphilicity MSE | 0.5319 | 0.3397 | 0.3148 | 0.5583 | 0.3130 | 0.3077 | 0.5547 | 0.3327 | 0.3258 |
| Alpha helix MSE | 0.0436 | 0.0271 | 0.0160 | 0.0425 | 0.0242 | 0.0153 | 0.0426 | 0.0219 | 0.0154 |
| Beta sheet MSE | 0.0379 | 0.0204 | 0.0139 | 0.0383 | 0.0168 | 0.0130 | 0.0388 | 0.0171 | 0.0165 |

**Table S7: Performance evaluation of GenMTAMP and other generative models on the ClaAMP model.**

| Generative model | Generated sample | Predicted as AMP | Predicted as non-AMP | Predicted AMP rate |
| --- | --- | --- | --- | --- |
| PepVAE^[11]^ | 143 | 129 | 14 | 90.21% |
| KPPepGen^[12]^ | 143 | 135 | 8 | 94.41% |
| AMP-Designer^[13]^ | 143 | 139 | 4 | 97.20% |
| HydrAMP^[14]^ | 143 | 138 | 5 | 96.50% |
| GenMTAMP | 143 | 142 | 1 | 99.30% |

**Table S8: Performance evaluation of GenMTAMP and other generative models on the PreAMP model.**

| Generative model | Net charge* | Isoelectric point* | Hydrophobicity* | Amphiphilicity* | Alpha helix* | Beta sheet* |
| --- | --- | --- | --- | --- | --- | --- |
| PepVAE^[11]^ | 4.231 | 10.201 | 0.032 | 1.131 | 0.277 | 0.512 |
| KPPepGen^[12]^ | 4.732 | 11.043 | 0.052 | 1.223 | 0.338 | 0.477 |
| AMP-Designer^[13]^ | 4.787 | 12.217 | 0.046 | 1.301 | 0.371 | 0.431 |
| HydrAMP^[14]^ | 5.534 | 11.390 | 0.045 | 1.353 | 0.368 | 0.450 |
| GenMTAMP | 5.863 | 12.532 | 0.060 | 1.428 | 0.394 | 0.427 |

**Note:** * represents the average of all samples predicted as AMP.

**Table S9: True PCSS descriptors during MTAMP model generation.**

| No. | Net charge | Isoelectric point | Hydrophobicity | Amphiphilicity | Alpha helix | Beta sheet |
| --- | --- | --- | --- | --- | --- | --- |
| 1 | 2.989 | 11.572 | -0.378 | 1.644 | 0.278 | 0.333 |
| 2 | 3.030 | 10.987 | 0.550 | 1.585 | 0.458 | 0.500 |
| 3 | 4.987 | 13.141 | 0.162 | 1.642 | 0.308 | 0.462 |
| 4 | 3.990 | 12.575 | -0.288 | 1.813 | 0.250 | 0.375 |
| 5 | 4.986 | 11.700 | -0.050 | 1.480 | 0.375 | 0.500 |
| 6 | 4.987 | 13.141 | 0.192 | 1.389 | 0.400 | 0.320 |
| 7 | 5.875 | 11.752 | 0.211 | 1.683 | 0.333 | 0.500 |
| 8 | 5.835 | 12.423 | -0.227 | 1.541 | 0.400 | 0.333 |
| 9 | 5.835 | 12.583 | -0.756 | 1.440 | 0.222 | 0.333 |
| 10 | 5.028 | 11.700 | 0.017 | 1.216 | 0.222 | 0.500 |
| 11 | 5.679 | 11.688 | -0.463 | 1.447 | 0.211 | 0.421 |
| 12 | 5.836 | 12.700 | -0.813 | 1.664 | 0.200 | 0.333 |
| 13 | 5.835 | 12.423 | 0.187 | 1.322 | 0.400 | 0.400 |
| 14 | 4.988 | 12.577 | -0.473 | 1.238 | 0.267 | 0.400 |
| 15 | 4.985 | 11.200 | 0.064 | 1.649 | 0.480 | 0.320 |
| 16 | 4.989 | 12.423 | -0.400 | 1.656 | 0.300 | 0.500 |
| 17 | 5.830 | 11.025 | 0.204 | 1.460 | 0.304 | 0.391 |
| 18 | 5.028 | 11.700 | 0.318 | 1.505 | 0.409 | 0.455 |
| 19 | 5.987 | 12.426 | -0.150 | 1.317 | 0.292 | 0.417 |
| 20 | 5.986 | 12.185 | 0.057 | 1.737 | 0.381 | 0.476 |
| 21 | 5.986 | 11.587 | -0.386 | 1.728 | 0.364 | 0.455 |
| 22 | 4.072 | 13.078 | 0.052 | 1.716 | 0.259 | 0.407 |
| 23 | 3.918 | 10.947 | 0.343 | 1.297 | 0.357 | 0.464 |
| 24 | 3.988 | 11.676 | 0.395 | 1.603 | 0.421 | 0.368 |
| 25 | 4.681 | 10.913 | 0.258 | 1.365 | 0.333 | 0.375 |
| 26 | 5.681 | 12.115 | 0.094 | 1.600 | 0.278 | 0.444 |
| 27 | 3.031 | 10.946 | -0.700 | 1.164 | 0.250 | 0.400 |
| 28 | 3.986 | 11.009 | 0.295 | 1.388 | 0.450 | 0.450 |
| 29 | 4.030 | 13.078 | 0.300 | 1.445 | 0.294 | 0.412 |
| 30 | 4.680 | 10.913 | -0.533 | 1.320 | 0.200 | 0.333 |
| 31 | 4.985 | 10.906 | 0.204 | 1.828 | 0.370 | 0.407 |
| 32 | 4.987 | 13.141 | 0.432 | 1.725 | 0.464 | 0.429 |
| 33 | 5.029 | 12.577 | -0.376 | 1.440 | 0.190 | 0.333 |
| 34 | 5.986 | 12.185 | -0.048 | 1.228 | 0.238 | 0.429 |
| 35 | 5.987 | 11.374 | 0.520 | 1.823 | 0.467 | 0.500 |
| 36 | 5.987 | 11.374 | 0.273 | 1.833 | 0.462 | 0.500 |
| 37 | 5.987 | 11.374 | -0.832 | 1.755 | 0.360 | 0.360 |
| 38 | 3.987 | 11.606 | 0.187 | 1.284 | 0.333 | 0.400 |
| 39 | 3.987 | 11.606 | 0.233 | 1.204 | 0.333 | 0.400 |
| 40 | 5.919 | 12.129 | -0.376 | 1.516 | 0.360 | 0.360 |
| 41 | 5.835 | 12.129 | -0.505 | 1.635 | 0.350 | 0.450 |
| 42 | 5.113 | 12.577 | -0.164 | 1.699 | 0.240 | 0.360 |
| 43 | 5.987 | 13.188 | 0.081 | 1.563 | 0.385 | 0.462 |
| 44 | 5.987 | 11.374 | -0.008 | 1.789 | 0.462 | 0.385 |
| 45 | 3.030 | 13.000 | 0.615 | 1.651 | 0.462 | 0.462 |
| 46 | 3.988 | 11.157 | -0.108 | 1.799 | 0.400 | 0.360 |
| 47 | 3.877 | 11.288 | -0.275 | 1.267 | 0.250 | 0.321 |
| 48 | 3.988 | 13.078 | 0.448 | 1.613 | 0.478 | 0.391 |
| 49 | 3.072 | 13.000 | 0.322 | 1.655 | 0.333 | 0.370 |
| 50 | 5.985 | 11.952 | -0.416 | 1.262 | 0.280 | 0.400 |
| 51 | 5.874 | 11.105 | -0.133 | 1.548 | 0.259 | 0.407 |
| 52 | 3.030 | 10.987 | 0.630 | 1.728 | 0.435 | 0.348 |
| 53 | 3.989 | 11.676 | 0.373 | 1.330 | 0.367 | 0.400 |
| 54 | 3.988 | 11.157 | 0.044 | 1.548 | 0.440 | 0.440 |
| 55 | 3.988 | 13.078 | 0.248 | 1.400 | 0.440 | 0.320 |
| 56 | 4.988 | 11.752 | 0.220 | 1.569 | 0.440 | 0.440 |
| 57 | 5.111 | 11.200 | -0.068 | 1.608 | 0.400 | 0.360 |
| 58 | 3.031 | 11.393 | 0.096 | 1.595 | 0.333 | 0.333 |
| 59 | 3.988 | 11.157 | 0.329 | 1.537 | 0.458 | 0.375 |
| 60 | 4.835 | 11.390 | -0.725 | 1.543 | 0.250 | 0.333 |
| 61 | 3.986 | 11.053 | 0.546 | 1.779 | 0.417 | 0.458 |
| 62 | 4.987 | 11.279 | 0.260 | 1.245 | 0.400 | 0.400 |
| 63 | 3.030 | 13.000 | 0.465 | 1.791 | 0.231 | 0.385 |
| 64 | 4.987 | 11.279 | 0.466 | 1.674 | 0.414 | 0.379 |
| 65 | 4.986 | 11.700 | 0.535 | 1.523 | 0.462 | 0.385 |
| 66 | 4.987 | 10.979 | 0.200 | 1.715 | 0.379 | 0.379 |
| 67 | 3.988 | 13.078 | 0.433 | 1.443 | 0.333 | 0.467 |
| 68 | 4.834 | 11.677 | -0.184 | 1.312 | 0.211 | 0.368 |
| 69 | 2.988 | 10.987 | 0.600 | 1.472 | 0.400 | 0.360 |
| 70 | 4.030 | 11.157 | 0.009 | 1.725 | 0.435 | 0.391 |
| 71 | 4.987 | 13.141 | -0.053 | 1.818 | 0.467 | 0.333 |
| 72 | 3.114 | 13.000 | 0.390 | 1.439 | 0.429 | 0.429 |
| 73 | 3.988 | 11.157 | 0.481 | 1.280 | 0.385 | 0.423 |
| 74 | 3.032 | 12.400 | -0.280 | 1.833 | 0.333 | 0.400 |
| 75 | 3.988 | 11.676 | 0.440 | 1.411 | 0.433 | 0.400 |
| 76 | 4.987 | 11.279 | 0.232 | 1.760 | 0.320 | 0.400 |
| 77 | 3.988 | 13.078 | 0.404 | 1.734 | 0.440 | 0.360 |
| 78 | 3.835 | 10.947 | -0.721 | 1.168 | 0.211 | 0.421 |
| 79 | 3.985 | 10.892 | 0.194 | 1.707 | 0.222 | 0.444 |
| 80 | 4.987 | 11.279 | -0.022 | 1.726 | 0.435 | 0.348 |
| 81 | 5.988 | 12.434 | 0.140 | 1.361 | 0.400 | 0.333 |
| 82 | 4.070 | 11.428 | 0.323 | 1.642 | 0.455 | 0.455 |
| 83 | 3.030 | 10.987 | 0.200 | 1.788 | 0.348 | 0.348 |
| 84 | 2.988 | 11.288 | -0.067 | 1.375 | 0.400 | 0.400 |
| 85 | 2.989 | 10.946 | 0.233 | 1.391 | 0.467 | 0.367 |
| 86 | 5.987 | 13.188 | 0.027 | 1.446 | 0.462 | 0.423 |
| 87 | 4.031 | 11.495 | -0.254 | 1.404 | 0.385 | 0.385 |
| 88 | 2.989 | 11.572 | -0.078 | 1.215 | 0.389 | 0.333 |
| 89 | 3.988 | 13.078 | 0.550 | 1.781 | 0.308 | 0.462 |
| 90 | 3.030 | 10.987 | 0.512 | 1.587 | 0.480 | 0.360 |
| 91 | 5.031 | 12.129 | -0.256 | 1.785 | 0.280 | 0.400 |
| 92 | 5.030 | 11.752 | -0.018 | 1.811 | 0.409 | 0.455 |
| 93 | 2.988 | 10.987 | -0.314 | 1.816 | 0.286 | 0.500 |
| 94 | 5.987 | 13.188 | 0.338 | 1.700 | 0.476 | 0.381 |
| 95 | 4.988 | 11.279 | -0.116 | 1.839 | 0.440 | 0.360 |
| 96 | 4.986 | 11.146 | -0.069 | 1.674 | 0.345 | 0.414 |
| 97 | 5.985 | 11.311 | -0.332 | 1.659 | 0.455 | 0.409 |
| 98 | 5.987 | 13.188 | 0.513 | 1.611 | 0.458 | 0.417 |
| 99 | 4.987 | 11.279 | 0.222 | 1.651 | 0.296 | 0.407 |
| 100 | 3.988 | 11.157 | 0.252 | 1.587 | 0.400 | 0.400 |
| 101 | 3.987 | 11.606 | -0.253 | 1.697 | 0.267 | 0.467 |
| 102 | 3.030 | 13.000 | 0.592 | 1.750 | 0.417 | 0.500 |
| 103 | 3.988 | 11.157 | 0.312 | 1.404 | 0.417 | 0.417 |
| 104 | 4.073 | 12.412 | 0.345 | 1.719 | 0.300 | 0.400 |
| 105 | 2.989 | 11.572 | 0.496 | 1.584 | 0.360 | 0.320 |
| 106 | 3.031 | 12.130 | -0.229 | 1.708 | 0.429 | 0.429 |
| 107 | 3.988 | 11.157 | -0.093 | 1.706 | 0.467 | 0.400 |
| 108 | 2.990 | 12.400 | -0.387 | 1.459 | 0.200 | 0.333 |
| 109 | 3.835 | 10.913 | -0.173 | 1.569 | 0.364 | 0.409 |
| 110 | 3.987 | 11.324 | -0.057 | 1.363 | 0.333 | 0.381 |
| 111 | 4.030 | 11.157 | -0.167 | 1.572 | 0.400 | 0.333 |
| 112 | 5.984 | 11.541 | 0.185 | 1.704 | 0.300 | 0.500 |
| 113 | 5.987 | 13.188 | -0.439 | 1.627 | 0.391 | 0.435 |
| 114 | 4.986 | 11.700 | -0.047 | 1.603 | 0.467 | 0.333 |
| 115 | 3.988 | 12.403 | 0.100 | 1.223 | 0.444 | 0.444 |
| 116 | 2.989 | 10.987 | -0.100 | 1.576 | 0.385 | 0.385 |
| 117 | 4.030 | 11.157 | -0.042 | 1.822 | 0.292 | 0.333 |
| 118 | 4.987 | 13.141 | -0.052 | 1.801 | 0.360 | 0.320 |
| 119 | 4.030 | 13.078 | 0.367 | 1.415 | 0.481 | 0.333 |
| 120 | 5.987 | 13.188 | -0.333 | 1.600 | 0.400 | 0.400 |
| 121 | 5.987 | 13.188 | -0.313 | 1.608 | 0.400 | 0.400 |
| 122 | 5.987 | 13.188 | 0.200 | 1.840 | 0.400 | 0.467 |
| 123 | 3.988 | 13.078 | 0.050 | 1.754 | 0.400 | 0.500 |
| 124 | 4.680 | 10.947 | 0.467 | 1.822 | 0.389 | 0.389 |
| 125 | 4.987 | 11.812 | -0.715 | 1.356 | 0.269 | 0.385 |
| 126 | 4.987 | 11.812 | -0.715 | 1.356 | 0.269 | 0.385 |
| 127 | 5.680 | 11.106 | 0.541 | 1.175 | 0.471 | 0.412 |
| 128 | 5.987 | 12.426 | -0.263 | 1.476 | 0.375 | 0.438 |
| 129 | 5.987 | 12.426 | -0.321 | 1.435 | 0.357 | 0.429 |
| 130 | 5.987 | 12.426 | -0.592 | 1.514 | 0.417 | 0.417 |
| 131 | 3.988 | 13.078 | 0.043 | 1.428 | 0.429 | 0.429 |
| 132 | 5.832 | 11.519 | -0.062 | 1.409 | 0.308 | 0.462 |
| 133 | 4.030 | 11.157 | -0.074 | 1.705 | 0.304 | 0.348 |
| 134 | 5.113 | 13.141 | -0.355 | 1.835 | 0.300 | 0.350 |
| 135 | 3.988 | 11.157 | -0.820 | 1.735 | 0.467 | 0.467 |
| 136 | 5.028 | 12.162 | -0.775 | 1.595 | 0.200 | 0.450 |
| 137 | 3.877 | 11.393 | -0.793 | 1.670 | 0.333 | 0.400 |
| 138 | 2.989 | 11.572 | -0.185 | 1.343 | 0.231 | 0.462 |
| 139 | 4.988 | 12.278 | -0.463 | 1.431 | 0.250 | 0.438 |
| 140 | 2.988 | 11.813 | -0.031 | 1.605 | 0.385 | 0.462 |
| 141 | 5.028 | 12.162 | -0.359 | 1.358 | 0.294 | 0.353 |
| 142 | 4.987 | 13.141 | 0.169 | 1.669 | 0.462 | 0.385 |
| 143 | 3.987 | 11.606 | 0.554 | 1.575 | 0.385 | 0.462 |

**Table S10: Predicted PCSS descriptors during MTAMP generation.**

| No. | Net charge | Isoelectric point | Hydrophobicity | Amphiphilicity | Alpha helix | Beta sheet |
| --- | --- | --- | --- | --- | --- | --- |
| 1 | 2.773 | 12.615 | 2.177 | 1.152 | 0.327 | 0.591 |
| 2 | 5.857 | 12.319 | 0.256 | 1.272 | 0.521 | 0.509 |
| 3 | 6.887 | 13.606 | 0.359 | 1.261 | 0.360 | 0.464 |
| 4 | 7.130 | 13.997 | -0.190 | 1.469 | 0.195 | 0.480 |
| 5 | 7.136 | 12.093 | -0.359 | 2.226 | 0.531 | 0.471 |
| 6 | 3.696 | 12.970 | 0.585 | 1.435 | 0.326 | 0.318 |
| 7 | 11.390 | 12.782 | -1.742 | 1.620 | 0.512 | 0.255 |
| 8 | 6.646 | 13.612 | -0.566 | 1.344 | 0.405 | 0.199 |
| 9 | 3.757 | 13.097 | 1.516 | 1.463 | 0.161 | 0.624 |
| 10 | 2.684 | 9.708 | 0.603 | 1.452 | 0.173 | 0.753 |
| 11 | 2.587 | 11.390 | 0.868 | 0.965 | 0.112 | 0.543 |
| 12 | 9.137 | 14.134 | -1.665 | 1.865 | 0.158 | 0.361 |
| 13 | 8.061 | 13.232 | 0.294 | 1.813 | 0.521 | 0.391 |
| 14 | 9.168 | 13.879 | -1.880 | 1.514 | 0.245 | 0.335 |
| 15 | 2.721 | 9.977 | -0.419 | 1.247 | 0.400 | 0.219 |
| 16 | 7.613 | 13.869 | -0.124 | 1.617 | 0.584 | 0.583 |
| 17 | 11.092 | 11.979 | -0.192 | 1.539 | 0.425 | 0.369 |
| 18 | 7.702 | 13.143 | 0.005 | 1.317 | 0.423 | 0.459 |
| 19 | 5.319 | 13.404 | 1.233 | 1.433 | 0.258 | 0.522 |
| 20 | 7.288 | 12.946 | 0.817 | 1.212 | 0.451 | 0.586 |
| 21 | 6.891 | 11.145 | -0.581 | 2.096 | 0.250 | 0.512 |
| 22 | 8.749 | 13.978 | -2.078 | 1.916 | 0.287 | 0.196 |
| 23 | 6.366 | 12.915 | 0.539 | 1.649 | 0.396 | 0.592 |
| 24 | 5.669 | 12.983 | 0.820 | 1.221 | 0.424 | 0.442 |
| 25 | 6.283 | 11.594 | 0.595 | 1.291 | 0.283 | 0.399 |
| 26 | 10.064 | 13.789 | -0.541 | 1.754 | 0.261 | 0.351 |
| 27 | 5.338 | 10.916 | -1.164 | 1.096 | 0.383 | 0.461 |
| 28 | 8.470 | 12.468 | -1.040 | 1.512 | 0.706 | 0.384 |
| 29 | 3.214 | 13.014 | 1.205 | 1.081 | 0.138 | 0.469 |
| 30 | 3.307 | 9.078 | 1.073 | 1.385 | 0.068 | 0.377 |
| 31 | 7.287 | 10.971 | -0.788 | 1.581 | 0.448 | 0.372 |
| 32 | 3.765 | 13.707 | 1.931 | 1.612 | 0.552 | 0.620 |
| 33 | 6.558 | 14.020 | -0.518 | 0.881 | 0.133 | 0.272 |
| 34 | 3.290 | 11.549 | 1.407 | 1.214 | 0.117 | 0.681 |
| 35 | 6.154 | 12.389 | 0.853 | 1.417 | 0.493 | 0.466 |
| 36 | 4.242 | 11.739 | 1.100 | 1.136 | 0.523 | 0.626 |
| 37 | 7.655 | 10.103 | -1.142 | 1.527 | 0.366 | 0.335 |
| 38 | 5.685 | 13.932 | -0.134 | 1.175 | 0.230 | 0.373 |
| 39 | 5.264 | 12.537 | 0.964 | 1.694 | 0.239 | 0.494 |
| 40 | 11.038 | 14.200 | -1.277 | 1.451 | 0.455 | 0.356 |
| 41 | 4.632 | 12.175 | 1.016 | 1.576 | 0.352 | 0.683 |
| 42 | 5.550 | 14.039 | 0.083 | 1.584 | 0.232 | 0.388 |
| 43 | 7.182 | 13.580 | -0.469 | 1.395 | 0.499 | 0.373 |
| 44 | 3.647 | 12.731 | 2.105 | 1.115 | 0.441 | 0.632 |
| 45 | 3.029 | 12.693 | 0.650 | 1.161 | 0.604 | 0.294 |
| 46 | 7.984 | 14.043 | -1.125 | 2.046 | 0.282 | 0.321 |
| 47 | 2.312 | 7.913 | 0.599 | 1.289 | 0.155 | 0.364 |
| 48 | 6.791 | 13.273 | -0.810 | 1.147 | 0.467 | 0.256 |
| 49 | 0.717 | 11.640 | 0.895 | 0.945 | 0.260 | 0.394 |
| 50 | 5.478 | 12.281 | -0.234 | 1.319 | 0.179 | 0.361 |
| 51 | 6.218 | 11.014 | 0.245 | 1.363 | 0.116 | 0.459 |
| 52 | 2.920 | 9.578 | 1.704 | 0.778 | 0.611 | 0.452 |
| 53 | 5.507 | 13.528 | 0.498 | 1.656 | 0.308 | 0.434 |
| 54 | 4.981 | 12.235 | 1.073 | 1.302 | 0.518 | 0.640 |
| 55 | 4.023 | 13.379 | 0.939 | 0.830 | 0.553 | 0.290 |
| 56 | 8.168 | 12.934 | -0.014 | 1.754 | 0.501 | 0.414 |
| 57 | 7.631 | 13.213 | 0.356 | 1.351 | 0.508 | 0.495 |
| 58 | 2.705 | 12.859 | 2.261 | 1.495 | 0.288 | 0.769 |
| 59 | 7.617 | 12.998 | -0.980 | 1.089 | 0.505 | 0.129 |
| 60 | 10.035 | 12.473 | -2.817 | 1.480 | 0.166 | 0.088 |
| 61 | 5.137 | 11.858 | -0.240 | 1.299 | 0.525 | 0.357 |
| 62 | 3.685 | 8.371 | 0.746 | 1.444 | 0.346 | 0.230 |
| 63 | 4.135 | 13.263 | 0.649 | 1.373 | 0.124 | 0.463 |
| 64 | 4.291 | 11.507 | 1.359 | 1.402 | 0.437 | 0.475 |
| 65 | 8.809 | 12.881 | -0.122 | 1.083 | 0.560 | 0.339 |
| 66 | 13.460 | 13.262 | -0.873 | 1.845 | 0.594 | 0.307 |
| 67 | 2.492 | 12.866 | 1.646 | 1.045 | 0.351 | 0.595 |
| 68 | 6.712 | 10.861 | -1.326 | 1.468 | 0.243 | 0.340 |
| 69 | 4.862 | 13.234 | 1.149 | 1.150 | 0.612 | 0.524 |
| 70 | 3.736 | 11.530 | 0.348 | 1.407 | 0.488 | 0.505 |
| 71 | 5.174 | 13.150 | 0.060 | 1.276 | 0.496 | 0.310 |
| 72 | 4.999 | 13.388 | 0.182 | 1.834 | 0.825 | 0.434 |
| 73 | 7.745 | 13.050 | -0.156 | 1.393 | 0.495 | 0.336 |
| 74 | 6.835 | 13.177 | -1.766 | 1.417 | 0.470 | 0.257 |
| 75 | 4.609 | 11.662 | 1.059 | 1.434 | 0.470 | 0.363 |
| 76 | 6.006 | 11.198 | 0.303 | 1.528 | 0.311 | 0.331 |
| 77 | 5.889 | 13.189 | 0.300 | 2.087 | 0.535 | 0.314 |
| 78 | 4.054 | 11.880 | -0.806 | 1.204 | 0.113 | 0.498 |
| 79 | 4.483 | 11.293 | 0.921 | 1.510 | 0.236 | 0.574 |
| 80 | 8.798 | 13.131 | -0.069 | 1.629 | 0.675 | 0.379 |
| 81 | 7.572 | 13.976 | -0.548 | 1.005 | 0.389 | 0.199 |
| 82 | 3.817 | 12.181 | 1.342 | 1.910 | 0.706 | 0.652 |
| 83 | 6.850 | 12.929 | 0.157 | 1.442 | 0.450 | 0.401 |
| 84 | 5.697 | 13.057 | 0.057 | 0.993 | 0.433 | 0.513 |
| 85 | 3.739 | 11.412 | -0.058 | 1.843 | 0.653 | 0.321 |
| 86 | 8.618 | 13.301 | -0.222 | 1.807 | 0.543 | 0.399 |
| 87 | 3.474 | 11.823 | -0.570 | 0.871 | 0.199 | 0.252 |
| 88 | 4.031 | 13.070 | 0.697 | 1.307 | 0.445 | 0.314 |
| 89 | 2.948 | 12.985 | 2.146 | 1.866 | 0.329 | 0.721 |
| 90 | 2.754 | 12.592 | 1.828 | 1.229 | 0.418 | 0.512 |
| 91 | 10.811 | 13.515 | -1.074 | 1.504 | 0.394 | 0.356 |
| 92 | 2.948 | 11.193 | 1.769 | 1.435 | 0.264 | 0.756 |
| 93 | 2.736 | 10.280 | -0.225 | 1.736 | 0.360 | 0.514 |
| 94 | 7.400 | 13.646 | 0.126 | 1.635 | 0.433 | 0.419 |
| 95 | 3.577 | 11.240 | 0.825 | 1.648 | 0.363 | 0.452 |
| 96 | 7.339 | 12.575 | 0.365 | 1.619 | 0.470 | 0.533 |
| 97 | 6.552 | 10.946 | -0.302 | 1.281 | 0.431 | 0.540 |
| 98 | 5.301 | 13.085 | 0.410 | 1.499 | 0.398 | 0.403 |
| 99 | 6.877 | 12.135 | 0.275 | 1.457 | 0.382 | 0.486 |
| 100 | 2.056 | 10.993 | -0.011 | 0.875 | 0.329 | 0.159 |
| 101 | 6.921 | 13.603 | -0.537 | 1.684 | 0.422 | 0.370 |
| 102 | 4.290 | 13.491 | 0.556 | 1.486 | 0.506 | 0.488 |
| 103 | 2.110 | 10.913 | 2.515 | 1.346 | 0.466 | 0.766 |
| 104 | 4.810 | 14.011 | 0.548 | 1.598 | 0.253 | 0.551 |
| 105 | 3.218 | 12.176 | 1.526 | 0.933 | 0.380 | 0.477 |
| 106 | 3.233 | 11.253 | -1.664 | 1.057 | 0.451 | 0.268 |
| 107 | 8.597 | 12.480 | -1.177 | 1.298 | 0.735 | 0.375 |
| 108 | 4.663 | 13.580 | -1.014 | 1.080 | 0.003 | 0.264 |
| 109 | 7.329 | 12.039 | 0.052 | 1.410 | 0.491 | 0.567 |
| 110 | 8.341 | 13.831 | -0.497 | 1.430 | 0.268 | 0.326 |
| 111 | 2.790 | 12.839 | 1.304 | 1.260 | 0.336 | 0.456 |
| 112 | 6.128 | 12.063 | 0.428 | 1.582 | 0.241 | 0.625 |
| 113 | 7.783 | 13.843 | -0.738 | 1.727 | 0.411 | 0.412 |
| 114 | 4.700 | 13.100 | 0.529 | 1.381 | 0.444 | 0.417 |
| 115 | 8.619 | 13.788 | -0.951 | 1.203 | 0.541 | 0.332 |
| 116 | 2.105 | 10.034 | 0.579 | 1.341 | 0.285 | 0.462 |
| 117 | 0.505 | 9.251 | 0.578 | 1.150 | 0.117 | 0.391 |
| 118 | 3.820 | 12.596 | -0.107 | 1.149 | 0.345 | 0.249 |
| 119 | 5.113 | 14.004 | -0.385 | 1.197 | 0.703 | 0.215 |
| 120 | 7.481 | 13.629 | -0.650 | 1.619 | 0.567 | 0.417 |
| 121 | 6.616 | 14.071 | -0.410 | 1.836 | 0.495 | 0.390 |
| 122 | 5.394 | 13.997 | 1.015 | 1.525 | 0.483 | 0.648 |
| 123 | 6.700 | 13.485 | -0.427 | 2.251 | 0.538 | 0.472 |
| 124 | 6.355 | 12.148 | 0.596 | 1.341 | 0.371 | 0.385 |
| 125 | 6.441 | 11.415 | -1.054 | 1.549 | 0.292 | 0.365 |
| 126 | 9.218 | 13.099 | -1.877 | 1.186 | 0.445 | 0.294 |
| 127 | 3.575 | 9.259 | 0.880 | 1.401 | 0.521 | 0.287 |
| 128 | 5.858 | 13.583 | 0.423 | 1.194 | 0.339 | 0.653 |
| 129 | 12.858 | 13.880 | -1.900 | 1.367 | 0.276 | 0.246 |
| 130 | 9.070 | 13.786 | -1.835 | 1.728 | 0.601 | 0.290 |
| 131 | 6.403 | 13.634 | -1.446 | 2.335 | 0.498 | 0.318 |
| 132 | 7.109 | 12.127 | 0.482 | 1.662 | 0.589 | 0.607 |
| 133 | 7.115 | 13.331 | 0.020 | 1.093 | 0.340 | 0.434 |
| 134 | 5.599 | 13.769 | 0.042 | 1.585 | 0.127 | 0.482 |
| 135 | 5.132 | 11.990 | -0.675 | 1.795 | 0.517 | 0.500 |
| 136 | 7.413 | 13.989 | -0.933 | 1.324 | 0.301 | 0.461 |
| 137 | 4.395 | 11.491 | -0.902 | 2.089 | 0.317 | 0.543 |
| 138 | 6.901 | 14.262 | -2.189 | 1.254 | 0.293 | 0.341 |
| 139 | 6.477 | 13.377 | -0.515 | 1.047 | 0.184 | 0.403 |
| 140 | 3.849 | 11.668 | 0.132 | 1.452 | 0.613 | 0.577 |
| 141 | 9.085 | 13.153 | -1.662 | 0.844 | 0.617 | 0.151 |
| 142 | 6.368 | 13.496 | 0.695 | 1.623 | 0.553 | 0.502 |

**Table S11: WS of sequences generated by the GenMTAMP model.**

| Peptide names | Sequences | Scores | Grades |
| --- | --- | --- | --- |
| MTAMP001 | KLWWFFNNKIKIKRKIKIKG | 1.00000 | 1 |
| MTAMP002 | RLILVPIVALLVIRRLVKKRKRR | 1.00000 | 1 |
| MTAMP003 | RLLLALIVRRRLRL | 1.00000 | 1 |
| MTAMP004 | GWKSWAKRALAWWLL | 1.00000 | 1 |
| MTAMP005 | VVGVGIIAGVVKGGG | 1.00000 | 1 |
| MTAMP006 | FIIIISKLKLLKL | 1.00000 | 1 |
| MTAMP007 | FWPRKLKLRRKRRLKRRRL | 1.00000 | 1 |
| MTAMP008 | GKLRRKRRFILRLGKR | 1.00000 | 1 |
| MTAMP009 | GLDLKIIAKVKLVLIAVKLVL | 0.99999 | 2 |
| MTAMP010 | GKVVLSIKAAGAKGIVIIIKSISCKS | 0.99992 | 2 |
| MTAMP011 | GVIFKVFKVGKKKGIIKGICKCGIKS | 0.99991 | 2 |
| MTAMP012 | FKKKPIKLK | 0.99979 | 2 |
| MTAMP013 | IGGKKVKVGKGVKIKLIKGLK | 0.99965 | 2 |
| MTAMP014 | GRGIKKIGRIKGILR | 0.99963 | 2 |
| MTAMP015 | KKRKKKRLQRKLRRRKKKR | 0.99929 | 2 |
| MTAMP016 | VLFWIHIVKVVVKIKKVLLIRLR | 0.99890 | 2 |
| MTAMP017 | RYLYLRLWKRL | 0.99716 | 2 |
| MTAMP018 | FGSSKKKKKLLGGKLIVAKG | 0.99655 | 2 |
| MTAMP019 | ILISIGSISIGG | 0.99619 | 2 |
| MTAMP020 | IALLGLLKAL | 0.99524 | 2 |
| MTAMP021 | KWIWVVLLKLALKVGLLAIKLAKQ | 0.99373 | 2 |
| MTAMP022 | RKVSVRISSGKGRRGSGGK | 0.99367 | 2 |
| MTAMP023 | KLVLRCRIKC | 0.99304 | 2 |
| MTAMP024 | GLPLLFAKKTTGTLLLFLTFK | 0.99245 | 2 |
| MTAMP025 | GAIISLLAKGGSKAAKSAKGK | 0.99114 | 2 |
| MTAMP026 | GLLSRRAAIALLAVIVIAK | 0.99109 | 2 |
| MTAMP027 | VCIRVCK | 0.99028 | 2 |
| MTAMP028 | VLLGGKGLKAGAKKKKKALG | 0.98912 | 2 |
| MTAMP029 | RVRKRRIRKRIAAC | 0.98795 | 2 |
| MTAMP030 | GLGSIIGIKKALKGGKGIIGIICGGG | 0.98648 | 2 |
| MTAMP031 | GFSLKINGNKGIIKKKGGKC | 0.98635 | 2 |
| MTAMP032 | LGPGRKKSRRLIRGGRAA | 0.98562 | 2 |
| MTAMP033 | GRRIRKIRKRKKGRAR | 0.98492 | 2 |
| MTAMP034 | RVIRRRVVIV | 0.98436 | 2 |
| MTAMP035 | KIWIKFSK | 0.98425 | 2 |
| MTAMP036 | GFIVKKKYVRRVIRKRVLVYK | 0.98354 | 2 |
| MTAMP037 | RIRKVRIKKRVV | 0.98256 | 2 |
| MTAMP038 | AIGGKKIGLGAIAKIAIIIKSSKQ | 0.98091 | 2 |
| MTAMP039 | LWLIKKKLLKKGKLLLKKKLLNK | 0.98040 | 2 |
| MTAMP040 | GKLRRLRARRPPILILRKQ | 0.97914 | 2 |
| MTAMP041 | ILIKKKVRRKIFII | 0.97827 | 2 |
| MTAMP042 | VFLAIVVARLRLVIKIRKRR | 0.97767 | 2 |
| MTAMP043 | FRPRKLKRLKLKKKKCCR | 0.97340 | 2 |
| MTAMP044 | GVRIRPLPRLIPIPIKRCRK | 0.97319 | 2 |
| MTAMP045 | KRKRKKKCKIIC | 0.97297 | 2 |
| MTAMP046 | LILALIIKKLVLVKVLKIICCC | 0.97205 | 2 |
| MTAMP047 | IGLKKVIAKVKLGLLLSIIAAKSKGG | 0.97077 | 2 |
| MTAMP048 | FVKVLVKLC | 0.96985 | 2 |
| MTAMP049 | RFRLVVRFVRIFRRCKKC | 0.96811 | 2 |
| MTAMP050 | GFWWLKLKKAVVKKKAKVAVARLKKRQ | 0.96485 | 2 |
| MTAMP051 | AVGFFIIKKVGIVLVLILKLKKKKC | 0.95869 | 2 |
| MTAMP052 | GWGSFLKLAAGVAKGAAAGIKIAKLSG | 0.95817 | 2 |
| MTAMP053 | IGIKGGGLGAAAAKVKLKKKKL | 0.95806 | 2 |
| MTAMP054 | FFIIIKIVGKKKIVKILK | 0.95629 | 2 |
| MTAMP055 | GLILVKRIRIVKVRKVIIKKKKVGG | 0.95494 | 2 |
| MTAMP056 | GILDKNKGIVVIKKVVVVGIGK | 0.95074 | 2 |
| MTAMP057 | LRKIRFFRFLFK | 0.94520 | 2 |
| MTAMP058 | IIWTKKARKLLLRTIRRYYYKLYR | 0.94502 | 2 |
| MTAMP059 | VVFKKWKKKVVKKFKIK | 0.94458 | 2 |
| MTAMP060 | IIWLFILKVGVKVGLLGGAGVGKGKQQ | 0.94353 | 2 |
| MTAMP061 | KIWKIKGGGLGLGLA | 0.94199 | 2 |
| MTAMP062 | AVVGLAVIVGVIKAAKKG | 0.94133 | 2 |
| MTAMP063 | LKLKKVLKKKLALKA | 0.94017 | 2 |
| MTAMP064 | APSLAILSLKKGLKL | 0.93894 | 2 |
| MTAMP065 | CCFKKRRKRGRSGCKK | 0.93776 | 2 |
| MTAMP066 | LLKRRLIRKLKKIRR | 0.93589 | 2 |
| MTAMP067 | RGRIIVVVRGVL | 0.93550 | 2 |
| MTAMP068 | ALALLAAFALKFKIIKKKKCKC | 0.93505 | 2 |
| MTAMP069 | LAALLQLQRALLLGL | 0.93424 | 2 |
| MTAMP070 | LRRLFKLFLTKLFLY | 0.93197 | 2 |
| MTAMP071 | VKVLKKLLIVI | 0.93138 | 2 |
| MTAMP072 | KIIIGIGYGCKKSGKRRRGKKK | 0.93029 | 2 |
| MTAMP073 | FFILLIALKLAPAPLIKFIFR | 0.92941 | 2 |
| MTAMP074 | GKFVKKFVVVVVGGGILVIK | 0.92686 | 2 |
| MTAMP075 | WILFTITTKKGVGLLLAVALGATALLA | 0.92244 | 2 |
| MTAMP076 | KFWWKKKVVKVIKRVKRRYK | 0.91807 | 2 |
| MTAMP077 | GGIKKRVKIIVKVKIVFFI | 0.91801 | 2 |
| MTAMP078 | KKKKLRRKRWLLKKYK | 0.91687 | 2 |
| MTAMP079 | FGLLLSLGHAGVISGHVIIQQ | 0.91655 | 2 |
| MTAMP080 | FFLILKKAAGVALILFSIISKSTC | 0.91623 | 2 |
| MTAMP081 | GAGAAIGAIVAIKR | 0.91458 | 2 |
| MTAMP082 | KRWWWIRKKRRRILF | 0.91257 | 2 |
| MTAMP083 | IPISSGAPPALKKFK | 0.91039 | 2 |
| MTAMP084 | GGGLILGKGAGIAGIGIIIA | 0.90475 | 2 |
| MTAMP085 | LKLIALALKLLLKVVLVL | 0.90417 | 2 |
| MTAMP086 | GAKKGKKKIKAIGLIAAKLK | 0.90367 | 2 |
| MTAMP087 | IKKKRKKKKIIRYYGY | 0.90297 | 2 |
| MTAMP088 | ILRLCLLCKR | 0.90190 | 2 |
| MTAMP089 | GLSLAKIKKLKKGSCILICKKLTC | 0.90080 | 2 |
| MTAMP090 | ALKIAAARLLLL | 0.89791 | 3 |
| MTAMP091 | GLSAILIVKKGGLLAVLSKKSQ | 0.89433 | 3 |
| MTAMP092 | SIVIKLGGGKAGVVLAGSSK | 0.89030 | 3 |
| MTAMP093 | IFVFVIRRRGRIGGKK | 0.88961 | 3 |
| MTAMP094 | VWWLRRRQQQRRRKRGR | 0.88766 | 3 |
| MTAMP095 | GLGDIVKGKKVVKLKLKKCKLK | 0.88738 | 3 |
| MTAMP096 | GLLKLLAAKCKKCC | 0.88605 | 3 |
| MTAMP097 | GKKKKLLGGKGKLK | 0.88558 | 3 |
| MTAMP098 | GFGGLILALAKVCIKACKK | 0.88187 | 3 |
| MTAMP099 | GGGHGIVGIHIR | 0.88064 | 3 |
| MTAMP100 | LLLLLLRRKFIKKFCKKKKK | 0.87553 | 3 |
| MTAMP101 | GILSAILKAALKGALLIVAAAKCKK | 0.87446 | 3 |
| MTAMP102 | LFHGLFFLVRVLIITRLRKKK | 0.87395 | 3 |
| MTAMP103 | GLSSITIVGVGLLGGILVKGKGGS | 0.87250 | 3 |
| MTAMP104 | LFRFRIIFKFKLRG | 0.87222 | 3 |
| MTAMP105 | GLLRLIRKFIRIKIKIK | 0.86509 | 3 |
| MTAMP106 | LIARIR | 0.86165 | 3 |
| MTAMP107 | VKGGGGGIGKVGKKKAK | 0.85821 | 3 |
| MTAMP108 | GLASSKGLAAKLALLAKSQQL | 0.85504 | 3 |
| MTAMP109 | GLVIKVRIIKIILLK | 0.85471 | 3 |
| MTAMP110 | KFAAKKKRLIRAIKIK | 0.85227 | 3 |
| MTAMP111 | RWSSIVIRRRVIVVIVIRGGISLRG | 0.85004 | 3 |
| MTAMP112 | GGSGGKGKGLKKLAGAKVAGK | 0.84834 | 3 |
| MTAMP113 | KIKIKLAGLGAIILGKSIL | 0.84764 | 3 |
| MTAMP114 | IVLKSIILLIGG | 0.84688 | 3 |
| MTAMP115 | ILIINIKKKKLI | 0.84428 | 3 |
| MTAMP116 | RLPYYRLYFVFYLLFGL | 0.83940 | 3 |
| MTAMP117 | IFNWVVIIFLIKLIF | 0.83811 | 3 |
| MTAMP118 | FLRLRKKKKKLLLLR | 0.83499 | 3 |
| MTAMP119 | GWPKAKLLGAALLIIIYL | 0.83136 | 3 |
| MTAMP120 | GLWKIIKIIVLILKRIKLKLKKIK | 0.83097 | 3 |
| MTAMP121 | KKRKLIKRLKRALI | 0.82653 | 3 |
| MTAMP122 | FFWIFKLTAATKAVATLIGGLAVGQGG | 0.82632 | 3 |
| MTAMP123 | FGLKKGAKKAVGKGGAG | 0.82448 | 3 |
| MTAMP124 | VRGKGWVVVGRGRGGR | 0.82273 | 3 |
| MTAMP125 | LLPDVKGGKVKGAVVKTKKVL | 0.82010 | 3 |
| MTAMP126 | RLRRRKIRLIVKVRK | 0.81874 | 3 |
| MTAMP127 | GSILGSLKIRRRLA | 0.8071 | 3 |
| MTAMP128 | RIRRRIRFIRKFFRR | 0.80197 | 3 |
| MTAMP129 | ALARCCKCRLLRCGKK | 0.80125 | 3 |
| MTAMP130 | KLLVIVKLALKLALIKALALKKG | 0.80000 | 3 |
| MTAMP131 | ALILILAVAAGLKLLACKTGKC | 0.79712 | 4 |
| MTAMP132 | FPLALRRKKKRRK | 0.78138 | 4 |
| MTAMP133 | AFIRFIKGKRKGLILKKKGRK | 0.78078 | 4 |
| MTAMP134 | WLFKRYFIRRFIGG | 0.78048 | 4 |
| MTAMP135 | ICLCRLVAVCRL | 0.77926 | 4 |
| MTAMP136 | LLFKFALKLLVILKAILILKIC | 0.77846 | 4 |
| MTAMP137 | AGLIKGKKIIKLKRGR | 0.77161 | 4 |
| MTAMP138 | GRRWRGKGGIRARRKA | 0.75859 | 4 |
| MTAMP139 | GFYYWWYWWKKWWKKVRRLK | 0.75418 | 4 |
| MTAMP140 | KIKVKVKKKVLLKG | 0.74140 | 4 |
| MTAMP141 | KRRVVRFFRR | 0.74130 | 4 |
| MTAMP142 | ALLSVKIVVGAIILKALCLKG | 0.73913 | 4 |

**Table S12: MIC values of MTAMP003, MTAMP004, and Magainin II against Gram-positive bacteria and fungi.**

| Peptide names |  | MIC (µg/mL) |  |
| --- | --- | --- | --- |
|  | *S. aureus* ATCC 25923 | *B. subtilis* ATCC 9372 | *C. albicans* ATCC 10231 |
| MTAMP003 | 8 | 4 | 256 |
| MTAMP004 | 4 | 4 | 256 |
| Magainin II | 4 | 4 | 128 |

**Table S13: LC-MS and HPLC results of MTAMPs based on Fmoc-SPPS method.**

| Peptide names | Sequences | Ideal MW | True MW | MW error | Target peak area | Total peak area | Purity |
| --- | --- | --- | --- | --- | --- | --- | --- |
| MTAMP001 | KLWWFFNNKIKIKRKIKIKG | 2589.218 | 2588.90 | 0.01228% | 6584060 | 6690269 | 98.412% |
| MTAMP002 | RLILVPIVALLVIRRLVKKRKRR | 2809.646 | 2810.40 | 0.02684% | 27300642 | 26347038 | 96.507% |
| MTAMP003 | RLLLALIVRRRLRL | 1761.272 | 1762.05 | 0.04417% | 3599114 | 3723304 | 96.665% |
| MTAMP004 | GWKSWAKRALAWWLL | 1872.227 | 1871.70 | 0.02815% | 19302037 | 18625055 | 96.493% |
| MTAMP005 | VVGVGIIAGVVKGGG | 1281.546 | 1282.10 | 0.04323% | 6926495 | 6586102 | 95.086% |
| MTAMP006 | FIIIISKLKLLKL | 1542.045 | 1543.05 | 0.06517% | 24041846 | 23257842 | 96.739% |
| MTAMP007 | FWPRKLKLRRKRRLKRRRL | 2663.331 | 2664.60 | 0.04765% | 7921096 | 7542556 | 95.221% |
| MTAMP008 | GKLRRKRRFILRLGKR | 2053.559 | 2054.40 | 0.04095% | 15219183 | 14825911 | 97.416% |

**References**

**1.** Gao H., et al. DLFea4AMPGen de novo design of antimicrobial peptides by integrating features learned from deep learning models. *Nat. Commun.* **16**, 9134 (2025).

**2.** Jiang Z. H., et al. AI-guided design of antimicrobial peptide hydrogels for precise treatment of drug-resistant bacterial infections. *Adv. Mater.* **37**, 2500043 (2025).

**3.** Du R. B., et al. Uncovering encrypted antimicrobial peptides in health-associated Lactobacillaceae by large-scale genomics and machine learning. *Microbiome* **13**, 151 (2025).

**4.** Bhangu S. K., et al. Machine learning-assisted prediction and generation of antimicrobial peptides. *Small Sci.* **5**, 2400579 (2025).

**5.** Tan L. D., et al. SQ-DiffuPep: A multimodal information-guided quantitative latent diffusion model for antimicrobial peptide discovery. *Inf. Fusion* **121**, 103119 (2025).

**6.** Wang X.-F., et al. ProT-Diff: A modularized and efficient strategy for de novo generation of antimicrobial peptide sequences by integrating protein language and diffusion models. *Adv. Sci.* **11**, 2406305 (2024).

**7.** Das P., et al. Accelerated antimicrobial discovery via deep generative models and molecular dynamics simulations. *Nat. Biomed. Eng.* **5**, 613-623 (2021).

**8.** Cao Q. S., et al. Designing antimicrobial peptides using deep learning and molecular dynamic simulations. *Brief. Bioinform.* **24**, bbad058 (2023).

**9.** Hu M. Y., et al. Development of targeted antimicrobial peptides for Escherichia coli: Combining phage display and rational design for food safety application. *Food Chem.* **470**, 142685 (2025).

**10.** Zhang M. N., et al. Cathelicidin AS-12W derived from the alligator sinensis and its antimicrobial activity against drug-resistant Gram-negative bacteria in vitro and in vivo. *Probiotics Antimicrob. Proteins* **17**, 2995-3013 (2025).

**11.** Dean S. N., Alvarez J. a. E., Zabetakis D., Walper S. A., Malanoski A. P. PepVAE: Variational autoencoder framework for antimicrobial peptide generation and activity prediction. *Front. Microbiol.* **12**, 725727 (2021).

**12.** Wang Y. K., Li M. L., Huang F., Qiu M. Y., Zhang W. Controllable generation of pathogen-specific antimicrobial peptides through knowledge-aware prompt diffusion model. *Adv. Sci.* **12**, e07457 (2025).

**13.** Wang J., et al. Discovery of antimicrobial peptides with notable antibacterial potency by an LLM-based foundation model. *Sci. Adv.* **11**, eads8932 (2025).

**14.** Szymczak P., et al. Discovering highly potent antimicrobial peptides with deep generative model HydrAMP. *Nat. Commun.* **14**, 1453 (2023).

1. *Corresponding author. Email: dawen.sun@ucd.ie. URLs: http://www.ucd.ie/refrig; http://www.ucd.ie/sun. [↑](#footnote-ref-1)
